# Revealing Molecular Mechanisms by Integrating High-Dimensional Functional Screens with Protein Interaction Data

**DOI:** 10.1371/journal.pcbi.1003801

**Published:** 2014-09-04

**Authors:** Angela Simeone, Giovanni Marsico, Claudio Collinet, Thierry Galvez, Yannis Kalaidzidis, Marino Zerial, Andreas Beyer

**Affiliations:** 1 Biotechnology Center, TU Dresden, Dresden, Germany; 2 Max Planck Institute for Molecular Cell Biology and Genetics, Dresden, Germany; 3 Belozersky Institute of Physico-Chemical Biology & Faculty of Bioengineering and Bioinformatics, Moscow State University, Moscow, Russia; 4 Center for Regenerative Therapy, Dresden, Germany; 5 University of Cologne, Cologne, Germany; University of Toronto, Canada

## Abstract

Functional genomics screens using multi-parametric assays are powerful approaches for identifying genes involved in particular cellular processes. However, they suffer from problems like noise, and often provide little insight into molecular mechanisms. A bottleneck for addressing these issues is the lack of computational methods for the systematic integration of multi-parametric phenotypic datasets with molecular interactions. Here, we present Integrative Multi Profile Analysis of Cellular Traits (IMPACT). The main goal of IMPACT is to identify the most consistent phenotypic profile among interacting genes. This approach utilizes two types of external information: sets of related genes (IMPACT-sets) and network information (IMPACT-modules). Based on the notion that interacting genes are more likely to be involved in similar functions than non-interacting genes, this data is used as a prior to inform the filtering of phenotypic profiles that are similar among interacting genes. IMPACT-sets selects the most frequent profile among a set of related genes. IMPACT-modules identifies sub-networks containing genes with similar phenotype profiles. The statistical significance of these selections is subsequently quantified *via* permutations of the data. IMPACT (1) handles multiple profiles per gene, (2) rescues genes with weak phenotypes and (3) accounts for multiple biases e.g. caused by the network topology. Application to a genome-wide RNAi screen on endocytosis showed that IMPACT improved the recovery of known endocytosis-related genes, decreased off-target effects, and detected consistent phenotypes. Those findings were confirmed by rescreening 468 genes. Additionally we validated an unexpected influence of the IGF-receptor on EGF-endocytosis. IMPACT facilitates the selection of high-quality phenotypic profiles using different types of independent information, thereby supporting the molecular interpretation of functional screens.

## Author Summary

Genome-scale functional genomics screens are important tools for investigating the function of genes. Technological progress allows for the simultaneous measurement of multiple parameters quantifying the response of cells to gene perturbations such as RNA interference. Such multi-dimensional screens provide rich data, but there is a lack of computational methods for interpreting these complex measurements. We have developed two computational methods that combine the data from multi-dimensional functional genomics screens with protein interaction information. These methods search for phenotype patterns that are consistent among interacting genes. Thereby, we could reduce the noise in the data and facilitate the mechanistic interpretation of the findings. The performance of the methods was demonstrated through application to a genome-wide screen studying endocytosis. Subsequent experimental validation demonstrated the improved detection of phenotypic profiles through the use of protein interaction data. Our analysis revealed unexpected roles of specific network modules and protein complexes with respect to endocytosis. Detailed follow-up experiments investigating the dynamics of endocytosis uncovered crosstalk between the cancer-related EGF and IGF pathways with so far unknown effects on endocytosis and cargo trafficking.

## Introduction

Genome-scale functional genetics screens using technologies such as RNA interference (RNAi) have recently started to generate high-dimensional datasets by measuring either the same parameter in different cell lines [1], [2] or different features in the same cell line [3]–[5].

Such high-dimensionality improves the phenotypic specificity but, at the same time, increases the complexity of the analysis: the knock-down of two genes may have a similar phenotype on one parameter but yield different results on another. This poses a substantial challenge for the mechanistic interpretation of such screens [6], [7].

Furthermore, it has been noticed that targeting the same gene with different siRNAs can lead to conflicting results [3]. This ambiguity is caused by the additive influence of noise in the assay and off-target effects (OTEs). OTEs occur when the detected phenotype is due to interactions between the silencing molecules and genes other than the intended target [8], [9]. Thus, OTEs complicate the functional interpretation of RNAi screens and may lead to spurious gene annotation. Even though OTEs can be reduced in small-scale studies (e.g. by gene rescue experiments), it is very difficult to completely avoid them in large-scale genomic screens [10]. Consequently, it is often impossible to unambiguously assign the assay readout to a target gene without considering additional information. Note that frequently even replicate measurements using the same siRNA can be inconsistent, which is not necessarily an indication of bad experimental skills, but rather a problem intrinsic to the complexity of genome-wide screens [11], [12].

Previous work has shown that integrating independent information, such as protein interaction networks with RNAi screening data removes noise and improves the elucidation of molecular mechanisms [7], [4], [13]–[18]. These approaches exploit the fact that phenotypes that are observed consistently across a set of interacting genes are less likely to be noise. Hence, interaction data can be used to filter for genes that are more likely true positives. However, existing studies have not sufficiently addressed the problem of high-dimensional phenotypes nor the ambiguity of results from different siRNAs [7], [13]–[16]. The issue of multiple profiles per gene is relevant for studies performing replicate measurements with the same siRNAs, using different siRNAs per gene, as well as studies conducting functional assays on cells from multiple individuals/different cell lines.

Further, published studies often rely on first defining an arbitrary cut-off value for selecting ‘hit genes’ and subsequently interpreting their phenotypes using prior information [15], [16], [19]. Such approach is problematic because genes falling just below the threshold may be rejected even though their phenotype is consistent with interacting genes. Instead, it has been suggested to infer sets of relevant genes by first integrating the phenotype data with network information without any threshold and then simultaneously accounting for strength of the phenotype and its consistency in the network [20]. Two classes of such methods exist: methods of the first class assume one phenotype score per gene (e.g. the strength of the phenotype) and search for network regions enriched for high-scoring genes [16], [17], [21], [22]. The second class works on multi-dimensional phenotypic profiles, and assesses the similarity of them between genes being close in the network [7], [23], [24]. In these cases, multiple measurements are available to describe the loss of function phenotype, such as the number of objects, their average size, the average intensity of a marker protein and so on. We could not find a method integrating *multiple* phenotype vectors per gene with interaction data.

Thus, there is a need for new computational methods allowing for the integration of multi-parametric phenotypic data with molecular interaction information.

Here, we present a computational framework called IMPACT (Integrative Multi Profile Analysis of Cellular Traits) that integrates high-dimensional, quantitative phenotypic profiles with independent data like protein interactions. We devised two algorithms operating on two different types of prior information: sets of related genes (IMPACT-sets) and network information (IMPACT-modules). This framework offers several advantages: first, it can handle multiple phenotypic profiles per gene; second it avoids *a priori* definition of ‘hit genes’ based on score thresholding; third, it allows to rescue genes that do not have a significant phenotype based on the RNAi data alone, but show a behavior consistent with their interacting partners. Further, it can cope with many potential biases, e.g. caused by the different frequency of phenotype patterns in the screen, by the structure of the network, or due to variable numbers of knock-down experiments per gene. We validated both methods using a multi-parametric genome-wide RNAi screen on endocytosis [3] leading to new insights into the underlying molecular pathways.

Implementations of IMPACT-sets and IMPACT-modules as well as the data used in this publication are freely available at http://cellnet.cecad.uni-koeln.de/impact.html.

The source code is available at https://github.com/SimeoneMarsico/IMPACT.

## Results

### Overview of the computational methods

**Figure 1:**
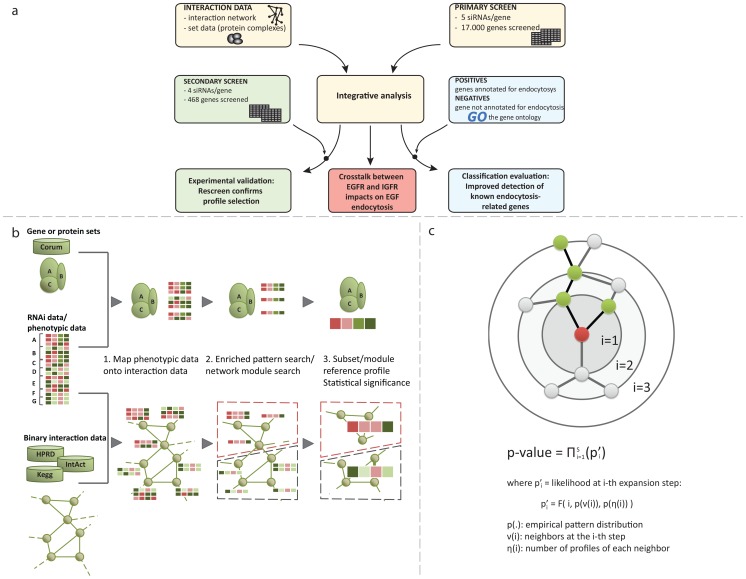
Pipeline and schematic illustration of the integrative analysis. (**a**) Overview of the analysis. (**b**) Schematic of the two integrative methods. They combine phenotypic information from RNAi screening data (heatmaps) with either known protein complexes (top) or binary interaction networks (bottom). Each gene (A through G) has been assayed with several different siRNAs, i.e., several profiles are obtained. The heatmap thus shows the different profiles per gene using a color code (deviation from average); each column represents a phenotype parameter (such as number or size of endosomes). In the case of sets (top) our method assesses all profiles associated with all genes in the set and identifies an over-represented profile. In the case of network data (bottom) the method searches for network modules (sub-networks) enriched for a common profile. In both cases the algorithm determines a ‘references’ profile representing the common profile of the set or network module. Note that in the network case we also consider anti-correlated profiles, as genes might have opposing effects on the readout. For the set-based analysis, significance is determined through appropriate randomizations that take into account the number of genes and profiles in each complex (**c**). For the network-based analysis, the significance of each module is estimated by a semi-analytical approach that takes into account the composition of the module at any expansion step and the probability of observing specific patterns in the dataset (see Methods for details).

We designed a general framework that combines data from quantitative multi-parametric measurements with protein interaction information (Figure 1). We refer to the set of parameters measured after each knock-down experiment as ‘phenotypic profile’. Given several profiles from different si-/esi-RNAs targeting the same gene, our aim was to identify the most likely ‘authentic’ profile, i.e. selecting those profiles that are least affected by noise and OTEs. IMPACT exploits that profiles being similar across interacting genes/proteins are more likely true (Figure S1). For this filtering process, we developed two methods using two types of gene-gene relationships: sets of genes and binary network information (Figure 1 b).

#### Gene set-based analysis (IMPACT-sets)

The first method utilizes pre-defined sets of genes with common properties (e.g. genes encoding components of a multi-protein complex or common pathway). The aim is to identify an enriched phenotype that is shared by a maximum number of genes/proteins in the set. We achieve this goal using an approach that is similar to common cluster analysis, but constrained on the members of a given set: the algorithm identifies the largest group (cluster) of profiles spanning a maximum number of genes in the set (see Methods for details). The advantage compared to standard cluster analysis is that the use of prior information (set membership) forces the method to select common profiles for genes that are known to operate together. Once protein complexes with coherent phenotypic profiles are identified, they are scored with respect to how likely such consistency might occur by chance (p-values). Empirical p-values are computed by permuting the gene labels across complexes many (e.g. 5,000) times (Figure S16). Importantly, this permutation keeps together sets of profiles that were obtained for the same gene. For this set-based analysis, we used 1930 complexes with phenotype information from a database of more than 2000 experimentally verified mammalian protein complexes [25].

#### Network-based analysis (IMPACT-modules)

The second method combines the RNAi screening data with binary interaction data, such as physical protein binding data. In this case the aim is to screen the network for sub-networks (modules) enriched for a common phenotypic signature. The components of such network modules are assumed to be involved in the same or related pathways or biological processes. This approach is based on a greedy search for sub-networks consisting of genes with at least *k* profiles that are correlated with a given ‘seed profile’. This search iteratively expands a network module around a seed gene using similarity with the seed profile as a selection criterion. See Methods for details on how to select the seed profiles. The probability (p-value) of obtaining the observed sub-network by chance is subsequently computed by taking into account (1) the number of genes in the sub-network, (2) the number of profiles (knock-downs) per gene, (3) the number of neighbours per gene in the expanding module, and (4) the frequency of the seed profile in the entire network (Figure 1c). This algorithm computes the probability of finding the observed number of correlated neighbor genes at each expansion step by accounting for the exact neighborhood at that iteration (i.e. the number of interacting genes and the number of profiles per gene; see Methods for details). This strategy accounts for the potential biases introduced by different numbers of profiles per gene. Further, we noticed that some phenotypic patterns are shared by more genes than others, which affects the probability of observing such profile in a random set of genes (see Methods). Hence, IMPACT also corrects for that potential bias. For the network-based analysis we combined binary protein-protein interaction data from three databases reporting experimentally verified interactions: HPRD [26], IntAct [27], and KEGG [28]. The final network contained 9,642 genes and 49,827 non-redundant interactions.

Both approaches require the definition of thresholds (e.g. a similarity threshold for phenotypic profiles). However, it is not necessary to decide on a list of ‘hit genes’ *before* integration with the interaction data. Instead, the algorithms consider all genes and all of their profiles, provided the genes are part of the network (or sets, respectively). Our framework can also deal with multiple knock-down experiments per gene without having to combine (‘average’) the profiles before the integration with external data.

#### Selected profiles and reference profile

A major goal of IMPACT is to select those phenotypic profiles that are least affected by noise and OTEs by using external, independent information. These ‘selected profiles’ are subsequently used to define a representative profile called ‘reference profile’, computed as the median of all selected profiles belonging to a given module or set (Figure S1b; see Methods). The reference is not used during the module/set search procedure: it is an output that provides summarized information of the phenotypic effect for each module or set identified.

In the case of networks, selected profiles include both positively and negatively correlated phenotypes within the same module. This approach accounts for the possibility of inhibitory (negative) interactions between members of the same pathway. However, in the case of protein complexes we require that all selected profiles have a positive correlation with each other. These decisions might have to be adapted when IMPACT is applied to other data. Because of the consensus among interacting partners, both, the selected profiles and the reference profiles are thought to better reflect the ‘true’ phenotype of the genes. We operationally define the ‘true phenotype’ of a gene as the phenotypic profile that would be obtained if the assay was perfect (i.e., no noise, identical knock-down efficiency across all genes, no OTEs, etc.). Of course, we do not know the ‘true’ phenotype of any single gene. However, below we provide multiple lines of evidence suggesting that the selected profiles are indeed closer to that ideal scenario than the discarded profiles.

An important assumption underlying our analysis is that physically interacting genes are more likely to show a similar (i.e. positively or negatively correlated) phenotype. Indeed, genes that are linked in our network have significantly more similar phenotypic profiles than random pairs of genes (p-value of 7×10^−11^ and 2×10^−7^ with the Kolmogorov-Smirnov test and Mann-Whitney U test, respectively).

### Classification performance on known endocytosis-related genes

We applied our methods to an image-based, genome-wide RNAi screen assessing the role of genes in transferrin (TF) and epidermal growth factor (EGF) endocytosis in human HeLa cells [3] (Figure 1a and Input Data in Methods). Forty quantitative parameters describing various aspects of cargo uptake and propagation along the endocytic pathway, such as endosome number, size and intracellular distribution, were extracted by image analysis [3], [29] (Table S1). On average about 7 si-/esi-RNA per gene were screened. Ideally, one would expect a high correlation between the phenotypic profiles of different siRNAs targeting the same gene. However, those profiles were often not significantly correlated (Figures S1 & S2). Such inconsistency is neither caused by technical or biological variation in the screen, nor by different silencing potency of the siRNAs [3], but mainly due to siRNA-specific OTEs [3], [8], [30], [31].

In order to systematically and quantitatively assess the performance of recovering genes involved in endocytosis, we compiled a set of known endocytosis-related genes as positive controls (Figure 1a). This selection is based on relevant Gene Ontology (GO) terms and exclusively using experimentally inferred gene annotations (in total 387 genes annotated for the terms reported in Table S2). The negative control set (21,585 genes) was assembled considering genes that are annotated with functions other than endocytosis (i.e. genes without any annotation were excluded from this analysis).

**Figure 2:**
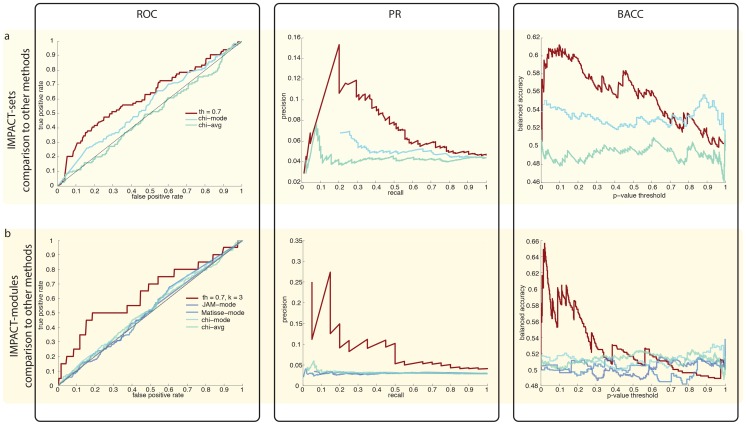
ROC, Precision Recall and Balanced Accuracy analysis. Validation of the combined results obtained with the set- and network-based analyses using known endocytosis-related genes as a positive reference set. The first column shows Receiver Operator Characteristic (ROC) curves, the second column displays Precision-Recall curves and the third column contains Balanced Accuracy curves (BACC). Row (**a**): comparison IMPACT-sets (*T* = 0.7) to a method based on the Chi-square statistics scoring genes exclusively using RNAi data (‘Chi-square mode’, ‘Chi-square avg’). Row (**b**): comparison of the IMPACT-modules (*T* = 0.7, *k* = 3) to two published methods (JActiveModules and MATISSE) and to the Chi-square method (‘Chi-square mode’, ‘Chi-square avg’).

We ranked the genes based on the p-values of the protein complexes or network modules they belong to, and tested whether known endocytosis related genes (i.e. genes from the positive set) rank higher than the negative set genes. We used Receiver Operator Characteristic (ROC), precision-recall (PR) curves, and balanced accuracy (BACC) [32] curves (Figure 2) for visualizing to what extent IMPACT distinguishes known endocytosis-related genes from the negative set. We also computed the Area Under the ROC Curve (AUC, [33]) to quantitatively compare the overall performances of different search parameters and across different methods.

#### Comparison of algorithm search parameters

Since our integrative approach requires the definition of a ‘similarity threshold’ *T* (based on the Pearson correlation coefficient *R*) for calling two profiles ‘similar’, we tested several values of *T* for the set-based (Figure S6 a) and network-based analysis (Figure S7 c). We obtained the best performance for both the set- and network-based approaches using cutoff values of *T* = 0.7 (AUC-IMPACT-sets *T = 0.7*: 0.619; AUC-IMPACT-modules *T = 0.7*: 0.648). In case of the network-based analysis one has also to choose the minimum number (*k*) of profiles that have to be similar per gene to include a gene in the current network module. We obtained the best result with *k = 3* and *k = 2* (AUCs 0.648 and 0.553 respectively (Figure S7 a). All the following tests have been carried out with *T* = 0.7 and *k* = 3 for modules (AUC = 0.648) and *T* = 0.7 for sets (AUC = 0.619), unless explicitly stated otherwise. Since IMPACT-sets requires positive values of correlation above the threshold *T* for the searching, we tested the effect of allowing also negatively correlated profiles to be included, i.e. similarity is the absolute value of correlation as in IMPACT-modules (see Methods). Allowing for anti-correlation in IMPACT-sets reduced the AUC from 0.619 to 0.567, suggesting that by-and-large phenotypic profiles of genes whose products participate in the same complex are positively correlated. Additionally, enforcing positive correlation in IMPACT-modules slightly decreased performances (AUCs from 0.648 to 0.627 for *T* = 0.7, *k* = 3 and from 0.553 to 0.539 for *T* = 0.7, *k* = 2; Table S13).

#### Effects of seed selection, network coverage, and parameter subsets on module search

We tested how seed selection affects final estimates of significance: relaxing the seed selection threshold *T_s* (see Methods) from 0.8 to 0.5, i.e. allowing for the inclusion of less correlated phenotypic patterns as starting point for the search, reduced the algorithm's performance (AUC for *T_s* = 0.8: 0.648; AUC for *T_s* = 0.5: 0.571). Reshuffling the selected seed profiles to random positions within the network lowered the performance (for *T* = 0.7, *k* = 3: AUC = 0.59 and 65% less modules; for *T* = 0.7, *k* = 2: AUC = 0.507 and 50% less modules than the respective real cases), suggesting that seed profiles are indeed specific for their network context (Table S13).

We investigated the effect of network coverage and parameter subsetting on the classification outcome, to test respectively the effect of prior information or phenotypic readout on module identification. For evaluating network coverage sensitivity, we tested IMPACT-modules on modified networks obtained by random removal of 33% and 50% of the interactions. A sparser network leads to lower classification performances (AUC = 0.62±0.076 and 0.55±0.085, respectively; *n* = 7 random tests each), but the performance is still better than the random case with AUC = 0.5 (p-values of 0.05 and 0.28, respectively). Thus, even an incomplete network improves the detection of correct phenotypes.

Next, we evaluated the importance of using the complete 40-parameter profiles by comparing the results obtained when using only a sub-set of the parameters. Here, we grouped the parameters into 7 groups (G1 to G7) according to their biological similarity (Table S1) and compared the results after systematic removal of each group of parameters. In general, we noticed that removal of parameters reduces the performance to different extent depending on the parameters removed (AUCs in the range [0.5034, 0.63] versus 0.648 of the full parameter set; Table S7). The fact that the performance is reduced in all 7 cases suggests that all parameters are informative.

#### Effects of network permutation and phenotypic noise on module search

We also explored the effect of randomly permuting: 1) network topology (i.e. shuffling edges); 2) gene assignment (i.e. shuffling genes with all their profiles on the same network structure); 3) profile assignment (i.e. shuffling profiles across genes on the same network structure) and 4) network and profile assignment (i.e. shuffling profiles and edges). In all cases the performance went down, with AUCs of 0.529±0.070 (p = 0.34), 0.515±0.087 (p = 0.43), 0.510±0.081 (p = 0.45) and 0.433±0.067 (p = 0.78) versus 0.648 (p = 0.01) of the real case (values are averaged for 3 independent randomizations; method parameters always set to *T = 0.7* and *k = 3*).

We further investigated the role of overall noise in the screening data by adding various levels of noise to the phenotypic profiles. To do this, we added to each parameter of the original phenotypic data normally distributed values *N(μ, σ)* with mean *μ = 0* and standard deviation *σ* = 0.05, 0.1 and 0.2 (i.e., we perturbed each parameter with random noise of 5, 10 and 20% of the original z-normalized data variance). After repeating IMPACT-modules (*T = 0.7*, *k = 3*) on *n = 4* independent random cases, we obtained average AUCs of 0.542±0.073, 0.561±0.077 and 0.556±0.073, respectively, worse when compared to 0.648 of the real case, but still better than random (p-values of 0.28, 0.21 and 0.22). Also, the number of identified modules decreased progressively to 63%, 57% and 37% with increasing noise added, i.e. also the sensitivity is affected. The fact that the AUCs stay above the background level underlines the robustness of IMPACT-modules.

#### Comparison to screen analysis

Using external information for the profile selection clearly improved the recovery of known endocytosis genes compared to not using such information. This is shown by comparing our results (IMPACT-sets and IMPACT-modules) to a method based on the phenotypic strength as measured by the Chi-square test, which was used in the initial publication of the RNAi screen [3] (Figure 2). Specifically, we used two versions of the Chi-square test: the first using the average profiles and the second the mode profiles, the latter being used in the previously published analysis [3]. Our results show improved selection of endocytic genes (higher AUCs) for both, protein complexes (Figure 2a, Table S9) and network modules (Figure 2b, Table S8). We also compared the true and false positive rates of our two methods with the values from the hit list of the original publication, where a combined approach assessing phenotypic strength (Chi-square on mode profile) and phenotypic specificity (Phenoscore on mean-shift clustering) was used to define scoring genes. Our results show better performances (Figure S9).

Further, we compared the counts of endocytosis genes (based on GO annotation) selected as significant by IMPACT (p-value< = 0.1) to the count in the hit list based on the Chi-square of the mode profile. To perform a balanced comparison, we selected the 2,720 significant genes from IMPACT-sets and IMPACT-modules (Table S5) and the top 2,720 genes from the sorted Chi-square list (Figure S10). The endocytic genes recovered specifically by IMPACT (79) are more than the ones missed (36, of which only 26 map on the interaction data), showing that our method has a better sensitivity/specificity trade-off, as also highlighted by the AUC analysis.

An important feature of IMPACT is that alternative phenotypic profiles per gene do not have to be collapsed to a single profile prior to the analysis. In order to investigate the relevance of this feature, we applied the IMPACT to single profiles per gene computed either (i) as the average, *single-avg*, or (ii) as the multi-parametric mode, *single-mode* (representing the most probable profile [3]). In both cases, all performance measures (ROC, PR and BACC curves) went down for the module-based approach (Figures S7b) and the AUCs decreased to 0.528 and 0.496 for *single-mode* and *single-avg* (Table S4, right). For the set-based approach, we observed AUCs of 0.636 and 0.571 for *single-mode* and *single-avg* (Figure S6b; Table S4, left). Thus, using *single-mode* for IMPACT-sets slightly improved the AUC compared to the standard analysis (AUC = 0.619). However, the number of genes in significant complexes decreased to 16% (for *single-mode*) and 18% (for *single-avg*) of the total number of significant genes detected when considering all profiles prior to averaging. This underlines the importance of considering all available profiles separately for maximizing accuracy and sensitivity.

#### Comparison to other methods

We compared our network-based approach to two published network-based methods, JActiveModules [21] and MATISSE [23]. JActiveModules is a representative for the class of methods assuming one score per gene and then searching for sub-networks that are enriched for high-scoring genes. MATISSE represents methods operating on single multi-dimensional profiles per gene. None of the methods can cope with multiple profiles per gene. We used each gene's mode-profile as input for MATISSE and the p-value of the Chi-square statistic calculated on the mode profile as input for JActiveModules. Our network analysis performed better than both, JActiveModules and MATISSE (AUCs of 0.507 and 0.518; Figure 2b and Table S8). We previously performed parameter tuning to choose the best performing parameters (*T = 0.7*, *k = 3*) by analyzing the classification of endocytosis GO terms. In order to ensure a fair comparison we also tested the method performances on an independent list, selected according to different criteria. We used the merged list of Rab5 effectors [34] and of proteins containing domains that are known to be related to endocytosis (PX, FYVE, BAR, TBD and VPS9, [3]), comprising of 306 members, of which 213 are present in the interaction network (the overlap with the endocytosis GO annotation gene list was just 62 genes). IMPACT-modules (both for *T* = 0.7, *k* = 2 and *T* = 0.7, *k* = 3) out-performed the other approaches on this new list as well as on the union of the two, the GO terms list and the RAB5 effectors and proteins with endocytic domains list (Table S10 and Figure S15). Also IMPACT-sets performed better on this new list of positives (AUC = 0.624) when compared to Chi-square on mode and average profiles (AUCs of 0.571 and 0.518 respectively).

The degree of a gene in the network (the number of its neighbors) is an indicator of pleiotropy. Thus, genes with a high degree are more likely to be involved in many cellular functions, potentially leading to inflated performance estimates when using networks to predict gene function [35]. We therefore tested if the improved enrichment of known endocytosis genes among high scoring network modules might be an artifact of the degree distribution, but this was not the case (Figure S8, Table S8).

In summary, these analyses demonstrate that including protein interaction information improves the filtering of relevant phenotypes from high-dimensional functional screens and it underlines the importance to maximally exploit the information contained in the several, multi-dimensional profiles obtained per gene.

Most of the above analysis required at least three profiles per gene to be similar with interacting genes for inclusion in a network module (*k* = 3). Such stringent threshold led to very good recovery of known endocytosis genes. In order to improve the potential for new discoveries we subsequently lowered the required number of consistent profiles from 3 to 2. This value still yields a better performance compared to other methods (Table S8), while increasing the number of newly predicted genes in network modules 10-fold.

### Rescreen confirms profile selection

In order to also experimentally validate that our approach improves the phenotype selection, we rescreened 468 genes from the most significant protein complexes and network modules (Table S5) using an improved set of 4 siRNAs per gene (see Methods). The siRNAs used for this rescreen represented a new, independent set of reagents from a different provider, produced with newer technology, which improves the knock-down efficiency, induces less toxicity, and lowers off-target effects [36]. In order to independently confirm the improved quality of the new siRNAs we validated that both, individual parameters as well as phenotypic profiles are more reproducible using the new set of siRNAs (Figures S3 & S4). Importantly, profiles of different siRNAs targeting the same gene are more similar in the rescreen compared to the primary screen. Therefore, the new profiles are expected to be closer to the true phenotype.

**Figure 3:**
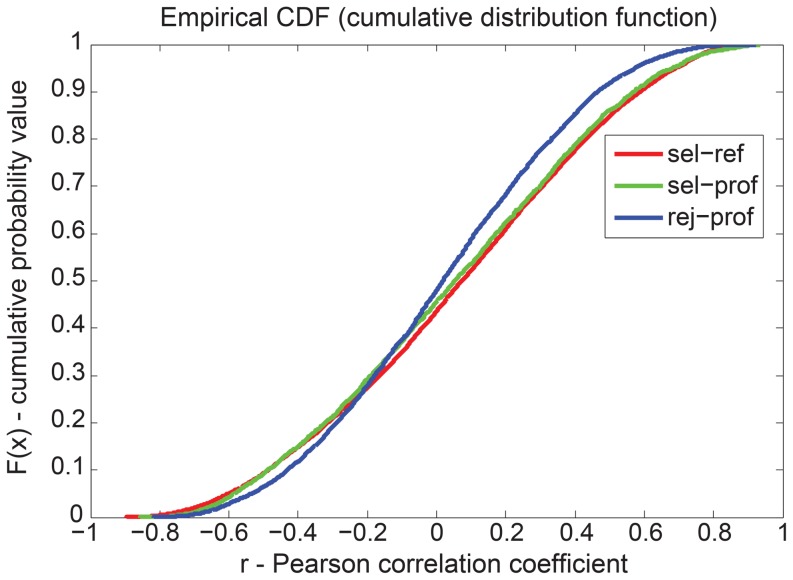
Independent experimental validation of the selected profiles. Selected profiles (‘sel-prof’, green), rejected profiles (‘rej-prof’, blue) and reference profiles obtained by averaging selected profiles (‘sel-ref’, red) are compared to respective profiles from a rescreen using improved siRNA oligonucleotides. Cumulative distributions of the Pearson correlation coefficients (*R*) between profiles obtained for the same gene are shown (468 genes were rescreened). Selected profiles are significantly better correlated with rescreen profiles than rejected profiles (p = 8.51×10^−5^ MW test; p = 8.94×10^−13^ KS test); reference profiles are even more strongly correlated with the rescreen profiles (p = 1.48×10^−18^ MW test; p = 1.1×10^−32^ KS test). Note that the correlation of rejected profiles (‘exc’) is not better than random. MW test = Mann–Whitney U test, KS test = Kolmogorov-Smirnov test.

The profiles selected by IMPACT are thought to be closer to the true phenotype of the genes than the rejected ones and, thus, should also be more similar to the rescreen data. Indeed, we observed that the pairwise correlation of the selected profiles to the new profiles is significantly higher than the correlation between rejected and new profiles (Figure 3). Furthermore, the reference profiles (i.e., the median of selected profiles per set or network module) are even more similar to the rescreen data than the selected profiles (Figure 3). Although being significant, the improvement is not dramatic: this is partly due to the fact that the phenotypic data for the new set of oligonucleotides are better but still noisy (Figure S3 and S4). To confirm this notion, we selected a few strong examples where the set of new oligo profiles show high intra-similarity within the rescreen (suggesting low noise). The similarity of the profiles selected by IMPACT is much higher to this new set than to the old ones for the same gene. Among those, we had some genes important for endocytosis (PDPK1, Furin, MLC1) and for signaling (ERBB2, IGF1R) (Figure S19).

These data demonstrate that our analysis successfully selected profiles that are more reproducible in the rescreen and likely better reflect the true function of the genes. Furthermore, the reference profiles, representing the consensus phenotype of a protein complex or network module, were even less affected by noise.

### Phenotyping endocytosis core machineries

**Figure 4:**
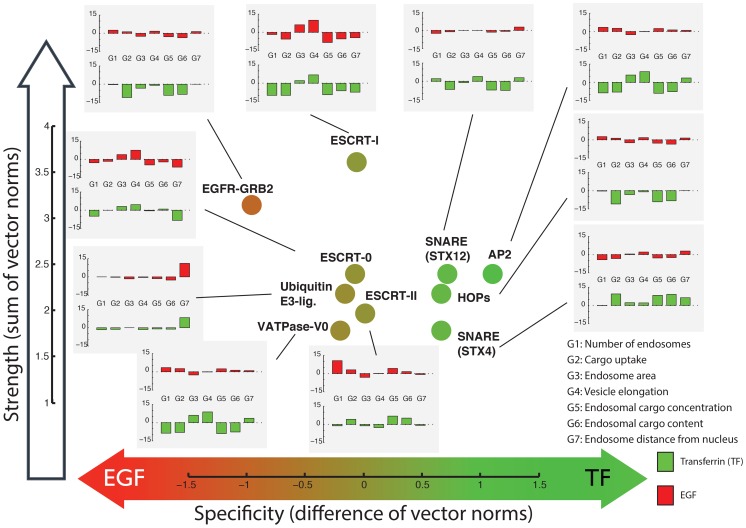
Selected protein complexes with enriched phenotypes. Each complex is represented by its reference profile, which is computed as the most consistent signature across all members of the complex. The analysis is based on 19 parameters for each channel (EGF and transferrin, TF) and two co-localization parameters (TF with EGF and EGF with TF). The x-axis shows the difference between the Euclidean (or L2) norm of the transferrin and the EGF parameters 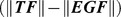 the y-axis represents the strength of the phenotype computed as the sum of the norm of both the transferrin and the EGF signals 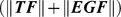. Each protein complex is shown as a circle, its color reflecting the primary direction of the phenotype (value on x-axis). Inset bar-plots show summary versions of the reference phenotypic profiles by projecting them from 40 to 14 dimensions (red: response on EGF channel, green: response on transferrin channel). See Table S1 for details on the parameters. More details about complex membership are shown in Supplementary Figure S13.

**Figure 5:**
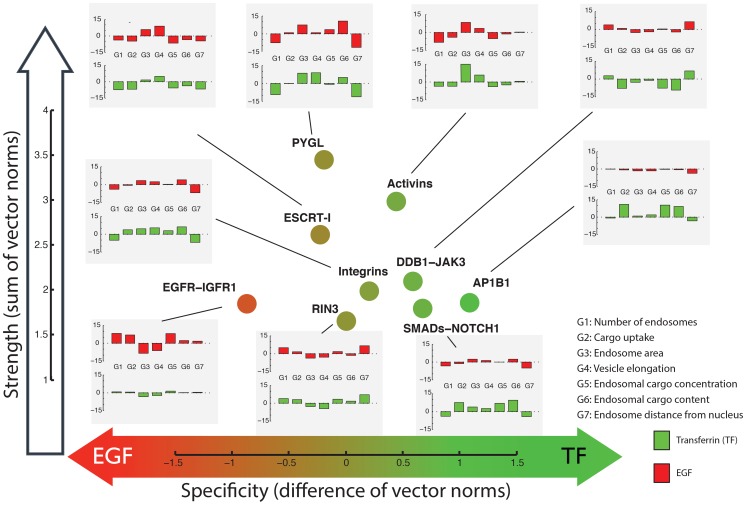
Selected network modules with enriched phenotypes. See Figure 4 legend for more details. The topology of the extracted sub-networks is shown in Figure S14. Note that genes in the same module (as opposed to protein complexes) can have anti-correlated profiles, but selected profiles of the same gene must be positively correlated. The profiles shown represent the direction taken by the majority of the genes in the module. E.g., the phenotype of EGFR is anti-correlated to the reference profile shown for the EGFR-IGFR module.

In order to visualize the phenotypes of the analyzed complexes and network modules we created a ‘phenotype map’ representing the strength and specificity of the phenotype for transferrin or EGF (Figures 4 & 5). This visualization groups phenotypically related complexes and network modules and it also shows simplified representations of the profiles, thus, facilitating the interpretation of the findings. Even though the analysis above already showed that our method improved phenotype selection, we also verified the validity of our results by focusing on proteins and protein complexes with known functions related to endocytosis. Our analysis rescued several genes that did not score in the initial analysis [3], like RAB4A, SARA (ZFYVE9), APPL1, RAB11FIP1, VPS28, VAMP8, VIT1A, STX2 and SNX1 (see Table S12 for full list of 91 endocytic genes selected by IMPACT and missed in the previous analysis). Genes selected by IMPACT were enriched also for other endocytosis-related functional terms from the KEGG and GO annotations (DAVID analysis, [37], [38]), such as endocytosis (p = 1.9e-3, modified Fisher's Exact Test), phosphatidylinositol signaling system (p = 1.3e-14) and inositol phosphate metabolism (p = 1.4e-8) for KEGG; membrane enclosed lumen (p = 1.7e-26) and membrane bounded vesicle (p = 1.5e-5) for cellular compartment (GO CC); membrane fusion (p = 1.8e-3), invagination (p = 6.5e-2) and docking (p = 9e-2) for GO biological processes (GO BP). Moreover, our method selected expected phenotypes for several known cellular machineries. The AP2 complex, for instance, is known to be primarily involved in transferrin endocytosis [39]. Even though phenotypic profiles of individual AP2 subunits were ambiguous, our method correctly identified the transferrin-specific phenotype as being enriched in this complex (Figure 4 and S1).

The integrative analysis allowed us to reveal subtle phenotypic differences between closely related machineries. Two examples are the families of SNARE and ESCRT complexes (Figure 4 and S13). The reference profiles extracted for those complexes through our method again suggest possible insights into molecular mechanisms, therefore posing the basis for focused experimental testing (Text S1).

### Impact of signaling pathways on endocytosis

Internalization and trafficking of signaling molecules such as membrane receptors is crucial for many signaling pathways. Whereas the importance of endocytosis for signaling is well established, much less is known about how signaling pathways control endocytosis [40], [41]. The network analysis allowed us to gain insights into this process by identifying several signaling pathways over-represented in statistically significant network modules, such as the ErbB and Insulin signaling pathway, the focal adhesion and actin pathway and pathways involved in diseases, particularly cancer (Table S11). Also, our analysis further elucidated how the position of a protein in a pathway relates to its phenotype.

For example, we detected two transforming growth factor beta (TGF-beta) related network modules with distinct phenotypic profiles (“Activins” and “SMADs-Notch”, Figure 5). Consistent with the fact that Activins and SMADs act in the same pathway, our algorithm assigned related phenotypes to them, both showing a reduction of transferrin and EGF uptake, as already reported [3] with a stronger impact on transferrin than EGF (Figure 5). However, our analysis also uncovered significant differences between these two parts of the TGF-beta pathway. The first module contains several Activin receptors (ACVR1B, ACVR2B, ACVR2A, ACVR1 and AXVRL1) that are known to modulate and transform signals for the TGF-beta superfamily of ligands. The second module links the TGF-beta and Notch pathways [42]. This SMADs-Notch module has a core consisting of SMAD2, SMAD3 and NOTCH1, which in turn are associated with several transcriptional regulators (Figure 5). SMAD3 and NOTCH1 were missed in the initial screen hit list and have been rescued by the integrative analysis. Knock-down of genes in both, the Activins and SMADs-Notch sub-networks, significantly reduced the number of endosomes (G1), underlining the importance of these pathways for endocytosis. However, knock-down of the Activin module reduces cargo uptake (G2), whereas knock-down of the SMADs-Notch module increases cargo uptake for transferrin endosomes.

The difference between the Activins and SMADs-Notch modules underlines that upstream and downstream components of the same signaling pathway (i.e. the TGF-β pathway in this case) can have different effects on endocytosis. Thus, the position of proteins in the pathway seems to critically affect the impact on the assay's readout.

#### Crosstalk between EGFR and IGFR impacts on EGF endocytosis

Another network module related to cell signaling contains the EGF receptor (EGFR) together with huntingtin (HTT), catenin delta 1 (CTNND1) and the IGF-1 receptor (IGFR, Figure 5). This module was of particular interest because of the importance of IGF-EGF crosstalk in signaling and cancer [43], [44]. Our analysis suggested a specific effect of this interaction on EGF endocytosis (and not transferrin). Moreover, the Insulin signaling pathway was one of the most enriched among the ones identified by IMPACT-modules. IGF1R was specifically rescued by our analysis as a weak but specific phenotype and the results were confirmed by our rescreen. Taken together all these facts lead us to investigate the EGFR-IGFR interaction more in detail.

All proteins in this module, except for EGFR, share a phenotypic profile in which EGF endocytosis is up-regulated. Whereas knock-down of EGFR causes a decrease in EGF endocytosis, as expected, silencing of the other genes in the module leads to an anti-correlated profile, consisting of increase in the number of EGF-positive endosomes, endosomal EGF concentration and distance of EGF endosomes from the nucleus. In contrast, the transferrin related parameters did not show relevant changes. We were particularly interested in the crosstalk between IGFR and EGFR, because the constitutive activity of these signaling pathways is characteristic of many tumors and chemotherapy resistance [43]–[48]. Our analysis suggests that IGFR exerts a specific regulation on EGFR trafficking, an observation that has not been reported so far. We reasoned that this crosstalk might be mediated either by a direct interaction between the two receptors [44] or by a modulation of EGFR trafficking by IGFR signaling.

**Figure 6:**
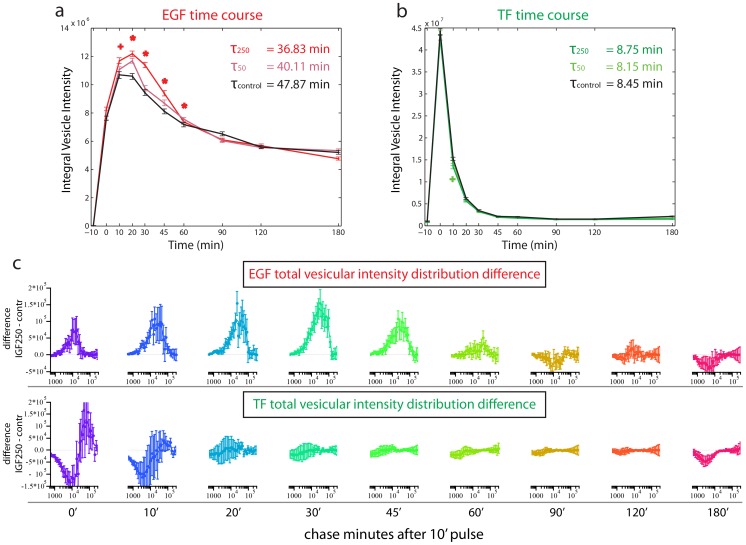
IGF-1 co-stimulation experiments. Pulse-chase experiment of labeled EGF and transferrin in presence of IGF-1. The 10 minutes pulse lasts from time point −10′ to 0′. (**a**) Temporal profile of the total vesicular intensity calculated for the EGF-positive endosomes, with different concentration of IGF-1 (dark red: 250 ng/ml; light red: 50 ng/ml; black: no IGF-1 (0 ng/ml), or control). (**b**) Temporal profile of the total vesicular intensity calculated for the TF-positive endosomes, with different concentration of IGF-1 (dark green: 250 ng/ml; light green: 50 ng/ml; black: no IGF-1 (0 ng/ml), or control). The insets within panels (a) and (b) show the estimated time constants obtained by fitting a decaying exponential function to the last part of each curve, for the corresponding concentrations (see Figure S11 for details). Asterisks (*) and plus sign (+) above individual time points indicate significant deviation from the control assessed by t-test (p≤0.0001 and p≤0.01, respectively). (**c**) Time course analysis for different chasing times: each mini-plot represents the difference of distributions (y-axis) of the total cargo content in endosome sub-populations of different mean content (bins in the x-axis) for EGF-positive endosomes (upper row) or TF-positive endosomes (lower row). All mini-plots show the difference between the distribution of the IGF-1-stimulated case (250 ng/ml, IGF250 in the label) and the control (contr in the label) for the corresponding time point and cargos. See Figure S9 and S10 for more details.

To test this possibility, we measured the effect of IGF-1 stimulation on the cellular uptake kinetics of EGF and transferrin by confocal fluorescence microscopy. For this, HeLa cells were pulsed with EGF and transferrin for 10 minutes (see Methods) and chased for different periods of time, in the absence or presence of 50 and 250 ng/ml IGF-1. We found that IGF-1 significantly accelerated the kinetics of EGF internalization (*integral vesicular intensity*, Figure 6 a), whereas transferrin uptake was unaffected (Figure 6 b). After an initial accumulation phase of approximately 20 minutes, intracellular vesicular EGF decreased monotonously following first-order kinetics, consistent with endosomal degradation [49]. Transferrin total intensity (integral vesicular intensity, Figure 6 b) showed a rapid monotonous decay after pulse, in agreement with its recycling kinetics [50]. The decay of EGF (i.e. degradation) was specifically accelerated in the presence of IGF whereas that of transferrin (i.e. recycling) was unchanged, as quantified by the estimation of the rate constants after fitting a decaying exponential function to the experimental data (Figure S11).

To better visualize the changes induced by IGF-1, we subtracted the total cargo vesicular intensity distributions between the control and stimulated conditions for each time point. This representation (Figure 6 c) emphasizes the changes in cargo content in different sub-populations of endosomes out of the total endosomal network. The results indicate that IGF-1 exerted two main effects. First, it caused the appearance of a higher number of endosomes containing EGF, as shown by the positive values in vesicular intensity differences (Figure 6 c, panel above). Second, stimulation caused a faster endosomal accumulation of EGF (note the shift over time of the peak toward higher vesicular intensity values, Figure 6c, panel above). In contrast, the transferrin was re-distributed between endosomes only at the initial time points (Figure 6 c, panel below), without alterations in the total amount of intracellular transferrin (note that the positive and negative values of vesicular intensity differences neutralize each other, Figure 6 c, panel below).

In conclusion, our analysis was able to uncover a novel aspect of the crosstalk between IGF-1 and EGF, through acceleration of EGF uptake and transport along the degradative pathway. These results demonstrate the ability of IMPACT to reveal novel interactions between functional modules and to assist the molecular interpretation of genetic screens.

### Application of IMPACT to other data sets

We evaluated the general applicability of IMPACT in three different ways: first, we applied IMPACT-modules to the same RNAi screening data, but using a different network as a prior. Second, we ran it on another siRNA screen with autophagy as an endpoint [51] and finally, we used IMPACT to analyze a CRISPR-Cas9 knockout screen in human cells [52] (see respective paragraphs in Text S2).

We run IMPACT-modules for the endocytosis screen on different interaction networks derived from the STRING database [53]. STRING incorporates diverse types of information, such as co-expression, experimentally validated protein binding, and text mining, to predict the functional relationships between genes. Importantly, it can be used to evaluate the importance of these individual feature types for the phenotype prediction. This analysis revealed that the choice of the network strongly affects the quality of the phenotype prediction. Specifically, we noticed that 1) the performance deteriorates when considering co-expression data only (AUC = 0.505); 2) experimentally validated interaction networks yield better classification (AUC = 0.6483 for the HPRD-Intact-KEGG combined network and 0.603 for STRING experimental) than networks allowing also non-experimental interactions such as database and text mining predictions (AUC = 0.553). See paragraph Other sources of prior information, Text S2. Thus, this analysis confirmed that using high-quality, experimentally confirmed protein interaction data maximally reduced noise from the RNAi data. Importantly, both experimental networks (our combined and STRING-experimental) gave results that were better than random.

Next, we run IMPACT-modules on a siRNA autophagy screen in the human HEK293 cell line [51] where 3 replicates of the entire screen were acquired and 3 different image-based parameters were measured. We analyzed the recovery of the known autophagy genes reported in the human autophagy database (www.autophagy.lu, [54]). The original screen analysis identified 25 known genes (out of the 175 autophagy genes screened) among the 1'000 reported hits (enrichment p-value = 0.04); IMPACT-modules identified 1'332 significant genes, of which 46 were autophagy annotated (out of the 161 mapping on the network; enrichment p-value = 2e-3). Also, IMPACT performed better than the ranking measure considered in the screen for classifying the known autophagy genes (AUC = 0.563, p = 2e-4 for IMPACT; AUC = 0.4949, p = 0.59 for hit ranking). See paragraph Analysis of the siRNA autophagy screen in Text S2.

Finally, we run IMPACT-modules on a CRISPR-Cas9 knockout screen in the human melanoma cell line A375 [52], where the authors investigated the effect of gene loss upon treatment with vemurafenib, a therapeutic drug inhibitor of BRAF, by measuring cell viability in 4 different conditions (vehicle versus drug, 7 and 14 days). IMPACT identified 1'659 significant genes (p<0.05). Gene enrichment analysis of over-represent GO biological processes and KEGG pathways (DAVID [37], [38]) revealed interesting insights into the mechanism of action of the drug. Pathways involved in cancer and related to BRAF activity, such as “Melanoma”, “MAPK”, “Pathways in cancer” were strongly enriched (fold enrichment of 2.35, 1.84, 2.06; p-value of 3e-7, 8e-11, 2e-20 respectively). Also, biological processes related to phosphorylation, kinase activity, cell migration, cell proliferation and cell death were strongly enriched (p-values ranging from 1e-12 to 1e-6). The screen hit list derived using RIGER [55] identified overall GO and KEGG terms with higher p-values and lower fold enrichment (i.e. less significant), related mainly to “Oxidative phosphorylation” (p = 1e-4), transcription (2e-4) and histone modification (1.2e-3). See paragraph Analysis of the CRISPR-Cas9 knockout screen in Text S2. Thus, we conclude that IMPACT improves the analysis of functional genomics screens beyond RNAi screens.

## Discussion

Multi-parametric phenotyping is becoming increasingly important for understanding basic biological processes and disease mechanisms. Such high-dimensional phenotyping is being conducted in RNAi screens, (conditional) knock-out screens, quantitative trait loci (QTL) studies, chemical genomics and gene editing screens. In this study, we developed a novel approach for analyzing multi-dimensional functional genomics screens in combination with external information in order to facilitate the mechanistic interpretation and to cope with noise in phenotype detection. Our approach provides significant methodological advantages, because it integrates (1) set-based and network-based prior information, (2) high-dimensional (multi-parametric) phenotypic profiles, and (3) multiple profiles per gene. Further, it handles several potential biases, e.g. due to the network topology (number of neighbors) or the frequency of phenotypic profiles. This data integration of course critically relies on existing interaction information, which is still sparse and noisy. However, our study demonstrates that the impact of noise and OTEs could be drastically reduced for those genes that are part of a network. A broad range of novel insights into the function of both known and new cellular machineries could be gained, only some of which could be addressed in this study. The use of two methods obviously increased the scope of prior information that could be used for the analysis (protein complexes and protein-interaction networks).

### Alternative approaches

We chose to use only protein interaction data to support mechanistic interpretation of the phenotypes in terms of molecular machineries. Co-expression and co-functionality data have a broader coverage of the genome, however they do not necessarily imply molecular interactions and would therefore not satisfy the purpose.

Instead of using IMPACT-sets, one may also transform protein complexes into interaction networks by considering all pairwise interactions instead of protein sets. In this case, one could use IMPACT-modules to perform the analysis. We tested this possibility (IMPACT-modules on sets) and calculated the classification performance (AUC) in identifying known endocytic genes. Running IMPACT-modules on sets still performs better than the other approaches considered in this study, but its performance is worse compared to IMPACT-sets (Table S6). Two reasons may explain this phenomenon: first, the statistical analysis of IMPACT-sets may be better suited for the analysis of sets. Second, the conversion of sets into networks is questionable. Thus, considering set information, when available, rather than splitting it into binary interactions can be advantageous.

The complexity of our approach arises in part from the fact that we considered all phenotypic profiles separately and selected the profiles with an enriched pattern among other genes in the set or interacting genes in the network. A simpler approach would have been to combine all profiles of a gene first (e.g. averaging) and then assessing the consistency in the networks. However, this method would not take into account that different siRNAs often produce very different profiles due to their heterogeneous off-target signatures. In fact, sometimes less than half of the siRNAs yield profiles resembling the correct phenotype (Figure S18) and averaging would result in profiles that are more strongly affected by OTEs and noise. We indeed observed that applying IMPACT after averaging either decreases performances (Figures S6b and S7b; Table S4) or reduces the number of selected genes (Table S5), with a stronger effect on network module identification.

### Method assessment

The classification analysis showed improvements relative to other approaches, both considering (JAM, MATISSE) and ignoring (Chi-square) prior information. However, we were hoping for an even better performance: using IMPACT the AUCs never exceeded 0.65 for endocytosis GO terms and 0.67 for the Rab5 effectors and proteins with endocytic domains. Our analysis showed that noise in both, the RNAi screening data and the network can significantly compromise the performance. However, using a network-prior improves the signal-to-noise ratio, which is even more important when data is noisy. Also, reducing network coverage led to lower performances, suggesting that IMPACT or related methods relying on prior information can further improve as new protein-protein interactions will be discovered. Importantly, whichever method is used, the quality of the results is always limited by the quality of the input data. Thus, all method assessment should be regarded as relative comparisons of alternative approaches.

One concern when comparing methods relying on prior information (such as IMPACT, JAM, Matisse) to methods relying on phenotypic data only is that the performances of these methods may be inflated by the fact that interacting genes tend to share the same annotation used for evaluation, which may result in circular reasoning. This problem cannot be completely solved since equally annotated, connected genes could be at the core of molecular machineries, which makes it difficult to distinguish a circularity bias from the reality of the underlying biology. We have tried to address this problem in two ways: first, we evaluated the enrichment of not just any GO term among top-scoring genes, but terms specific for endocytosis. A network-based method running on random data may still identify sub-networks or protein complexes that are enriched for certain functions, but not necessarily for the functions relevant for the screen. Second, we have performed extensive new experimental validation, which is independent of any reported annotation in public databases.

Application of our two methods to an endocytosis RNAi screen led to improved recovery of known endocytosis-related genes compared to the analysis of the primary screen data alone. Further, based on several performance measures, our network-based method performs better than published methods that are either mono-parametric or do not assume multiple profiles per gene. Importantly, using only one (averaged) profile per gene also reduces the performance of our method, which demonstrates that considering multiple profiles is crucial. The importance to assess sets and modules based on individual-siRNA profiles was also confirmed by the rescreen, which showed that the selected profiles were significantly better correlated with the new independent data than the rejected profiles. Moreover, the partial coverage that prior information based approaches can offer (in our case, 9,715 out of the 17,730 genes in the screen were mapped on the interaction network) suggests as a sensible solution the use of IMPACT as a complement rather than an alternative to other methods relying only on phenotypic information, to combine the strengths of both, i.e. identifying protein machineries and elucidating their function with IMPACT, while still assessing the effect of genes without interaction information.

The computation of the significance of network modules controls for potential biases, such as the topology of the sub-networks, that were ignored in previous work [16], [21], [23], [56]. To address this, our approach takes into consideration the number of neighbors (degree) and the number of profiles (siRNAs) of each gene, as well as the frequency of the enriched phenotypic profile across all genes in the network. Not considering these factors may lead to inflated or deflated significance estimates. Note that global permutation testing [16], [56] would neither account for the specificities of a given phenotypic profile nor for the local topology of the network. Whereas our network search is based on a heuristic greedy search, other methods [57], [58] provide exact solutions using constraint programming for module search. However, these approaches can only deal with a single score per gene; further development will be necessary to devise similar strategies for multi-parametric measurements and multiple profiles per gene.

### Pathway analysis

Recent work already suggested feedback regulation of signaling pathways onto endocytosis [40], [59] [3]. The findings in this study (Table S11) underline the tight bonds between the endocytic machinery and signaling networks. Additionally, our network analysis revealed that different modules within the same signaling pathway can exert diverse effects on trafficking (e.g. Activins- and SMADs-containing modules within the TGF-beta pathway), whereas components of different signaling pathways (e.g. TGF-beta and Notch) can have similar effects on endocytosis. Thus, there is no trivial relationship between a gene's membership in a signaling pathway and effects on cellular machineries.

Our computational analysis suggested that IGFR might impact specifically on EGFR trafficking, an observation that has not been described so far. This effect might be mediated by direct binding between activated IGFR and EGFR [44] or by the IGFR signaling pathway. Our experiments confirmed that IGF stimulation induced faster endosomal accumulation of EGF, probably by accelerating early endosome fusion. The redistribution of transferrin was not surprising, since the two cargos extensively colocalize in endosomes at early time points [60]. However, IGF-1 stimulation affected specifically EGF trafficking kinetics by inducing both faster uptake and decay (consistent with degradation) without affecting the overall uptake and recycling kinetics of transferrin. Importantly, since these effects were relatively weak, IGFR was not detected as a hit gene in the initial analysis [3]. Only the integrated analysis exploiting the network context revealed its effect on EGF endocytosis.

Other mechanisms of crosstalk among IGFR and EGFR involving receptor cross-activation and heterodimerization [44] or cross-transcriptional regulation [43] have previously been proposed. Our findings extend those reports by uncovering novel aspects of the integration of these signaling pathways at the level of the trafficking system.

### Application to other data sets

The application of the screen to other sources of prior information and other phenotypic data revealed important aspects of the presented method. First, the choice of prior information can significantly affect the quality of the results. Molecular interaction data (as opposed to functional relationships such as co-expression) helped best to reduce noise and improve the mechanistic interpretation of the results.

For the CRISPR screen application, it is difficult to compare the performance of our method to the RIGER screen analysis due to the absence of a positive set. However, we have shown that our method can be successfully applied to diverse kinds of data and can lead to interesting hypotheses to further explore. IMPACT did not reveal modules of genes involved in drug resistance (i.e. increasing cell viability), because they either lack network context or the phenotypic effect is not conserved among interactors. However, it succeeded in identifying molecular machineries responding to the drug (i.e. decreasing cell viability), which could have potential therapeutic applications for the design of co-inhibitors of other genes in the pathway to overcome drug resistance in melanoma.

Despite the aforementioned advantages, there are limitations to this approach. Our network covers less than half of the human protein coding genes. Genes outside the network are ‘inaccessible’ to our analysis. We anticipate that future projects will use other information (such as predicted protein-protein interactions) and also improve the statistical framework. This work therefore just represents the beginning of a gradually more integrated analysis of high-dimensional functional screens in conjunction with network data.

## Methods

### Input data

Both IMPACT-sets and IMPACT-modules work on a high-dimensional dataset generated by functional screens (e.g. knock-down, knock-out, gene editing screen). Here, the phenotype of each gene *g* is measured *m* times, as for instance after knock-down with *m* different oligonucleotides. (Alternative scenarios are for instance targeting the same gene in different individuals or cell lines.) For each single knock-down experiment, the phenotype is described quantitatively by the phenotypic profile *p*, which is an *N*-dimensional vector where each element is a parameter measured. The parameters measured (and thus also the dimensionality of *p*) must be the same for all genes, whereas the number of measurements per gene (*m*) can vary between genes.

The phenotype information *D* is therefore represented as a *PxN* matrix, where rows represent different perturbations (e.g. siRNAs) and columns the different parameters. *N* is the number of parameters and *P* is the total number of phenotypic profiles for all genes, with 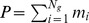, where *m_i_* is the number of different perturbation experiments (e.g. oligonucleotide knock-downs) for the *i_th_* gene and *N_g_* is the total number of genes screened.

Importantly, both methods can work with single phenotypic profiles per gene (*m_i_ = 1*) as well as with multiple oligo profiles per gene (*m_i_*> = 1). Genes in the same data set can have different number of profiles. The screen data *D* needs to be normalized (e.g. z-score or similar), so that parameters have similar impact on the similarity metric during the method search.

### The endocytosis screen

We considered as phenotypic data for our analysis a high-dimensional image-based RNAi screen performed in human HeLa cells [3]. This screen aimed to characterize the loss of function phenotype of each gene involved in the endocytosis of two cargo molecules, transferrin (TF) and the epidermal growth factor (EGF). To this purpose, 40 parameters (Table S1) were quantitatively measured to assess the effect of multiple oligonucleotide knock-downs per gene, with an average of about 7 different si-/esi-RNA reagents per gene. The prior information we used to guide the method search is detailed in the paragraphs “Protein Complexes” and “Protein-Protein Interaction Network”.

### Gene-set-based approach (IMPACT-sets)

The gene-set-based approach tests for the enrichment of a set of related genes for a specific phenotypic profile. The set of genes can be defined based on functional relationships (e.g. pathway co-membership) or physical association (protein complexes). The algorithm consists of two main steps: First, the algorithm identifies a ‘common’ phenotypic pattern that is shared by a maximum number of genes in the set. Then, the statistical assessment is performed via randomizations: for each set with an enriched profile, we generate random sets of the same size and with the same number of profiles per gene in each set. The resulting empirical distribution is used for computing p-values.

#### Enriched pattern estimation

Let us consider a gene set *G* as a set of multiple genes *g_i_*. We denote the number of genes in the set with *n_g_* and the total number of profiles in the whole set with *n_p_*. IMPACT-sets builds the squared similarity matrix *S* of size 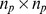 representing the pair-wise similarity between all the profiles *P* present in set *G*. In principle, any similarity measure can be used; here we use the Pearson correlation coefficient. All profiles with correlation scores above a threshold *T* are considered ‘similar’. Only positively correlated profiles are considered. The searching heuristic selects the row (i.e. profile) in *S* having the largest number of genes *n_T_* similar above *T*. If two subsets have equal *n_T_*, the heuristic selects the subset with the maximal sum of correlation values. Note that IMPACT-sets choses the profile with the maximum number of genes having similar profiles. Just maximizing the number of similar profiles in the set may bias the results in favor of genes with large numbers of profiles. Profiles belonging to the selected profiles define the enriched pattern and are used to calculate the reference profile of the set *G* as the median of all selected profiles.

#### Statistical assessment for IMPACT-sets

The statistical assessment is done comparing the number of similar profiles in the observed (real) set to a respective empirical background distribution. The random distributions are computed using random sets that have the same structure (same number of genes *n_g_* and the same number of phenotypic profiles per gene *n_p_g_*) as the real sets. Genes are shuffled between sets, but profiles of the same gene are kept together. In this way we maintained the inherent correlation between profiles of the same gene also in the background distributions. Since the probability of observing *n* similar genes by chance depends on *n_g_* and *n_p_g_*, we generated specific background distribution for each tested complex. Given a set *G*, we performed *N_rand* (5000 in our case) randomizations by assembling complexes with the same structure, i.e. *n_g_* genes and *n_p_g_* profiles per each gene. We then applied the searching step on each of the *N_rand* complexes and we compared the result from the real case to the distribution of results obtained from the randomizations. The p-value is estimated as the fraction of times the same or more profiles are selected as with the observed data.

### Network-based approach (IMPACT-modules)

For the network-based analysis of the phenotypic data, we have implemented a greedy search algorithm (Figure S17). This search method may operate on any network representing genes as nodes and any kind of relationship between genes as (undirected) edges. The method can be applied to phenotypic data where either multiple profiles are available for each gene or when there is a single profile per gene.

The algorithm works in three main steps:

1. **seed selection**: selection of genes (network nodes) as starting points for performing the search;
2. **module expansion**: identification of sub-networks with homogeneous phenotypic profiles;
3. **module assessment**: determination of statistical significance via computing the module p-value.

#### Seed selection

Seed selection iteratively tests each node with at least *k_s* profiles as a potential seed node. The procedure assesses all direct neighbours of a node and the centre-node itself as a ‘gene set’. It then searches for an enriched phenotypic profile in this set using a procedure similar to the method IMPACT-sets described above. We keep as seed nodes only those genes having an enriched phenotypic pattern in the set, i.e. profiles showing a similarity (absolute value of Pearson correlation coefficient) above a threshold *T_s* between each putative seed node and its network neighbours. If such enriched profile exists and if at least one profile of the neighbours is selected, the centre-node will be used as a seed node and the profiles selected in the enriched pattern will be used to calculate the seed profile. Such seed profile is the median of all the selected profiles, where the sign of the profile is taken into account for each neighbour node by swapping the anti-correlated profiles within the node. If any node has profiles both correlated and anti-correlated above the threshold *T_s*, the heuristic selects the profiles that maximize the sum of negative or positive correlation values. In our analysis, we set *k_s* and *T_s* to 2 and 0.8, respectively.

Alternatively, the list of seed nodes can also be created using genes of interest or genes satisfying other properties (e.g. genes exhibiting very strong phenotypes).

#### Module expansion

The module expansion starts from the seed nodes. Neighbours of the current module are assessed by comparing their phenotypic profiles to the seed profile (Pearson correlation). At each step all neighbours having at least *k* phenotypic profiles that are similar above a threshold *T* are added to the module. *k* can either be the minimal number or the minimal fraction of profiles with similarity above the threshold *T* required for the inclusion of the gene in the expansion step. The method requires that the absolute (i.e. negative and positive) correlation between profiles must be equal or above the defined threshold *T*. Different profiles obtained for the same gene must always be positively correlated. Note that, during the module expansion, the seed profile is not changed, i.e. it is not updated after including new genes in the module. This is done to ensure that the seed profile does not start to gradually deviate from the initial profile. The module expansion stops when there are no neighbours satisfying the similarity condition or the maximum module size exceeded 50 expansion steps. Similarly to the set-based analysis, a phenotypic signature for the detected module is computed as the reference profile. This consists of the median of all the selected profiles belonging to the genes in each module. Due to the search strategy this reference profile may deviate slightly, but not strongly, from the seed profile. Since modules can contain both positively and negatively correlated profiles, we accounted for profile sign when computing the median for the reference profile i.e. if the majority of profiles are positively correlated to the seed profile, all the negatively correlated ones are swapped (multiplied by −1), or vice versa.

#### Module assessment

The statistical significance of modules detected is assessed in a semi-analytical way. Our approach computes the probability of observing a specific module by chance, by viewing the module expansion as a stochastic process. At each step of the expansion we compute the probability of including the given number of neighbours under the null-hypothesis that genes (and thus their profiles) are randomly distributed on the network. For this procedure we utilize profile-specific empirical probability distributions that take the inter-dependency of profiles for each gene into account.

Profile-specific distributions are needed to correct for the fact that certain phenotypic profiles are more frequent in the dataset than others. We noticed that certain types (clusters) of profiles are more frequent than others (Figure S5b), which may bias the selection in favor of more frequent profiles. (For the set based approach we did not observe such a frequency bias; Figure S5a). Hence, we created profile-specific background distributions for each seed profile, i.e. the probability of observing a correlated pair of profiles is dependent on the specific phenotypic pattern of the seed profile *p_s_* and the number of profiles per gene. In our case, the number of profiles per gene was very variable and only few genes had many (>7) profiles measured. Thus, it was not possible to generate a background distribution for each possible number of profiles per gene. Instead, we grouped genes with similar (though not identical) numbers of profiles into *n_bin* bins and subsequently randomly drew genes from these bins to generate the background distributions. For each seed node *s* and each of the *n_bin* groups (i.e., *b_1_*, *b_2_*, … *b_n_*), we counted how many times we selected at least *k* profiles where the correlation of the seed profile with the profiles of *N_rand* genes extracted from the bin *b_i_* exceeds the similarity threshold *T*. Therefore, the profile-specific probability for the inclusion of a node belonging to the bin *b_i_* (i.e. with a specific number of profiles) during seed expansion is
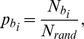
where *i* is the bin index, *N_rand_* is the desired number of random node extractions and
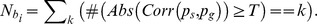
Here, 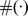 is the function that returns the number of occurrences, *k* is the minimal number of occurrences, *T* is the minimal similarity value required, *p_s_* and *p_g_* are respectively the seed profile and the profile of the gene *g* randomly extracted from the current bin *b_i_*.

The module expansion is considered as a stochastic process made of consecutive states. Each state (i.e. expansion step) consists of the direct neighbors of the current module that contains both, nodes that satisfy the similarity criterion (e.g. minimum 2 profiles similar above threshold) and nodes that do not satisfy it. Consequently, we consider the current neighbors as a set of Bernoulli experiments leading to a binomial distribution for expressing the probability associated with each state of the system. The probability mass distribution and the cumulative distribution function are thus:
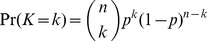
(1)

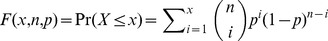
(2)where:

- *n* is the number of Bernoulli experiments i.e. the number of current neighbors in a given state;
- *k* is the number of successful experiments i.e. the number of similar neighbors;
- *n-k* is the number of unsuccessful experiments i.e. the number of not similar neighbors.

For each step of the module expansion we estimate the probability of observing it, i.e. the probability associated with the event of having the current module together with *k* similar and *n-k* dissimilar neighbors. For instance, at the initial step we have a state *x_0_* for which we can compute a probability 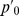; after the inclusion of the first set of similar neighbours, the module is in the state 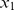, which occurs by chance with a probability 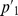 and so on. The probability of a state 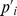 depends on the direct neighbors of the module and on the number of profiles that each of those neighbors has, and it is calculate as:
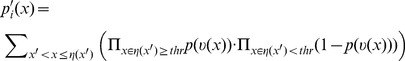
(3)where:

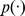
: profile-specific probability distribution 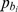 as defined above i.e. probability of observing a correlated pair of profiles, for the number of profiles in bin *b_i_*;
- *x*: is the generic node that is a direct neighbor of *x*′; −*x*′: is the current state of the module and therefore the starting point for evolving into the next state;
- *η*(*x*′): is the function that returns the set of all module neighbors at state *x*′;
- *υ*(*x*′): is the function that returns the number of profiles of each node included in the set produced by *η*(*x*′).

Once the searching procedure has finished, the module p-value is computed combining together the state probabilities:
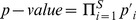
(4)where *S* is the total number of states of a module (i.e. total number of module expansion steps). Equation (4) rests on the assumption that the module expansion steps are independent under the null hypothesis.

### Protein complexes

Protein complexes were taken from CORUM [25], which contains 2,083 experimentally verified mammalian protein complexes (Table S3) of which 1930 had phenotype data from our RNAi screen. Orthologous complexes from non-human species were mapped using the ENSEMBL orthology information.

### Protein-protein interaction network

We assembled an interaction network combining experimentally validated protein-protein interactions from three public sources: HPRD [26] *in vivo* interactions (interactions that are validated in in vivo assays), IntAct [27] and physical protein interactions from KEGG [28]. After removing genes without phenotype information the combined network contains 9,642 nodes and a total of 49,827 interactions.

### Performance curves and AUC

ROC (Receiver Operating Characteristic) curves report the true positive rate (TPR, y-axis) as a function of the false positive rate (FPR, x-axis). 
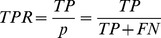
, where TP is the number of true positives and P is the total number of positive, i.e. the sum of true positives plus false negatives (FN). 
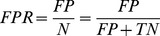
, where FP is the number of false positives and N is the total number of negatives, i.e. the sum of false positives and true negatives (TN). Sensitivity is a synonym for TPR; specificity is 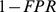.

Precision recall (PR) curves report precision (y-axis) versus recall (x-axis). Precision is defined as 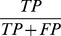 (see definition above). Recall is another name for true positive rate (TPR, see above).

Balanced accuracy is defined as 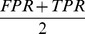. In presence of unequal sized classes and different classification performance on positive or negative sets, the balanced accuracy is a better measure than accuracy [32]. In case of balanced classes it reduces to conventional accuracy 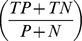. The balanced accuracy curve shows the balanced accuracy value (y-axis) as a function of the p-value threshold (x-axis).

The Area Under the Curve (AUC) is the integral under the ROC curve, calculated by the trapezoidal numerical approximation method. The standard error (sem) was estimated as reported in [33]. To test if the AUC is significantly better than the random case (i.e., AUC = 0.5), we performed the z-test on the quantity 
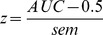
, as in [61]. The statistical assessment of the comparison between two AUCs (Table S10) was performed through stratified bootstrapping (*N* = 1000) by calculating the quantity 
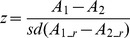
, where *A_1_* and *A_2_* are the two AUCs and *A_1_r_* and *A_2_r_* are the bootstrapped AUC values, described in [62].

### Rescreen comparison

A subset of genes (n = 468) selected with the integrative analysis have been rescreened with 4 new, independent siRNAs (Stealth Select RNAi from Invitrogen) and compared to the primary screen data. All the genes considered for the analysis belong to statistically significant modules and complexes. We used exactly the same cell line and conditions as in the primary screen [3] and we assayed the knock-downs in the same way.

For each gene, four groups of profiles have been considered: (1) the module reference profile, (2) the oligo profiles selected by our method, (3) the oligo profiles excluded by our method, and (4) the new oligo profiles in the rescreen. We computed the distribution of the pairwise Pearson correlations between each one of the groups (1)-(2)-(3) and group (4) and compared the three cumulative distributions of the correlation values. Two different non-parametric tests, the Kolmogorov-Smirnov and the Mann-Whitney U test, were used to assess the statistical significance of the differences between pairs of distributions.

### IGFR stimulation experiments

HeLa cells were grown in DMEM supplemented with 10% FCS and 24 h prior to the experiment cells were plated in 96 well plates to reach approximately 80% confluence on the day of the experiment.

Each experimental condition was repeated twice in the plate layout, and each experiment was repeated 4 and 5 times for the co-pulse-chase and the co-pulse, respectively.

#### Continuous pulse experiments

In step co-pulse experiments 100 ng/ml A488-EGF and 5 µg/ml A647-TF were co-pulsed with (50 ng/ml or 250 ng/ml) or without IGF-I (Peprotech) for the indicated time points (0-1-3-5-7.5-10-15-20-25-30-60 minutes).

#### Pulse chase experiment

In co-pulse-chase experiments 100 ng/ml A488-EGF and 5 µg/ml A647-TF were co-pulsed with (50 ng/ml or 250 ng/ml) for 10 minutes and then chased in medium containing 50 ng/ml or 250 ng/ml of IGF-I for the indicated time points (0-10-20-30-45-60-90-120-180 minutes). Cells were then washed 2 times with PBS fixed and stained as previously described [3].

#### Image acquisition and analysis

Triple color images were collected in a fully automated and unbiased manner using a spinning disk confocal microscope (OPERA, Evotec Technologies-Perkin Elmer) as previously described [3]. For the co-pulse-chase experiment, 14 images per well, containing in average 25 cells, were collected to have more than 160 images and 4200 cells analyzed per condition. For the co-pulse experiment, 14 images per well, containing in average 28 cells, were collected to have more than 240 images and 6700 cells analyzed per condition. Image and data analysis were performed [3] with the Motiontracking software [3], [29].

## Supporting Information

Figure S1 **Ambiguity of phenotypic profiles for different siRNA oligonucleotides.** X-axes show deviation of channel 1 (EGF) parameters (x-labels ‘C1’, left part) and channel 2 (transferrin, TF) parameters (x-labels ‘C2’, right part) from average in terms of z-scores. A large deviation from zero suggests a strong effect of the RNAi knock-down on the respective readout. See Table S1 for details. **Left**: Profiles obtained for different siRNAs targeting components of the AP2 complex. **Right**: Profiles selected by utilizing co-complex membership information. Bold red line shows ‘reference profile’ obtained as the median of the selected profiles. One would expect that knock-down of any component of the AP2 complex results in similar phenotypic signatures. However, observed profiles are inconsistent due to assay noise and off-target effects. After selecting the most enriched signature across all components of the complex (right) a consistent profile is obtained with strong changes of channel 2 parameters (transferrin) and no significant effects on channel 1 (EGF).
(PDF)

Figure S2 **Correlation of profiles targeting the same gene.** Distribution of Pearson correlation coefficients between profiles obtained from siRNAs and esiRNAs targeting the same gene. In the ideal case, where noise and OTE are low, phenotypic profiles obtained with different oligos should be highly correlated.
(PDF)

Figure S3 **Parameter reproducibility in the primary screen and rescreen data set.** The Pearson correlation coefficients between each parameter composing the multi-parametric profiles have been computed for the primary screen (black bars) and the rescreen (red bars). Upper panel: bar graph showing the correlation value on the y-axis for each one of the 40 parameters on the x-axis (Suppl. Table S1). Lower panel: cumulative distribution function of the correlation values shown in the upper panel. The comparison has been done on a subset of more than 1000 different genes where multiple runs of the experiment have been acquired. The difference between the two curves (black and red cumulative distributions in the lower panel) is statistically significant (p-value of 0.0017 with the Mann-Whitney U test).
(PDF)

Figure S4 **Distribution of Pearson correlation coefficient for all oligo pairs of the same gene.** Above: normalized histograms. Below: cumulative distributions of the histograms above. Red curves are distributions for all genes in the re-screen subset. Black curves are distributions for all genes in the primary screen total set.
(PDF)

Figure S5 **Heat maps after hierarchical clustering of reference profiles.** Hierarchical clustering (Pearson correlation used as distance measure and average linkage method for computing the distance between clusters) of protein complex reference profiles (a) and network seed set profiles (b). Rows are multi-parametric profiles; columns are different parameters labeled with different numbers from 1 to 40 (Suppl. Table S1).
(PDF)

Figure S6 **ROC, PR and BACC curves for IMPACT-sets.** Row (a): comparison of different searching thresholds. Row (b): comparison IMPACT-sets to the analysis done using a single profile (average and mode of the original oligo profiles).
(PDF)

Figure S7 **ROC, PR and BACC curves for IMPACT-modules.** Row (a): comparison of different minimal number of profiles *k*. Row (b): comparison of our integrative analysis to the same analysis done using a single profile. Row (c): comparison between different searching thresholds (*T*).
(PDF)

Figure S8 **ROC, PR and BACC curves for the gene degree analysis.** Comparison of classification performances of the topological information alone e.g. gene degree versus IMPACT-modules (*T* = 0.7, *k* = 3).
(PDF)

Figure S9 **Comparison of IMPACT-modules with the published endocytosis screen analysis.** ROC, PR and BACC curves showing an improved detection of endocytosis genes (GO terms, *n* = 289) for network modules when compared to the previously published analysis based on the chi-square statistics (chi-mode in the legend). The red asterisk (*) reports the performance using the hit-list definition of the previous analysis [1], which was based on a combined evaluation of the phenotypic strength (chi-square) and phenotypic specificity (Phenoscore). The values are calculated by the true and false positive rates based on a significance threshold of 0.05, as in the published hit list. IMPACT can recover a higher number of true positives (ROC curve) at the same false positive rate as the previous analysis.
(PDF)

Figure S10 **Recovery of known endocytosis genes by IMPACT** ***versus*** **the original publication.** Venn diagrams reporting counts of endocytosis genes (based on GO annotation) selected as significant by IMPACT (blue circle) and previously published as screen hits [1] based on the Chi-square of the mode profile (red circle). To perform a balanced comparison, we selected the 2,720 significant (p-value< = 0.1) genes from IMPACT-sets and IMPACT-modules (Table S5) and the top 2,720 genes from the sorted Chi-square list. Out of the 36 genes found in the top Chi-square list and not detected by IMPACT, 26 could be mapped on our interaction data. The higher number of endocytic genes recovered specifically by IMPACT (79) compared to the ones missed (36, of which 26 mapped), shows that it has better sensitivity/specificity trade-off, as also highlighted by the AUC analysis.
(PDF)

Figure S11 **IGF-1 co-stimulation experiments: pulse-chase experiment of labeled EGF and transferrin in presence of IGF-1.** (**a**) Temporal profile of the total vesicular intensity calculated for the EGF-positive endosomes, with different concentration of IGF-1 (dark red: 250 ng/ml; light red: 50 ng/ml; black: no IGF-1 (0 ng/ml), or control), normalized by the time point 30′-chase to better visualize the monotonously decaying phase. (**b**) Temporal profile of the total vesicular intensity calculated for the TF-positive endosomes, with different concentration of IGF-1 (dark green: 250 ng/ml; light green: 50 ng/ml; black: no IGF-1 (0 ng/ml), or control), normalized by the time point 0′-chase to better visualize the monotonously decaying phase. Normalized experimental points (dots plus error bars) and the fitted curves (dashed lines), obtained by fitting the decaying exponential function f(x) = A*e^−t/τ^, are shown in both panels (a) and (b). The insets display the estimated time constants of the respective fitting.
(PDF)

Figure S12 **Distribution analysis for the IGF-1 co-stimulation experiment.** (**a**) The integral intensity (i.e., the integral of the intensity of the fitted object, representing per-endosome cargo content) is calculated per-each endosome. From here, a histogram is built showing how many endosomes are counted (y-axis) for each bin of mean vesicular integral intensity (x-axis). The two curves (black and red) represent two arbitrary conditions to be compared. (**b**) Starting from the mean integral intensity distribution shown in panel (a), it is possible to calculate the total vesicular intensity distribution shown here, by multiplying each bin of mean integral intensity (x-value) by the number of counts in that bin. This distribution displays how much cargo is contained in different sub-population of endosomes, that contain different mean cargo amount. (**c**) Bin count difference of the two distributions shown in panel (a): it is here visible that low cargo containing endosomes are depleted in the “red” condition, whereas bigger endosomes containing more cargo are enriched. The continuous grey line represents smoothing by moving average. (**d**) Distribution of the total vesicular intensity difference per bin, calculated subtracting the two curves in panel (b): it is here visible that endosomes from the “red” condition contain more cargo than the ones from the “black” condition, especially for high bins (>10^4^) of mean integral intensity (i.e., big endosomes containing a lot of cargo). The continuous grey line represents smoothing by moving average. The visualization depicted in panel (d) is what has been used in Figures 6.
(PDF)

Figure S13 **Details of the phenotypic space figure for complexes.** Proteins being part of the complexes are shown in the respective insets. Asterisks (*) indicate genes having at least one profile correlated with the profile shown in the insets in Figure 4.
(PDF)

Figure S14 **Details of the phenotypic space figure for modules.** Here we report the graphic representations of the modules of Figure 5. We used Cytoscape for the module visualization.
(PDF)

Figure S15 **Comparison of IMPACT-modules with other methods on an independent list of genes.** ROC, PR and BACC curves for the direct comparison of IMPACT-modules with other methods in classifying an independent list not used for parameter tuning, to account for potential overfitting. We used the merged list of Rab5 effectors [2] and of proteins with endocytic domains related to endocytosis (PX, FYVE, BAR, TBD and VPS9, [1]), comprising of 306 members, of which 213 are present in the interaction network (the overlap with the endocytosis GO annotation gene list was just 62 genes). IMPACT-modules (both for *T* = 0.7, *k* = 3 and *T* = 0.7, *k* = 2) out-performed the other approaches on this new list (shown here) as well as on the union of the two, the GO terms list and the RAB5 effectors and domains list (not shown). The red asterisk (*) in all three panels indicates single values relative to the hit list definition of the previous analysis [1], which was based on a combined evaluation of the phenotypic strength (chi-square) and phenotypic specificity (Phenoscore). The values are calculated by the true and false positive rates based on a significance threshold of 0.05, as in the published hit list. At equal false positive rate value as the hits list from the previous analysis, IMPACT can recover a higher number of true positives (ROC curve).
(PDF)

Figure S16 **Assessment of statistical significance for the set-based analysis.** For the set-based analysis, significance is determined through appropriate randomizations that take into account the genes and profiles number in each complex: 1) gene labels are permuted across the entire dataset keeping together profiles belonging to the same gene; 2) complexes subunits are reshuffled only with random subunits having a comparable number of profiles.
(PDF)

Figure S17 **Visual description of IMPACT-modules pipeline.** A) Flowchart of the network-based approach. Parallelograms Indicate input/output steps, rectangles describe processing steps. Different sections described in the main text (Methods) are indicated on the left. The pipeline takes two input files, the phenotype data and the prior information (interaction data), see Input Data in Methods. B) Description for the parameters reported on the right of the flowchart. A similar flowchart applies to the IMPACT-set module, with the only following difference that there is no seed selection step, as each set (i.e. protein complex) is considered for the analysis.
(PDF)

Figure S18 **Distribution of pairwise similarity for oligonucleotides of the same gene.** a) Distribution of the number of profiles similar above *T* = 0.7 within the same gene (*n_s*). b) Distribution for the fraction of profiles similar above threshold *T* = 0.7 within the same gene, calculated as *n_s*/*n_tot*, with *n_tot* the total number of profiles per gene.
(PDF)

Figure S19 **Reference profiles better match re-screen profiles than original phenotypic profiles.** Profiles selected by IMPACT (blue curves) compared to all the oligonucleotide profiles in the old screen data (left) and the new rescreen data (right). Three examples are shown (top to bottom): PDPK1, MLC1, IGF1R. X-axes: parameter index as described in Table S1. Y-axes: normalized parameter value. For further description of plots see Figure S1.
(PDF)

Table S1 **List of parameters used in the RNAi screen assay [1] (prev. page).** The first two columns describe respectively the label and the description for the parameter groups used as summarized graphical representation (Figures 4 and 5). The third column enumerates all the parameters constituting each group. The numbers in parentheses indicate the parameter index for EGF and TF, respectively. All 40 parameters have been used in the integrative analysis; but the parameters “background intensity” and “colocalisation” at the end of the table have not been used for the summarized graphical representation.
(PDF)

Table S2 **GO terms related to endocytosis.** GO terms used for assembling the positive reference set. Genes annotated for one or more of these terms were considered as positives (387). The negative set was assembled considering genes that are annotated with functions other than endocytosis (21,585). Of those, 293 positive and 9,929 negative genes are represented in the network and 133 positive and 2,735 negative ones are in the sets.
(PDF)

Table S3 **CORUM complexes.** CORUM database comprehends manually curated protein complexes from Human and from other mammalian organisms. In the table the number of complexes per organisms is reported. The total number from all species is 2,083.
(PDF)

Table S4 **Results of IMPACT-sets on CORUM complexes and IMPACT-modules on the combined interaction network.** Classification performances are reported in terms of AUCs (of the ROC curves) of IMPACT-sets (left) and IMPACT-modules (right) applied on CORUM complexes and on the combined interaction network respectively. AUCs are reported for different similarity thresholds (*T*) and for the analysis based on a single profile (i.e. average of the original oligonucleotide profiles and mode of the original oligonucleotide, cases single-avg and single-mode). The table on the right shows AUCs obtained by using IMPACT-modules with different similarity thresholds (*T*) and different minimum number/percentage of profiles (*k*), labeled as *T* - *k*. Legend: AUC = area under the ROC curve; sem = standard error of the mean; p-values = p (AUC)>0.5.
(PDF)

Table S5 **Number of genes selected by IMPACT.** Number of unique genes identified by both IMPACT-sets (*T* = 0.7) and IMPACT-modules (*T* = 0.7, k = 2). **Left column:** total numbers of genes selected by IMPACT in any module/set. **Right column**: number of genes in modules/sets with p-values< = 0.1. 0.7-single-mode and 0.7-single-avg represent the analysis done by considering a single profile i.e. mode and average profile computed out of the original oligo profiles of each gene.
(PDF)

Table S6 **Classification results obtained considering protein complexes as interaction network.** The table summarize the results obtained by running IMPACT-sets and IMPACT-modules on protein complexes, with *k* = 3 (minimal number of similar profiles). Isolated network: protein complexes are converted to binary protein interactions with the matrix model (all interacting with all) and they constitute an interaction network on their own. Other network context: as in isolated network, but the complex interaction network is added to the whole combined network used for IMPACT-modules.
(PDF)

Table S7 **Classification results obtained considering different subsets of parameters in the phenotypic vector.** Different groups of parameters (left column, Table S1 for details) were removed one by one from the original phenotypic data. G1 and G2 parameter groups were combined together because: 1) they represent linked biological features (endocytic uptake); 2) to have the same number of excluded parameters as the other cases (6 each). IMPACT-modules was run (*T* = 0.7, *k* = 3) and the AUC on the selected modules was measured for each case (right column).
(PDF)

Table S8 **Comparison of classification performance of IMPACT-modules with other methods.** In the table are reported Area Under the Curve (AUC) and standard error of the mean (sem) values relative to the analysis conducted with IMPACT-modules (grey rows) and relative to analysis performed using alternative methods (MATISSE, JActiveModules) that integrate network information with phenotypic data and are based on single profile (MATISSE) or on single values (JActiveModule). Chi-mode and Chi-avg denote the classification of the chi-square statistic calculated on the mode and average profile. Degree denotes the classification performances obtained by ranking the degree of network nodes.
(PDF)

Table S9 **Comparison of classification performances of IMPACT-sets with other methods.** Comparison of classification performances of IMPACT-sets with a different approach that uses only phenotypic information (e.g. chi-square statistic calculated on the mode and average profile of the oligo profiles of each gene).
(PDF)

Table S10 **Classification performances of IMPACT-modules and other methods on different positive sets.** Classification performances of IMPACT-modules measured on different lists and compared to other methods. Lists: 1) endocytosis GO terms list (yellow); 2) RAB5 effectors and proteins with domains related to endocytosis (red); 3) same as 2), excluding genes common to GO (cyan); 4) the union of both 1) and 2) (green). Legend: AUC = area under the ROC curve; sem = standard error of the AUC estimation; p(AUC) = probability that the AUC is higher than the random 0.5 case (z-test); p(diff) = probability that difference between AUCs of the reference method (0.7–3 or 0.7–2) and the compared case is significant (z-test of differences of stratified bootstrapped values).
(PDF)

Table S11 **Enrichment list for network modules.** List of molecular pathways (KEGG) enriched among the modules with low p-value<0.1. The enrichment was evaluated with the hypergeometric test by using the DAVID web-based Bioinformatics resources [3], [4]. The background distribution was built considering the entire network.
(PDF)

Table S12 **Complete list of endocytosis significant genes rescued by IMPACT.** 92 endocytic genes (GO annotation) were found in significant modules and/or protein complexes (p-value< = 0.1) by IMPACT-sets (*T = 0.7*) and IMPACT-modules (*T = 0.7*, *k = 2*) and were not hits in the previous screen analysis [1]. * indicates genes in significant modules/sets.
(PDF)

Table S13 **Comparison of classification performance of IMPACT-modules depending on similarity measure and seeding.** In the table are reported Area Under the Curve (AUC), standard error of the mean (sem) and p-values (probability that the AUC is higher than the random 0.5 case; z-test) relative to the analysis comparing different similarity measures for both modules and sets (absolute versus positive correlation) and different seeding for modules only. When not indicated, IMPACT-modules is run with default seeding parameters *T_s* = 0.8 and *k_s* = 2, as reported in the main text. The cases in bold are the ones chosen for the follow-up analysis in the main text.
(PDF)

Table S14 **Comparison of different networks used as prior information in IMPACT-modules.** Different networks used as prior information for IMPACT-modules. For STRING, different evidence levels (experimental, all and co-expression) and different confidence levels (400, 700 and all (> = 0), respectively) were used. Network features and module seeding/searching summary are reported.
(PDF)

Table S15 **Classification performance of IMPACT-modules with different interaction networks on the endocytosis GO terms.** Legend: AUC = area under the ROC curve; sem = standard error of the AUC estimation; p(AUC) = probability that the AUC is higher than the random 0.5 case (z-test).
(PDF)

Table S16 **Enrichment analysis for the autophagy genes.** Legend for columns, from left to right: scoring method; number of autophagy genes in the scoring list; total number of autophagy genes assessed by the scoring method (175 for the entire screen, of which 161 mapping on the interaction network); number of genes selected as significant by the scoring method; total number of genes assessed by the scoring method; p-value of enrichment of autophagy genes in the scoring hit-list, calculated through Fisher's exact test. # gene sig. = gene in significant modules (p-value< = 0.1).
(PDF)

Table S17 **Classification performance for the autophagy genes.** Legend: AUC = area under the ROC curve; sem = standard error of the AUC estimation; p(AUC) = probability that the AUC is higher than the random 0.5 case (z-test). Abbreviation: avg. = average of profiles from different replicates.
(PDF)

Table S18 **Gene Ontology biological process annotations enriched in the CRISPR-Cas9 screen analysis.** Column legend (left to right): name summarizing the different GO categories in the cluster; enrichment score, calculated as −Log (p-value), where p-values is the one in the next column; geometric mean of enrichment p-values for terms in the same cluster; geometric mean of the fold enrichment of different terms in the same cluster; geometric mean of the p-value corrected for multiple hypothesis (Benjamini correction).
(PDF)

Table S19 **KEGG pathways annotations enriched in the CRISPR-Cas9 screen analysis.** Column legend (left to right): name summarizing the different GO categories in the cluster; enrichment score, calculated as −Log (p-value), where p-values is the one in the next column; geometric mean of enrichment p-values for terms in the same cluster; geometric mean of the fold enrichment of different terms in the same cluster; geometric mean of the p-value corrected for multiple hypothesis (Benjamin correction).
(PDF)

Table S20 **Genes in the Melanoma KEGG pathway selected by IMPACT for the CRISPR-Cas9 screen analysis.** Full list of the 35 genes selected by IMPACT belonging to the Melanoma KEGG pathway annotation.
(DOCX)

Table S21 **Genes in the MAPK KEGG pathway selected by IMPACT for the CRISPR-Cas9 screen analysis.** Full list of the 97 genes selected by IMPACT belonging to the MAPK KEGG pathway annotation.
(DOCX)

Text S1 **Dissecting the function of related protein complexes.**
(PDF)

Text S2 **Application to other data sets.**
(DOCX)

Text S3 **References for the supplementary materials.**
(PDF)
