# Supplementary figures and images for: Revealing Molecular Mechanisms by Integrating High-Dimensional Functional Screens with Protein Interaction Data
Source: PLoS Comput Biol. 2014 Sep 4;10(9):e1003801. doi: 10.1371/journal.pcbi.1003801 (PMC4154648; doi:10.1371/journal.pcbi.1003801)

a

AP2-complex – all mapped phenotypic profiles

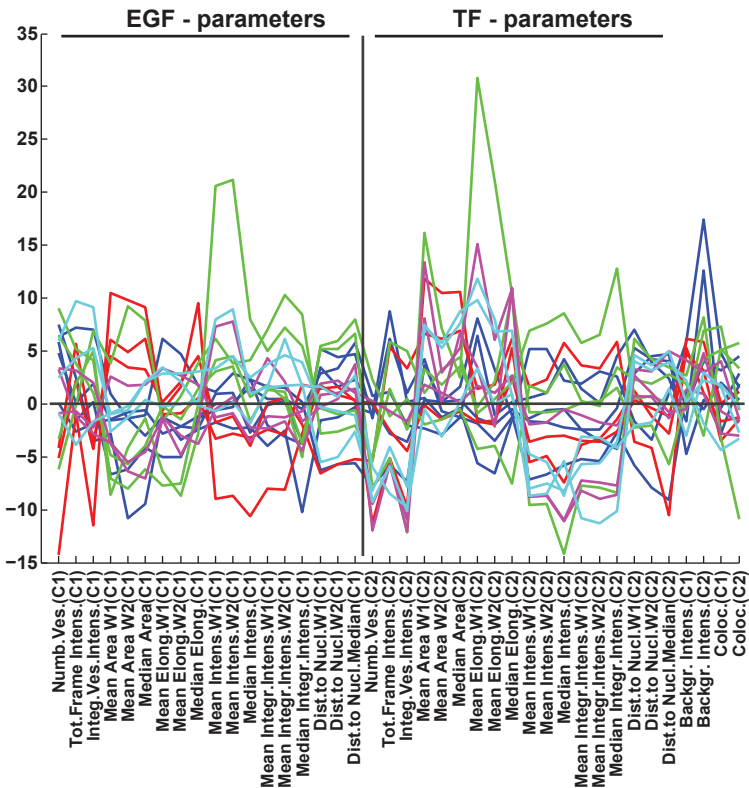

b

AP2-complex – selected phenotypic profiles

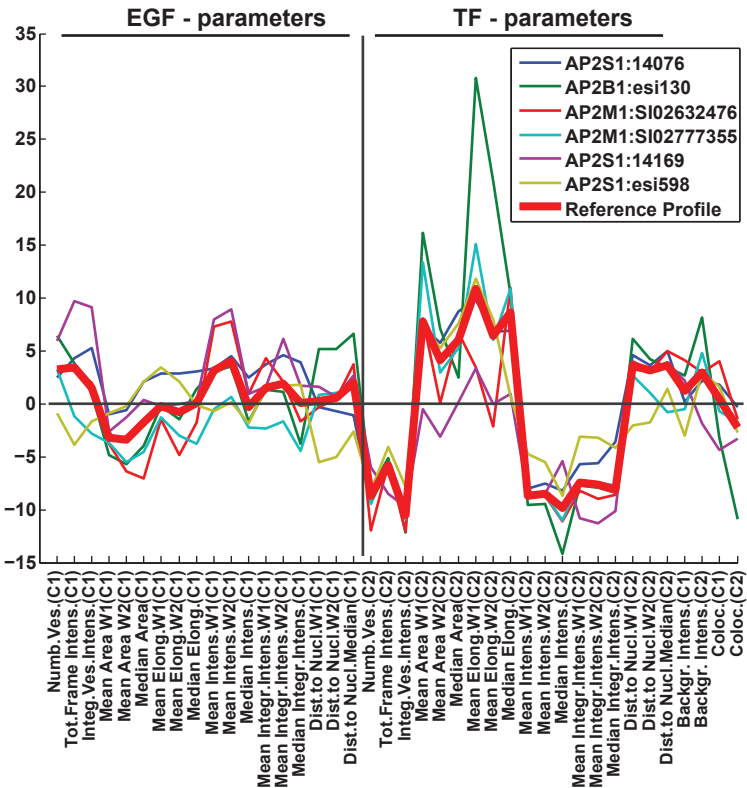

Supplement: Figure S1 — Ambiguity of phenotypic profiles for different siRNA oligonucleotides. X-axes show deviation of channel 1 (EGF) parameters (x-labels ‘C1’, left part) and channel 2 (transferrin, TF) parameters (x-labels ‘C2’, right part) from average in terms of z-scores. A large deviation from zero suggests a strong effect of the RNAi knock-down on the respective readout. See Table S1 for details. Left: Profiles obtained for different siRNAs targeting components of the AP2 complex. Right: Profiles selected by utilizing co-complex membership information. Bold red line shows ‘reference profile’ obtained as the median of the selected profiles. One would expect that knock-down of any component of the AP2 complex results in similar phenotypic signatures. However, observed profiles are inconsistent due to assay noise and off-target effects. After selecting the most enriched signature across all components of the complex (right) a consistent profile is obtained with strong changes of channel 2 parameters (transferrin) and no significant effects on channel 1 (EGF). (PDF) [file pcbi.1003801.s001.pdf]

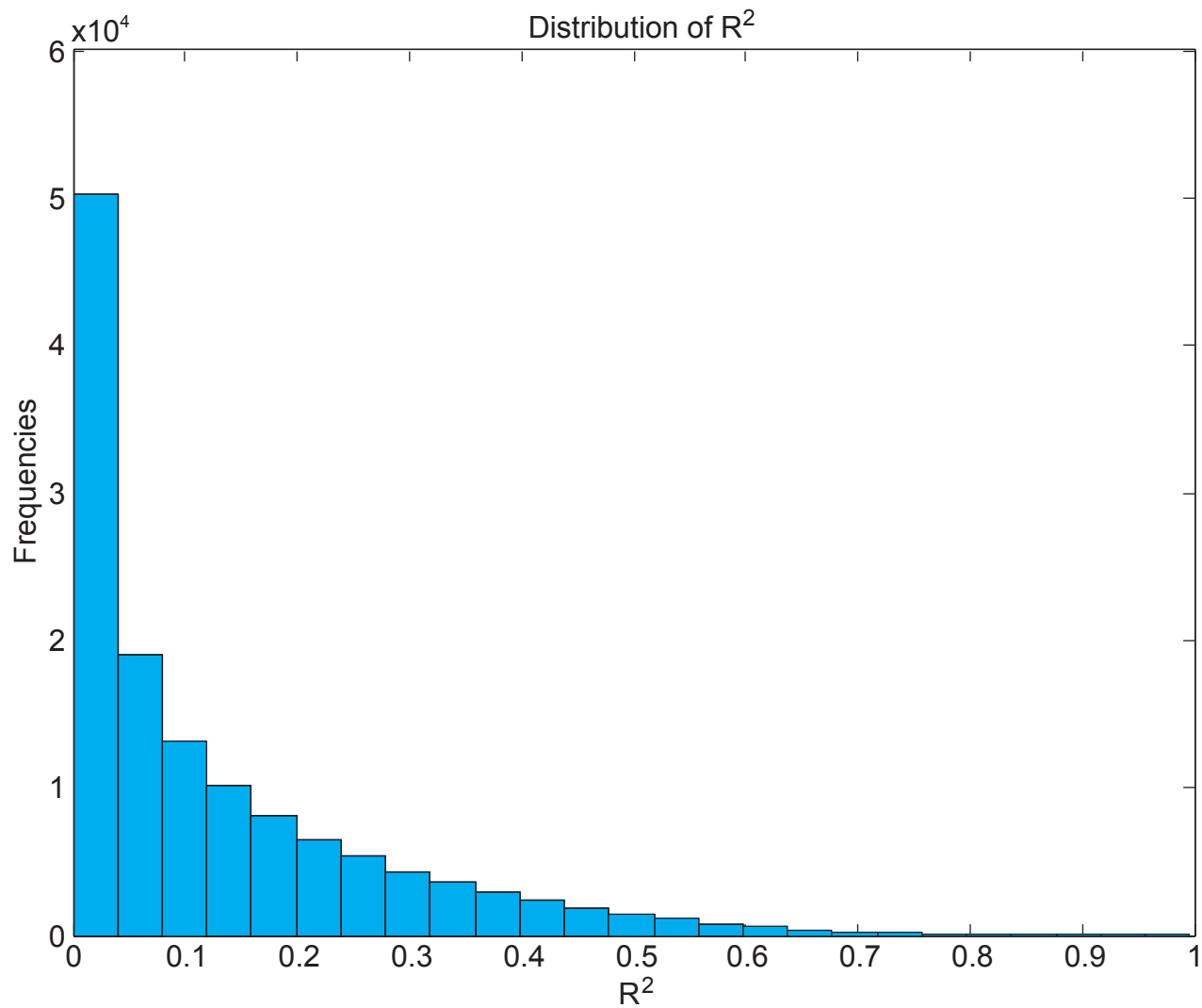

Supplement: Figure S2 — Correlation of profiles targeting the same gene. Distribution of Pearson correlation coefficients between profiles obtained from siRNAs and esiRNAs targeting the same gene. In the ideal case, where noise and OTE are low, phenotypic profiles obtained with different oligos should be highly correlated. (PDF) [file pcbi.1003801.s002.pdf]

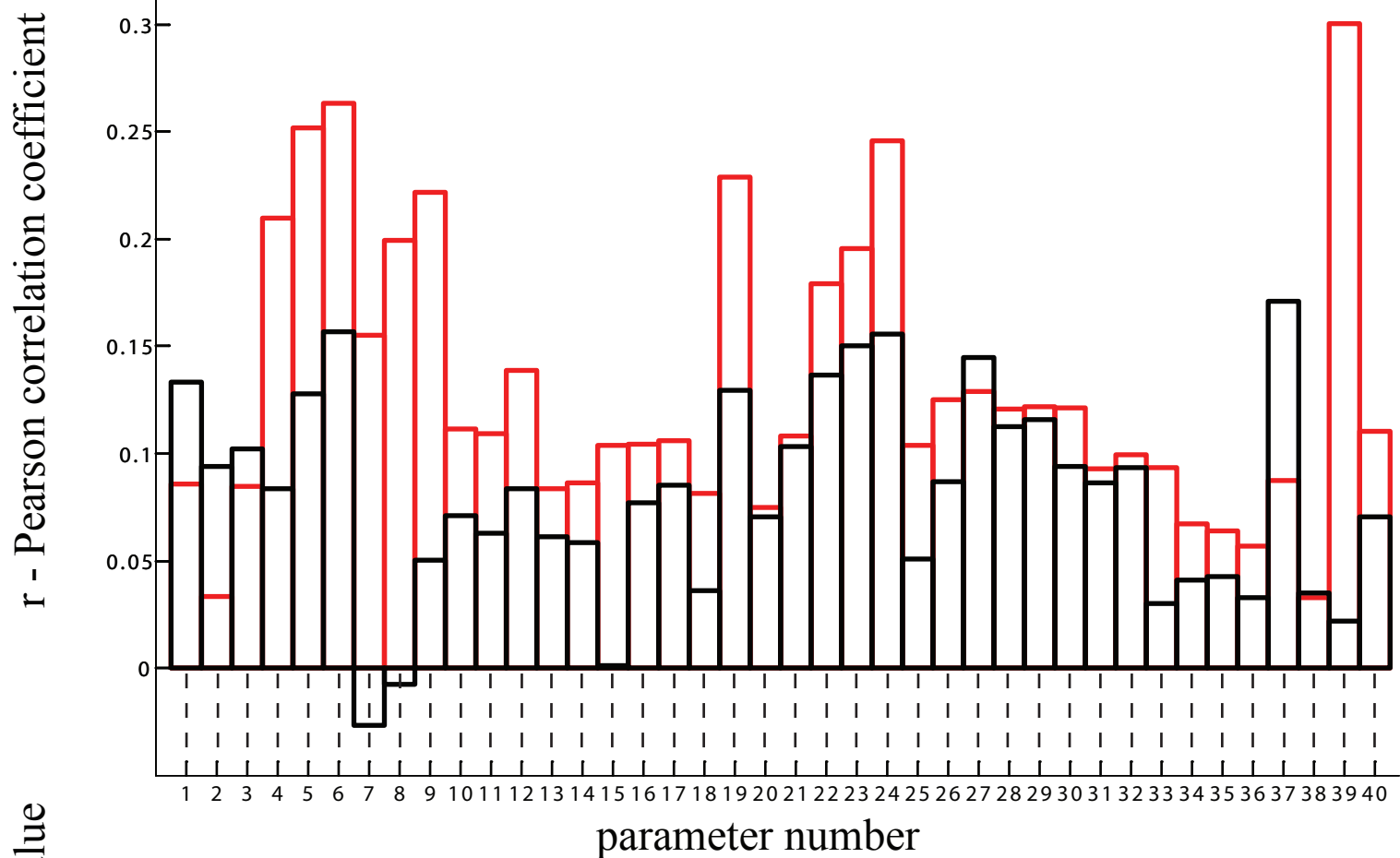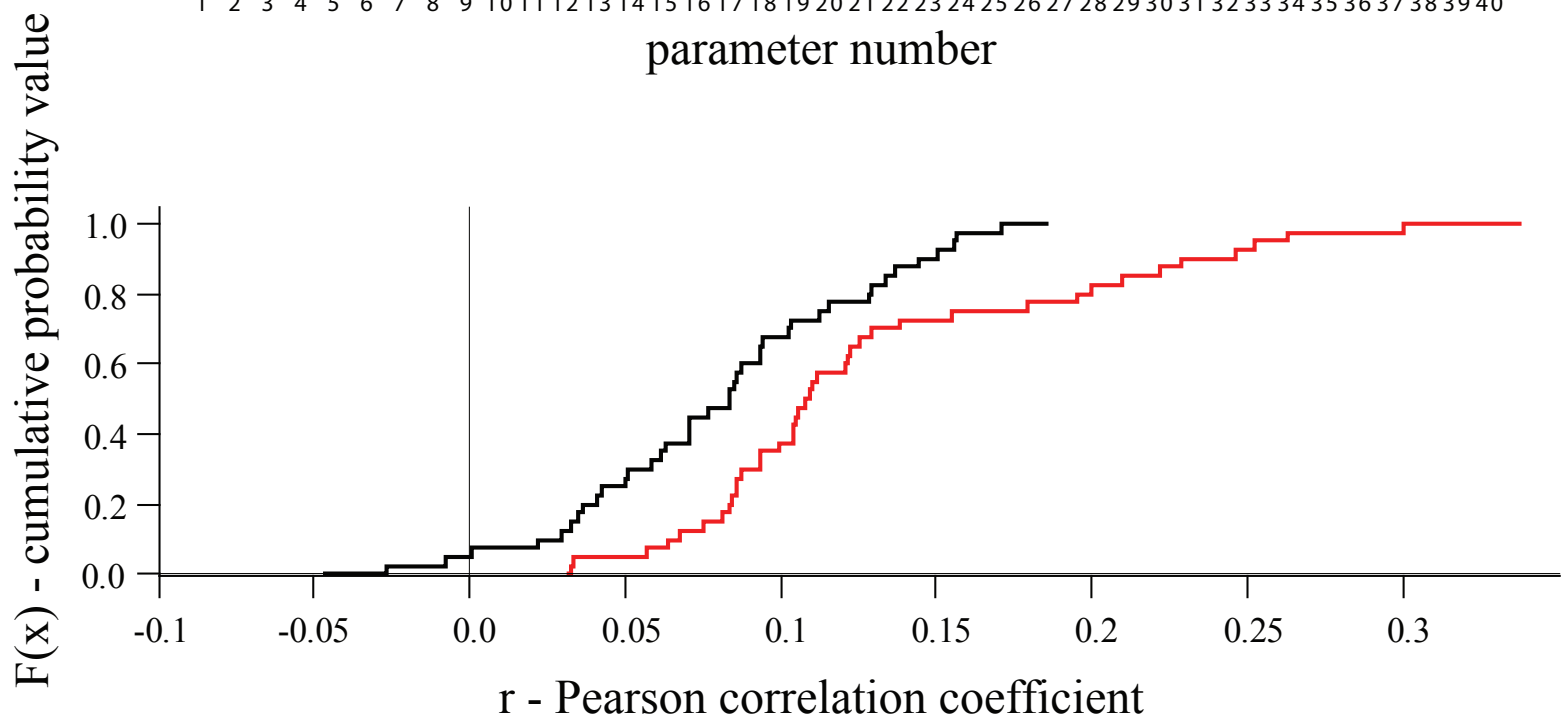

Supplement: Figure S3 — Parameter reproducibility in the primary screen and rescreen data set. The Pearson correlation coefficients between each parameter composing the multi-parametric profiles have been computed for the primary screen (black bars) and the rescreen (red bars). Upper panel: bar graph showing the correlation value on the y-axis for each one of the 40 parameters on the x-axis (Suppl. Table S1). Lower panel: cumulative distribution function of the correlation values shown in the upper panel. The comparison has been done on a subset of more than 1000 different genes where multiple runs of the experiment have been acquired. The difference between the two curves (black and red cumulative distributions in the lower panel) is statistically significant (p-value of 0.0017 with the Mann-Whitney U test). (PDF) [file pcbi.1003801.s003.pdf]

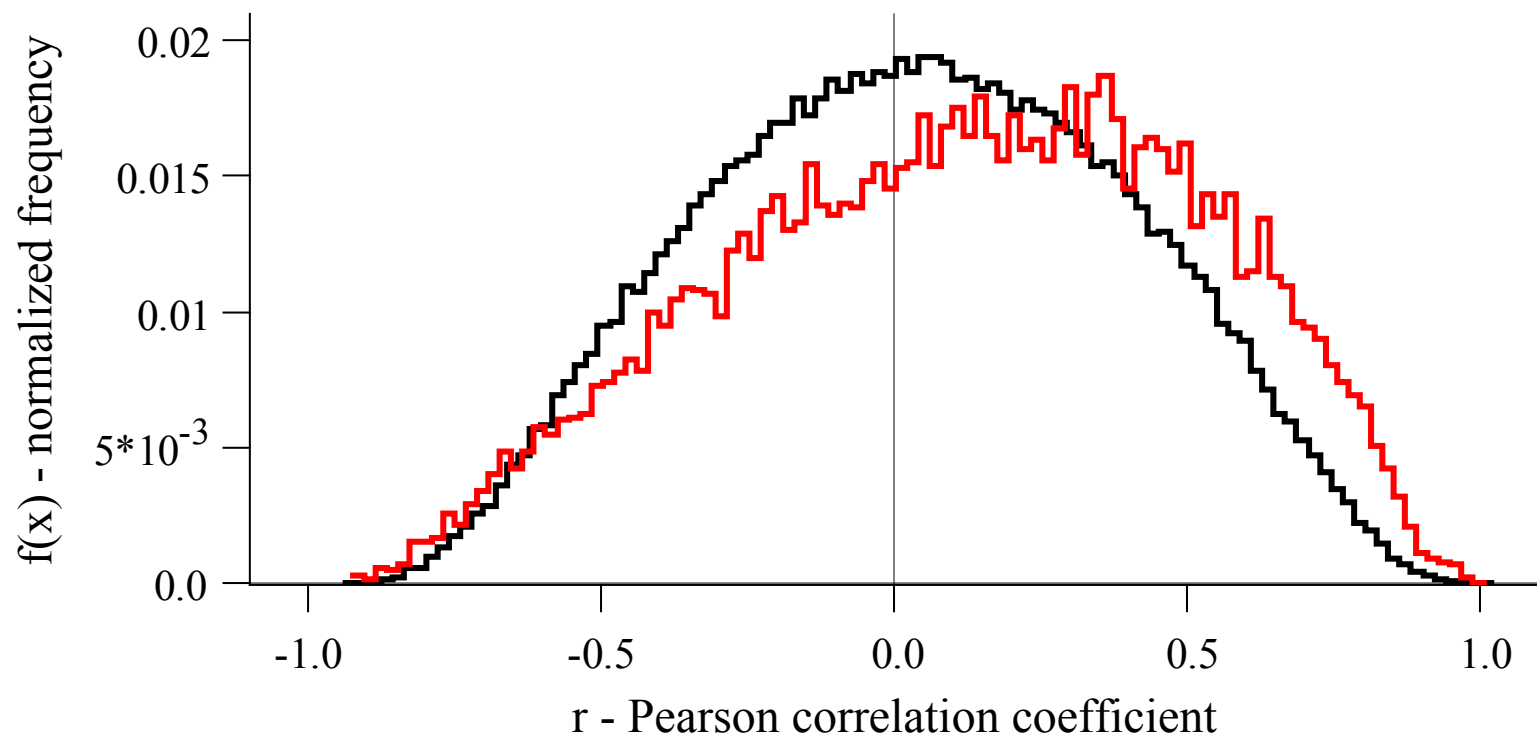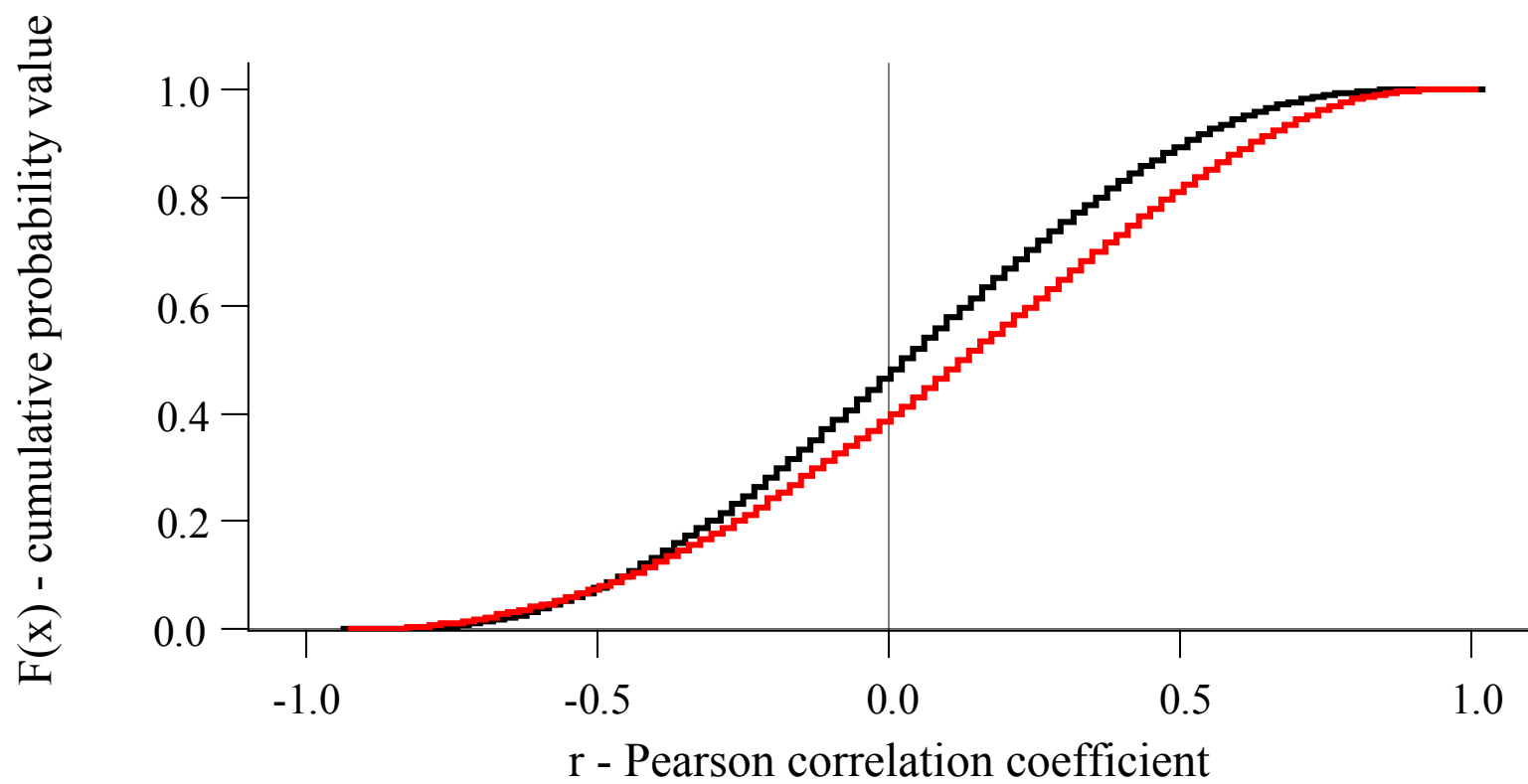

Supplement: Figure S4 — Distribution of Pearson correlation coefficient for all oligo pairs of the same gene. Above: normalized histograms. Below: cumulative distributions of the histograms above. Red curves are distributions for all genes in the re-screen subset. Black curves are distributions for all genes in the primary screen total set. (PDF) [file pcbi.1003801.s004.pdf]

a

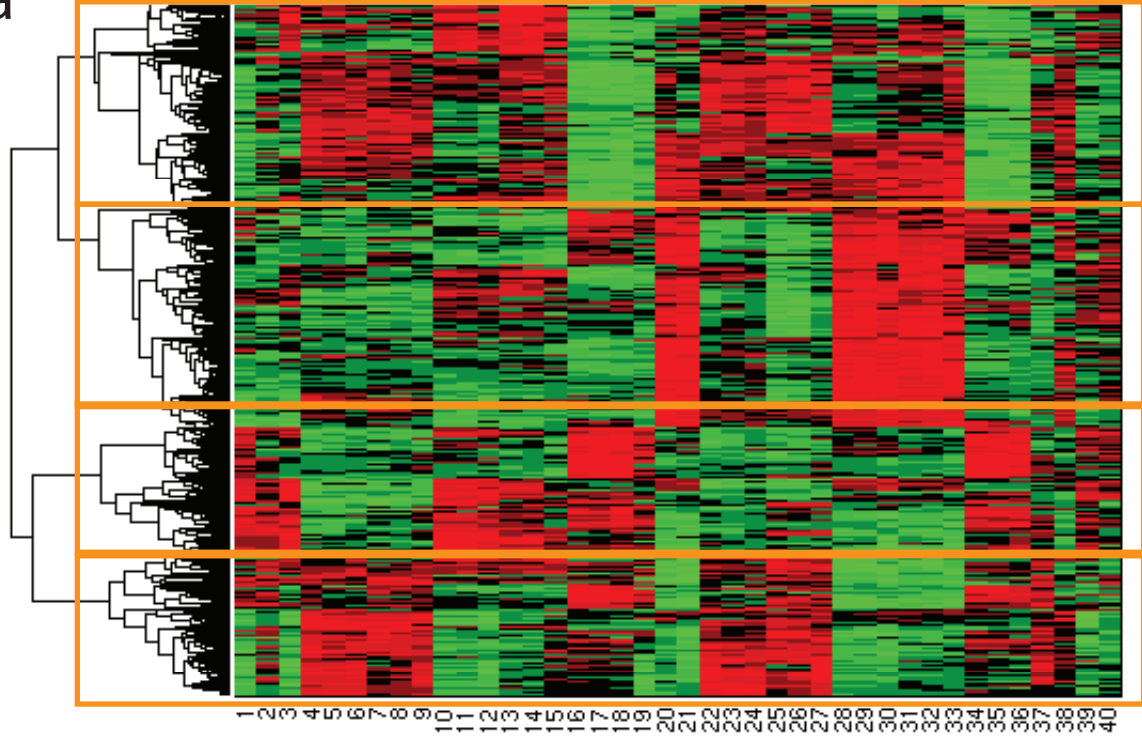

b

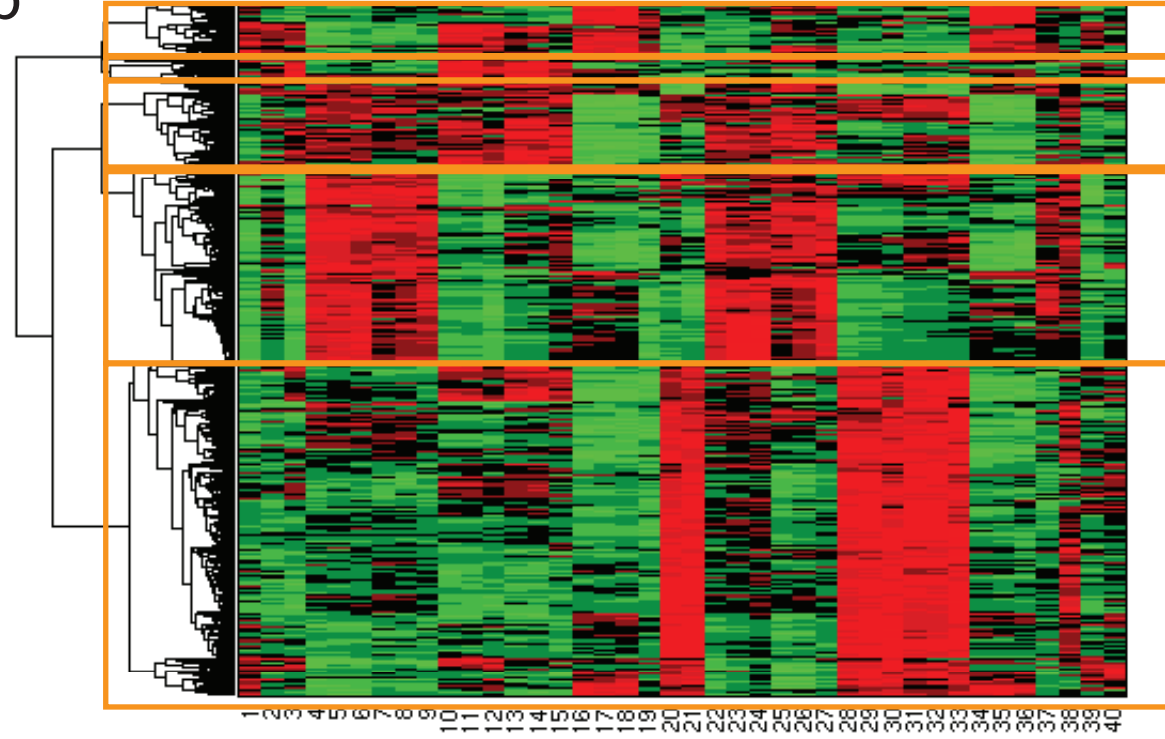

Supplement: Figure S5 — Heat maps after hierarchical clustering of reference profiles. Hierarchical clustering (Pearson correlation used as distance measure and average linkage method for computing the distance between clusters) of protein complex reference profiles (a) and network seed set profiles (b). Rows are multi-parametric profiles; columns are different parameters labeled with different numbers from 1 to 40 (Suppl. Table S1). (PDF) [file pcbi.1003801.s005.pdf]

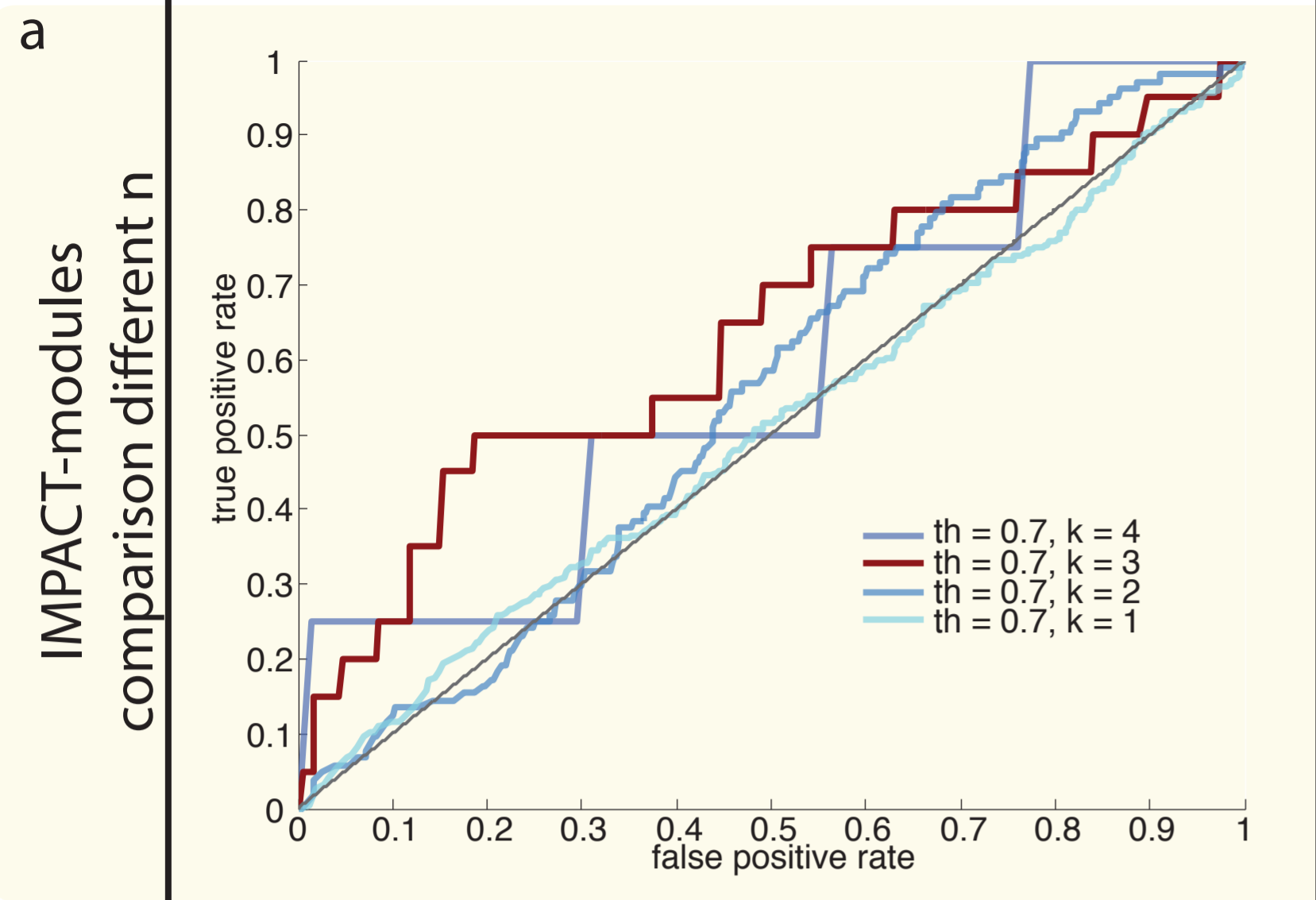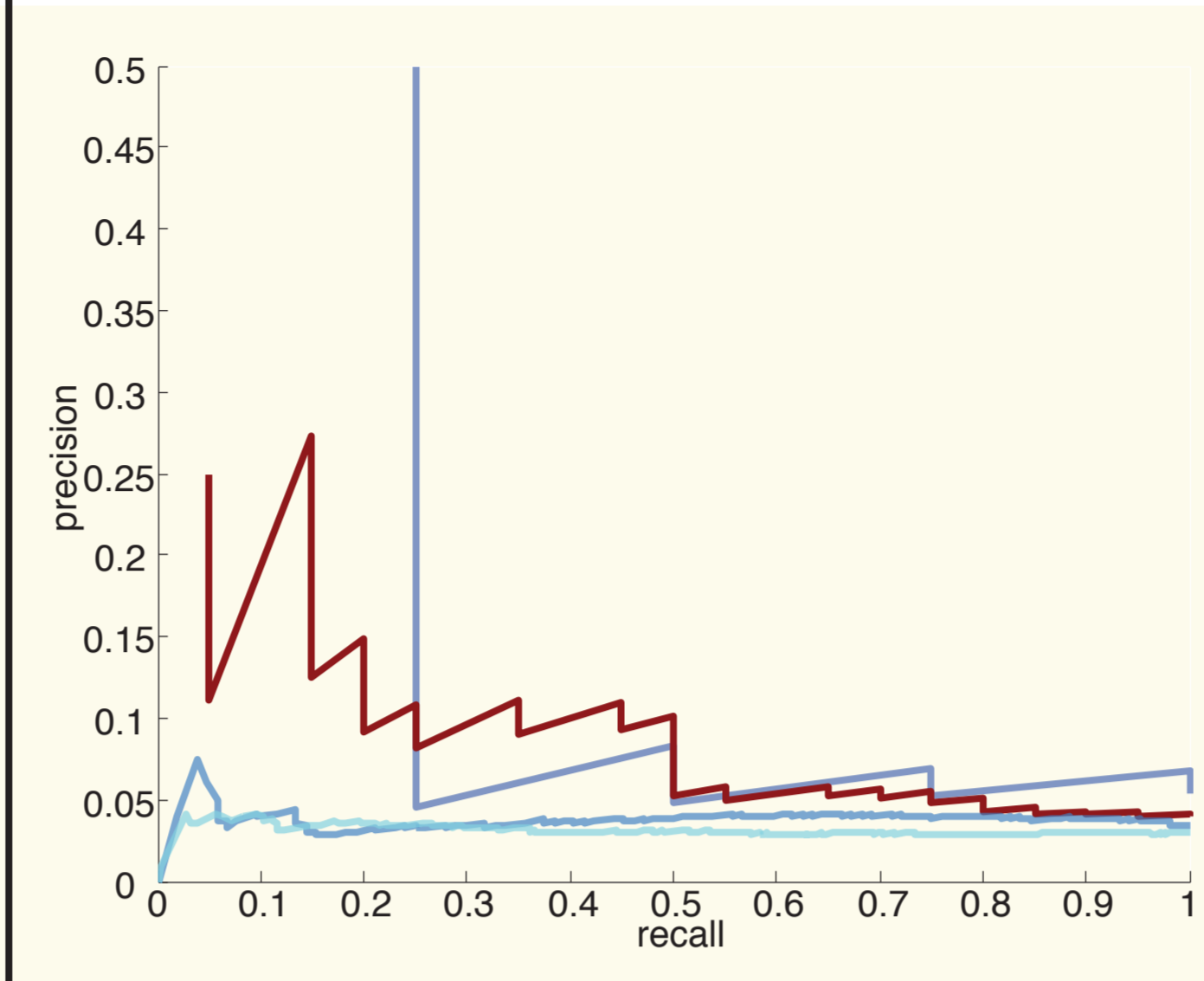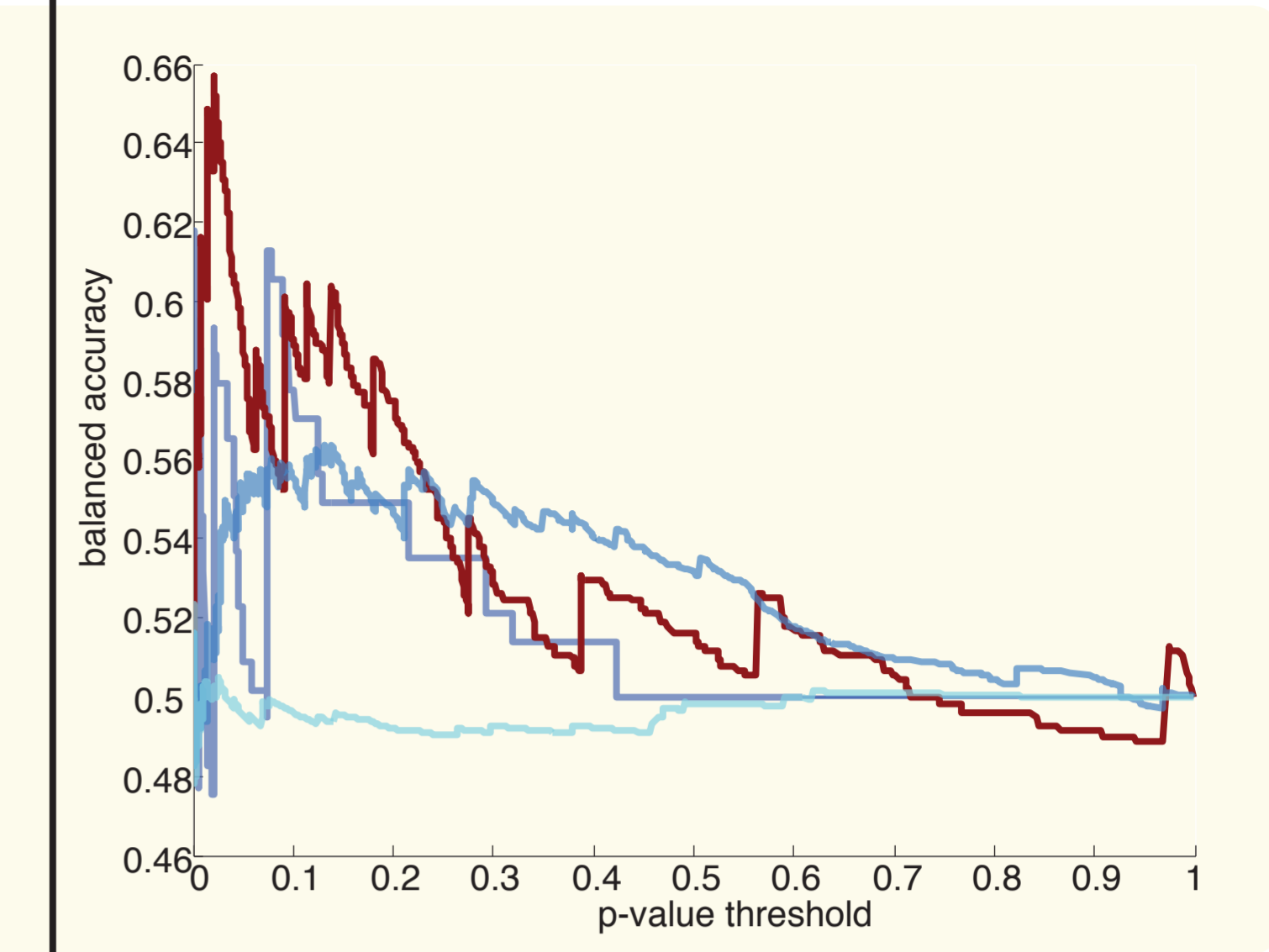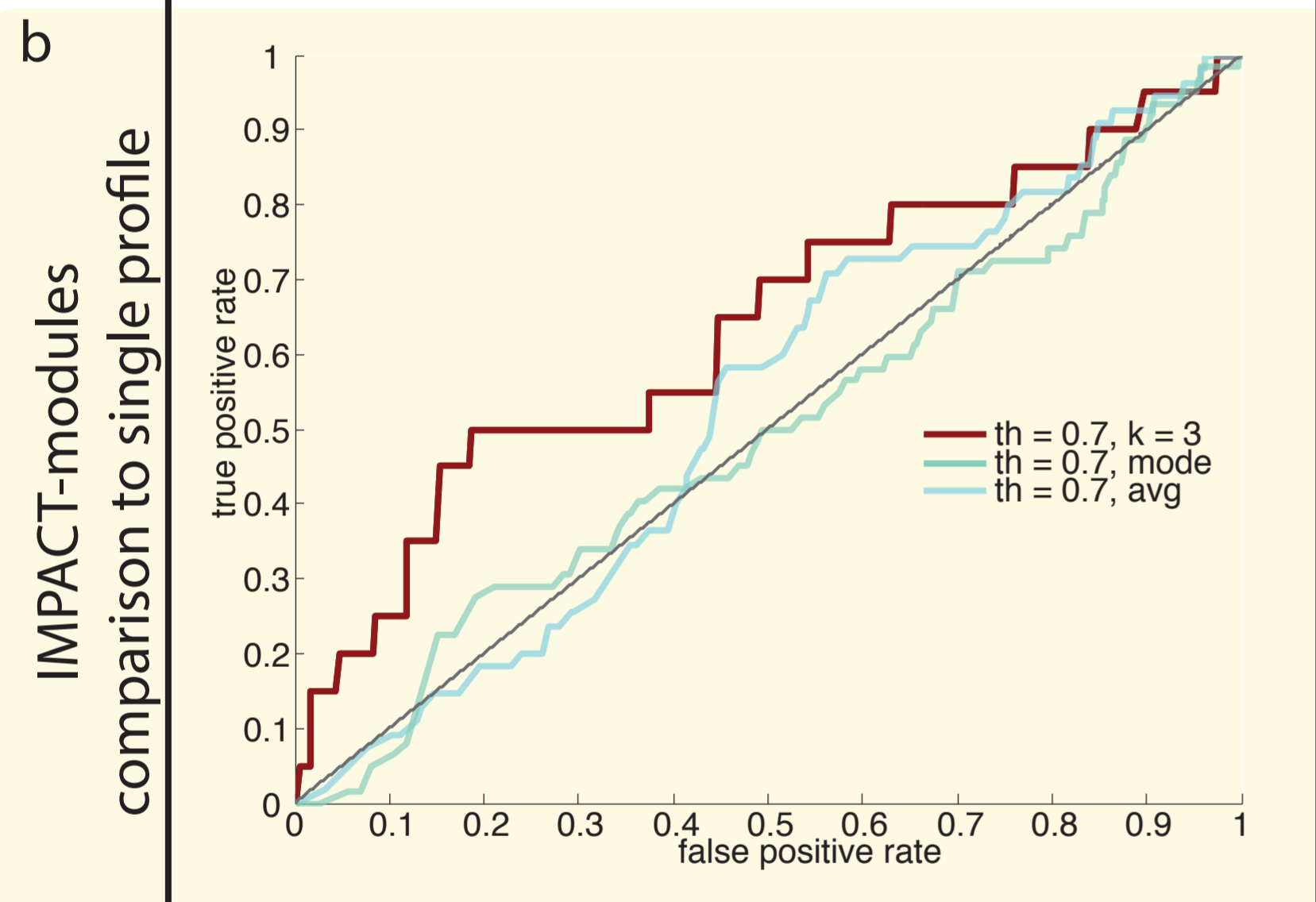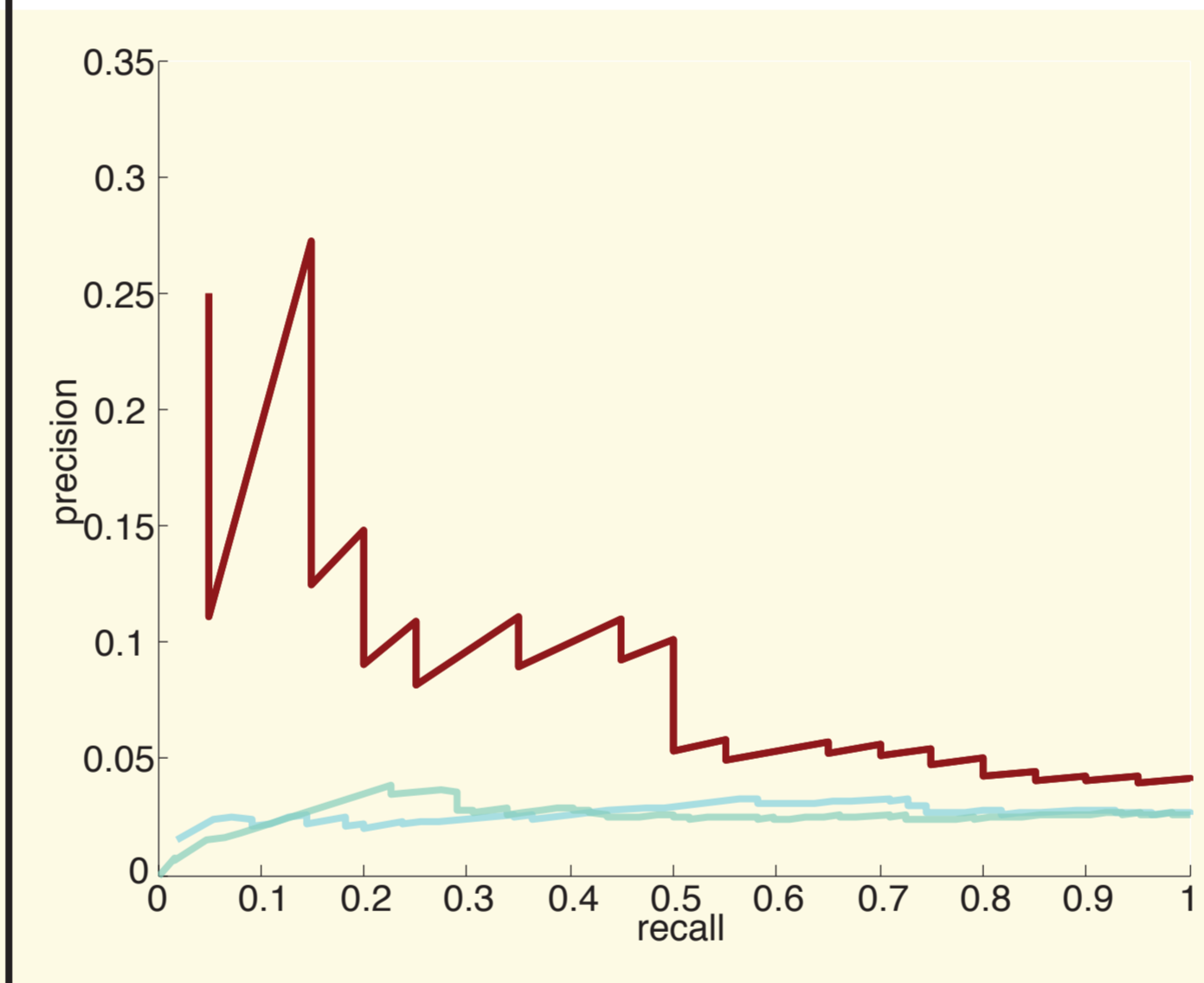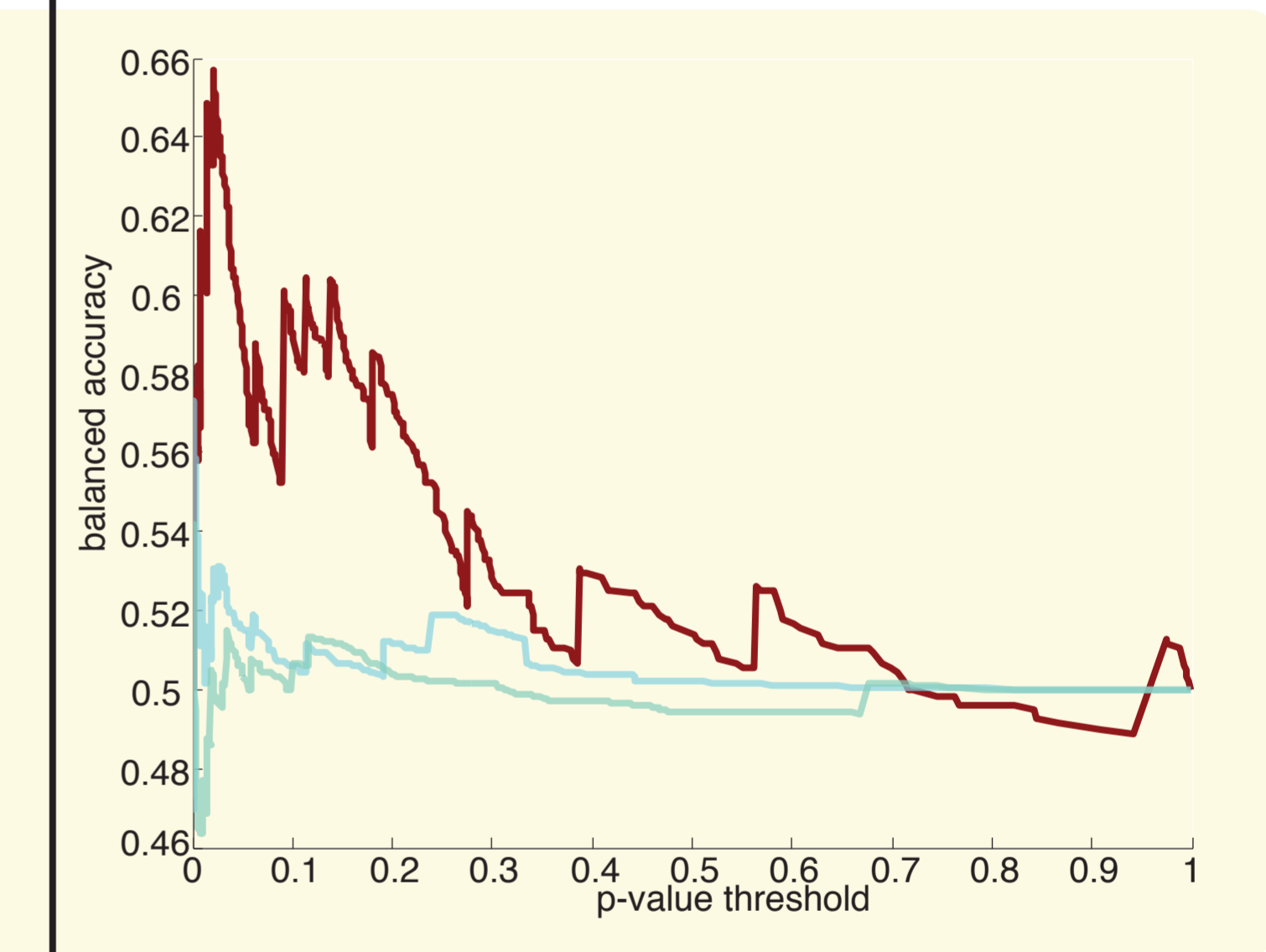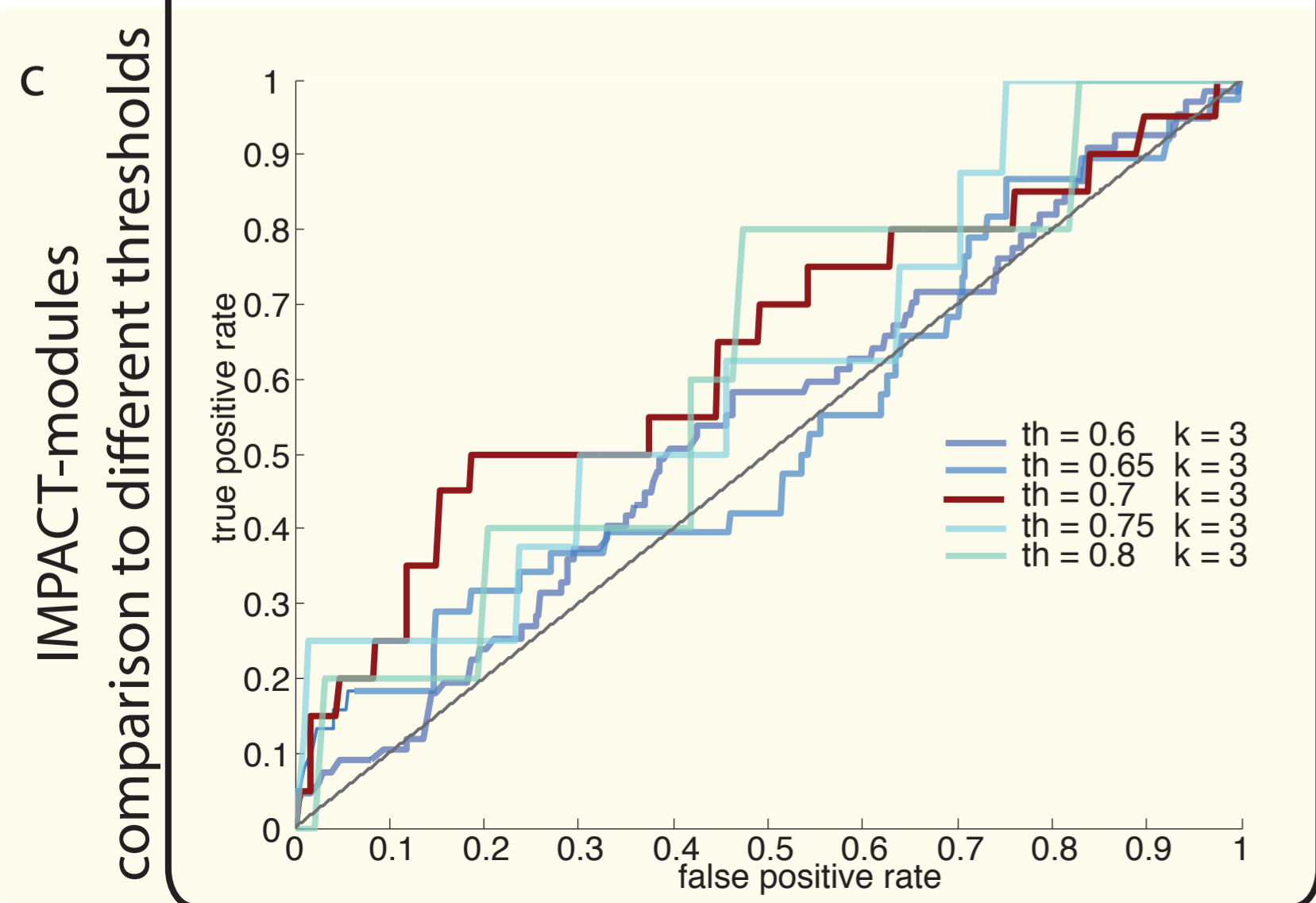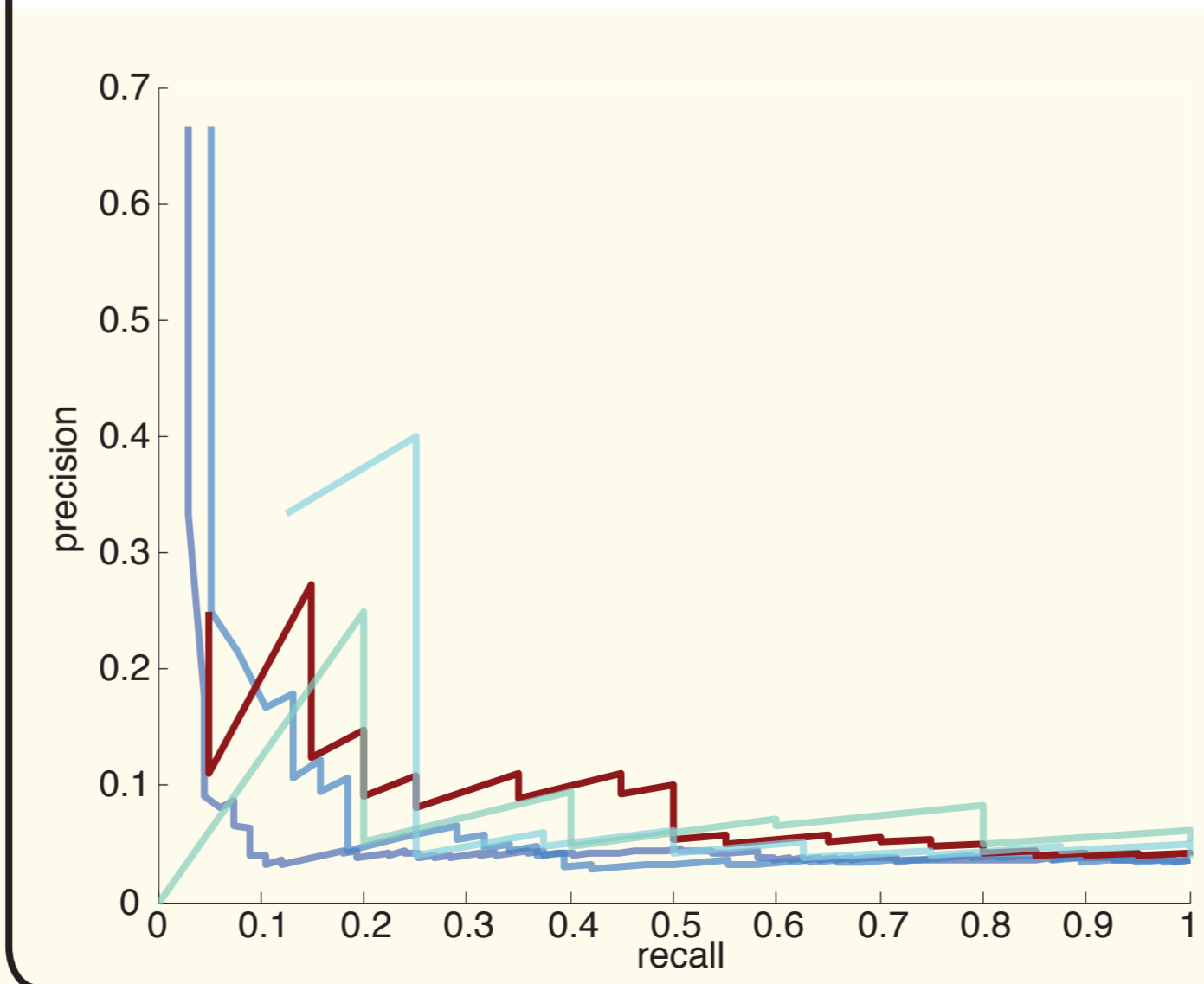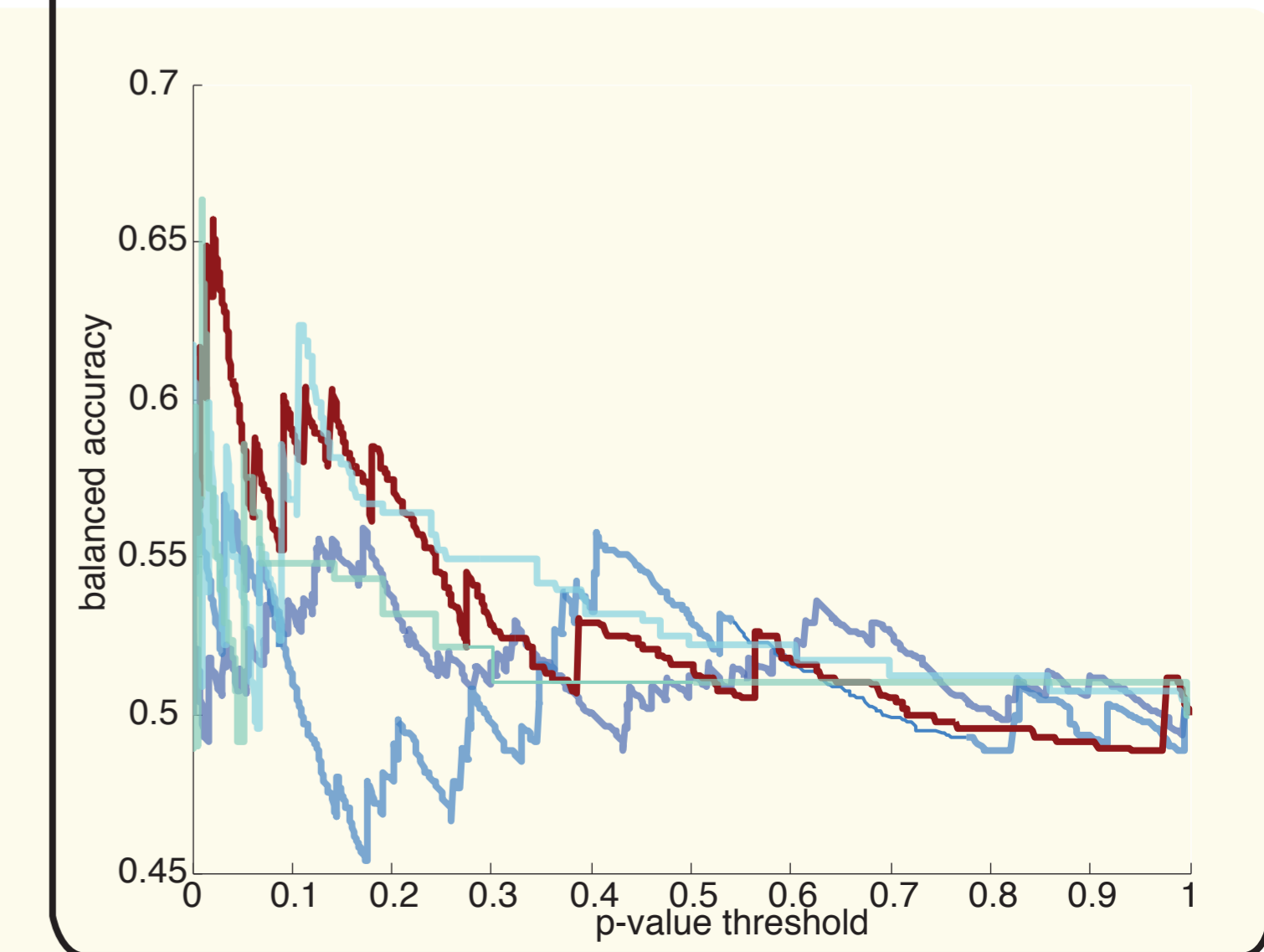

Supplement: Figure S7 — ROC, PR and BACC curves for IMPACT-modules. Row (a): comparison of different minimal number of profiles k. Row (b): comparison of our integrative analysis to the same analysis done using a single profile. Row (c): comparison between different searching thresholds (T). (PDF) [file pcbi.1003801.s007.pdf]

ROC

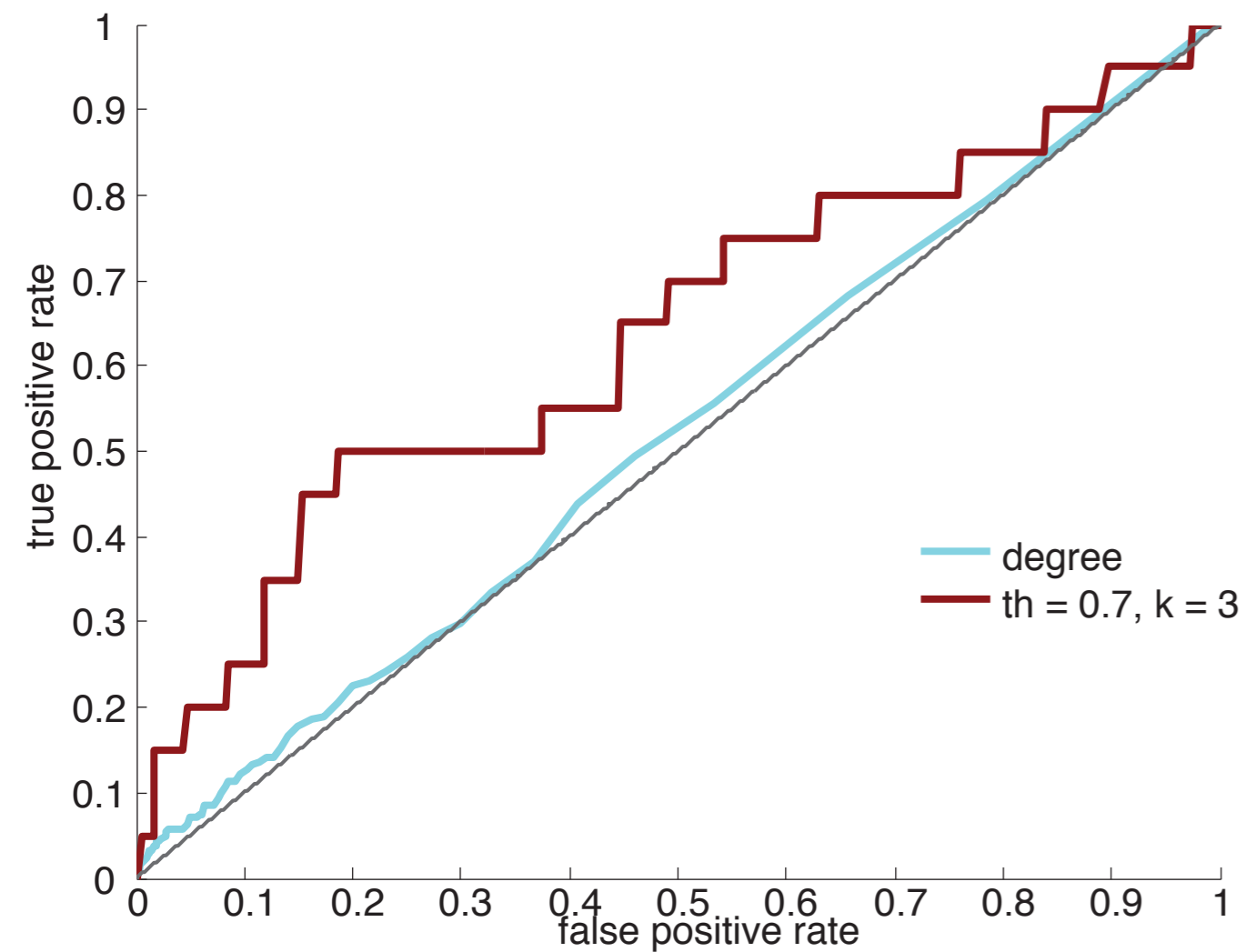

PR

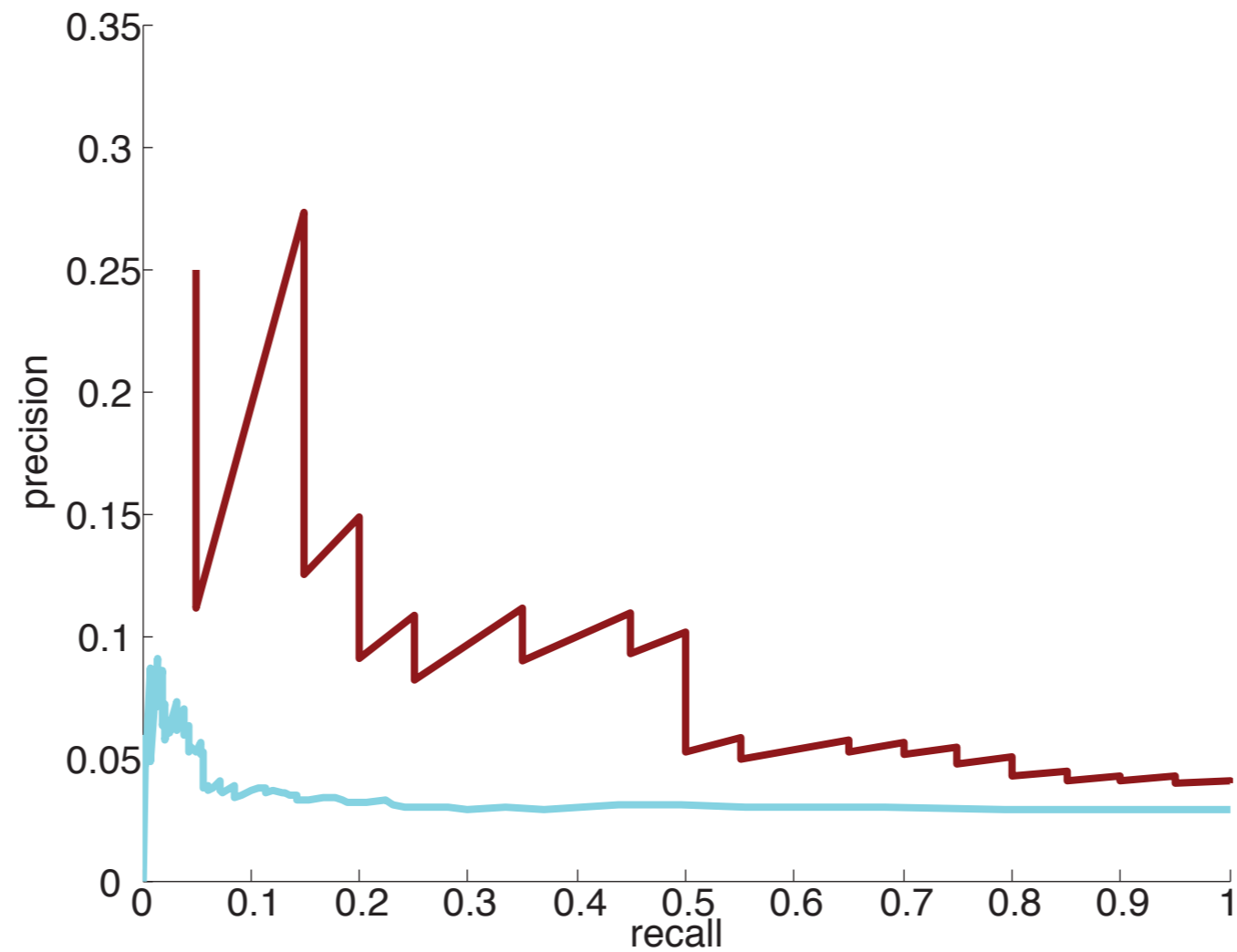

BACC

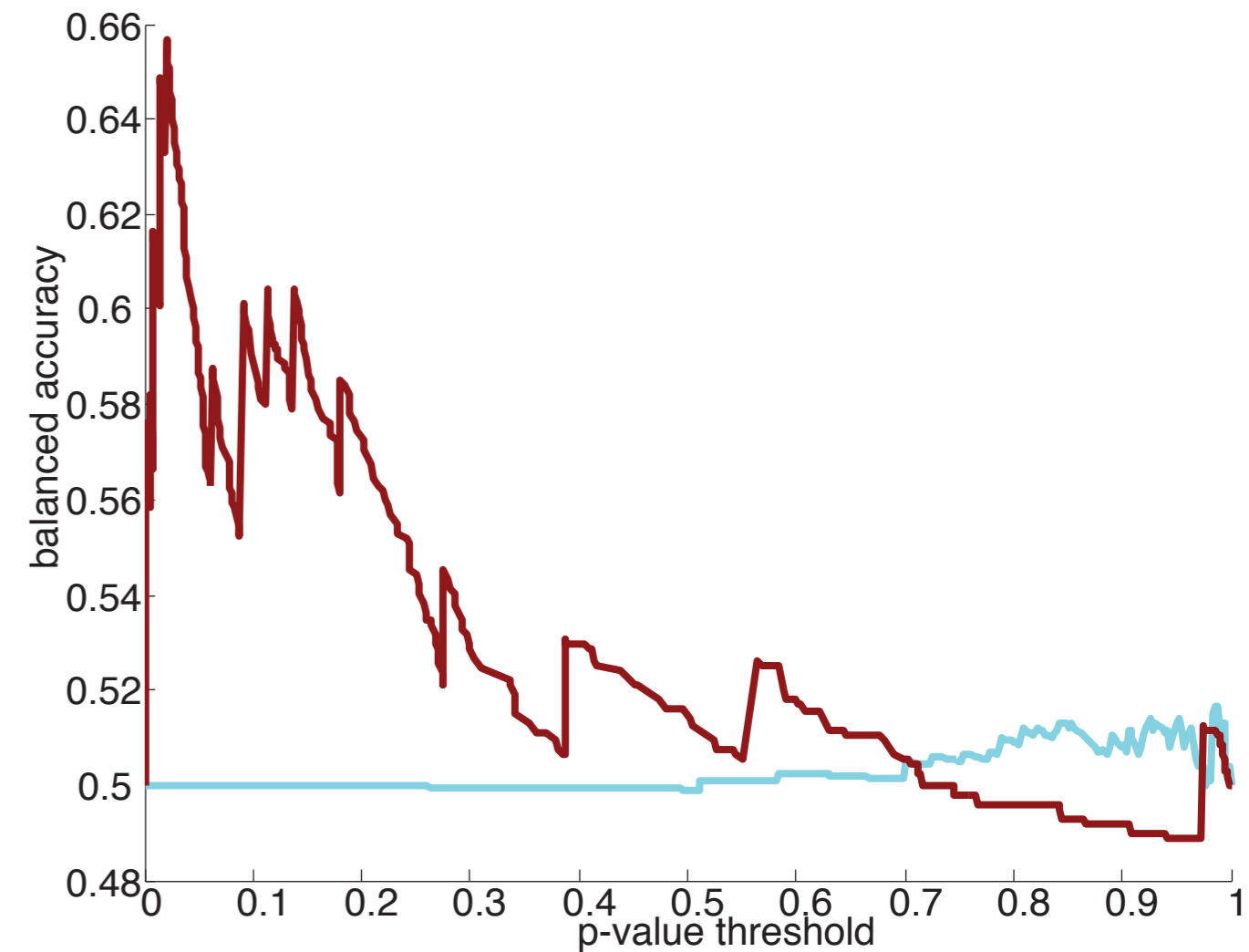

Supplement: Figure S8 — ROC, PR and BACC curves for the gene degree analysis. Comparison of classification performances of the topological information alone e.g. gene degree versus IMPACT-modules (T = 0.7, k = 3). (PDF) [file pcbi.1003801.s008.pdf]

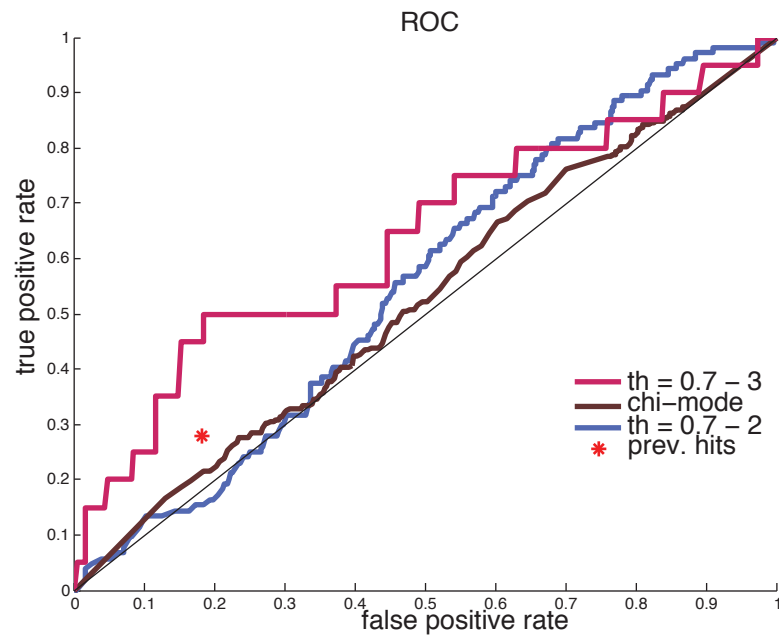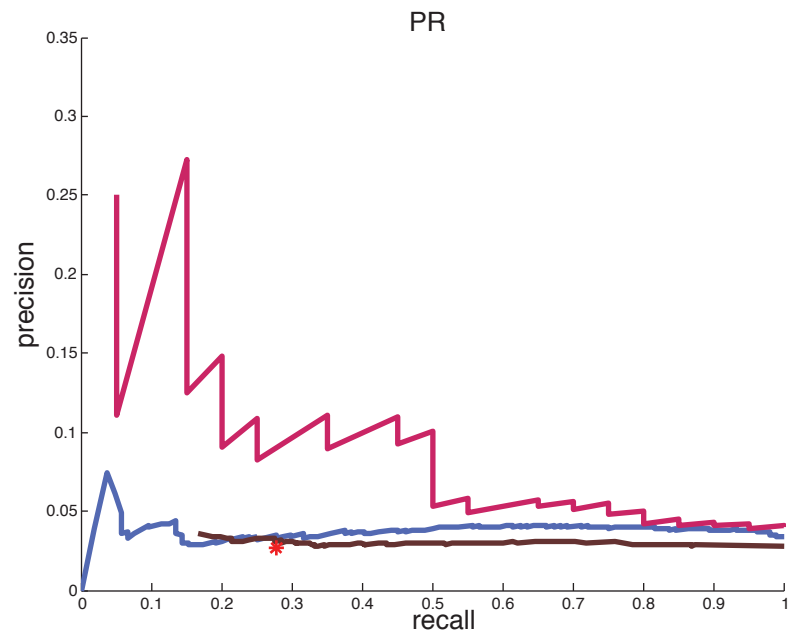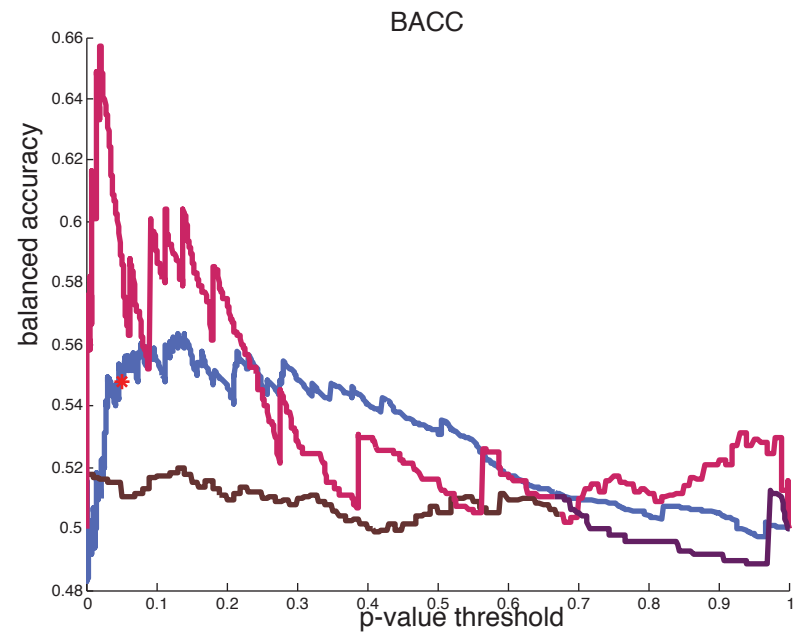

Supplement: Figure S9 — Comparison of IMPACT-modules with the published endocytosis screen analysis. ROC, PR and BACC curves showing an improved detection of endocytosis genes (GO terms, n = 289) for network modules when compared to the previously published analysis based on the chi-square statistics (chi-mode in the legend). The red asterisk (*) reports the performance using the hit-list definition of the previous analysis [1], which was based on a combined evaluation of the phenotypic strength (chi-square) and phenotypic specificity (Phenoscore). The values are calculated by the true and false positive rates based on a significance threshold of 0.05, as in the published hit list. IMPACT can recover a higher number of true positives (ROC curve) at the same false positive rate as the previous analysis. (PDF) [file pcbi.1003801.s009.pdf]

IMPACT

Chi-square  
mode profile

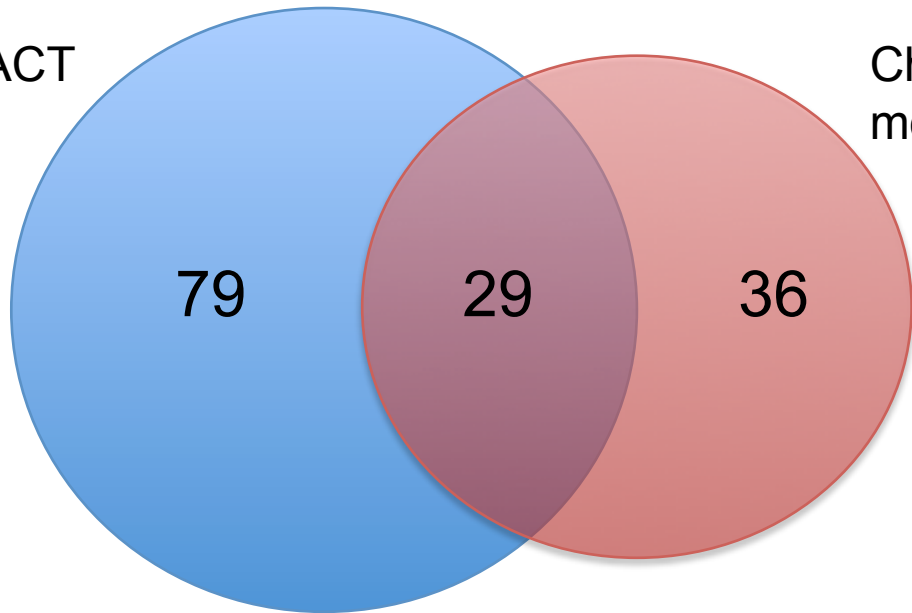

Supplement: Figure S10 — Recovery of known endocytosis genes by IMPACT versus the original publication. Venn diagrams reporting counts of endocytosis genes (based on GO annotation) selected as significant by IMPACT (blue circle) and previously published as screen hits [1] based on the Chi-square of the mode profile (red circle). To perform a balanced comparison, we selected the 2,720 significant (p-value< = 0.1) genes from IMPACT-sets and IMPACT-modules (Table S5) and the top 2,720 genes from the sorted Chi-square list. Out of the 36 genes found in the top Chi-square list and not detected by IMPACT, 26 could be mapped on our interaction data. The higher number of endocytic genes recovered specifically by IMPACT (79) compared to the ones missed (36, of which 26 mapped), shows that it has better sensitivity/specificity trade-off, as also highlighted by the AUC analysis. (PDF) [file pcbi.1003801.s010.pdf]

EGF time course (fitting)

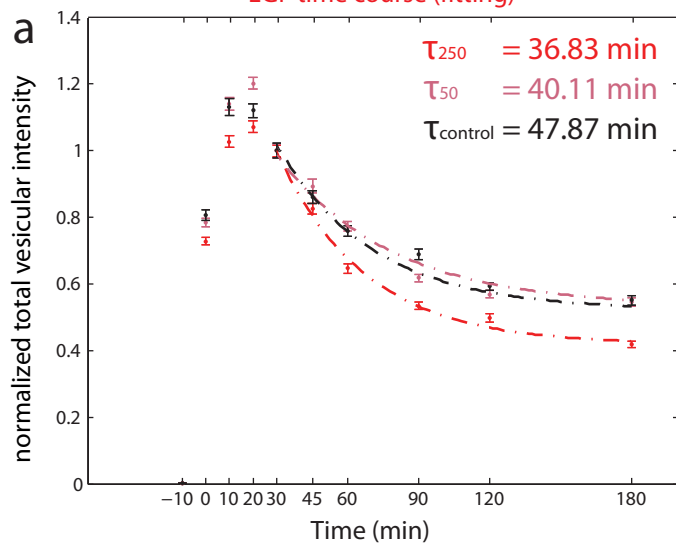

TF time course (fitting)

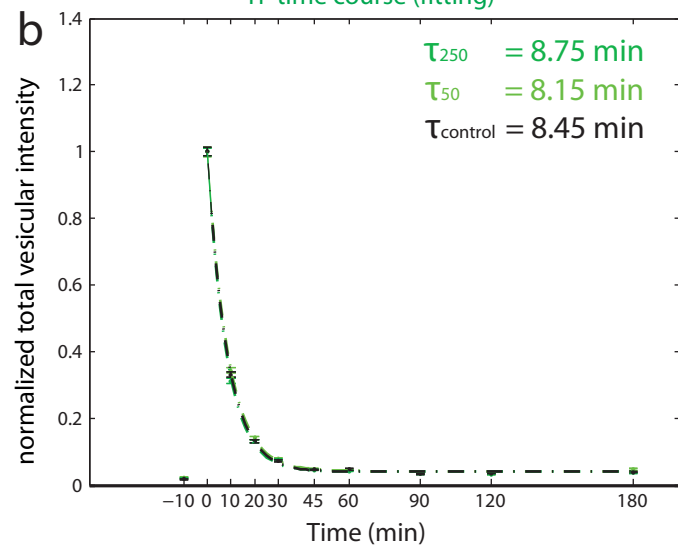

Supplement: Figure S11 — IGF-1 co-stimulation experiments: pulse-chase experiment of labeled EGF and transferrin in presence of IGF-1. (a) Temporal profile of the total vesicular intensity calculated for the EGF-positive endosomes, with different concentration of IGF-1 (dark red: 250 ng/ml; light red: 50 ng/ml; black: no IGF-1 (0 ng/ml), or control), normalized by the time point 30′-chase to better visualize the monotonously decaying phase. (b) Temporal profile of the total vesicular intensity calculated for the TF-positive endosomes, with different concentration of IGF-1 (dark green: 250 ng/ml; light green: 50 ng/ml; black: no IGF-1 (0 ng/ml), or control), normalized by the time point 0′-chase to better visualize the monotonously decaying phase. Normalized experimental points (dots plus error bars) and the fitted curves (dashed lines), obtained by fitting the decaying exponential function f(x) = A*e−t/τ, are shown in both panels (a) and (b). The insets display the estimated time constants of the respective fitting. (PDF) [file pcbi.1003801.s011.pdf]

a)

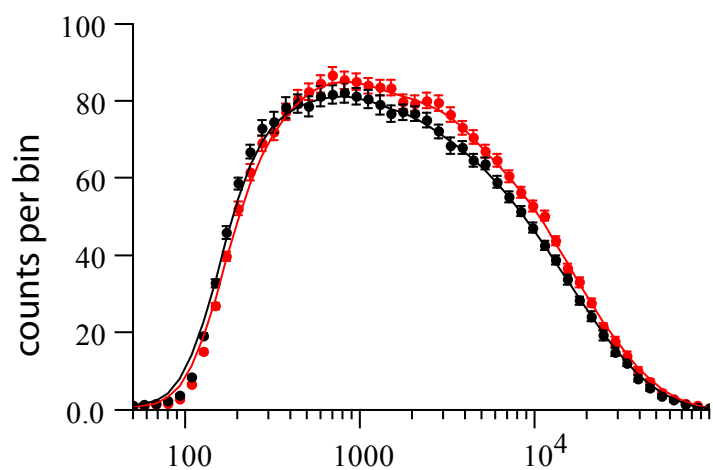

b)

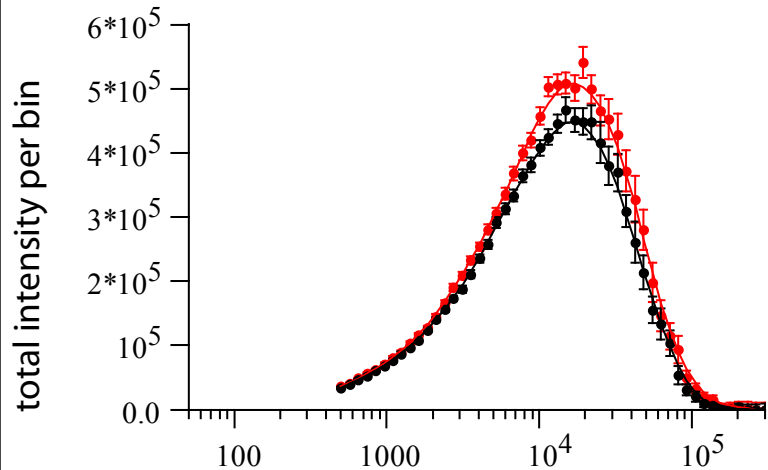

c)

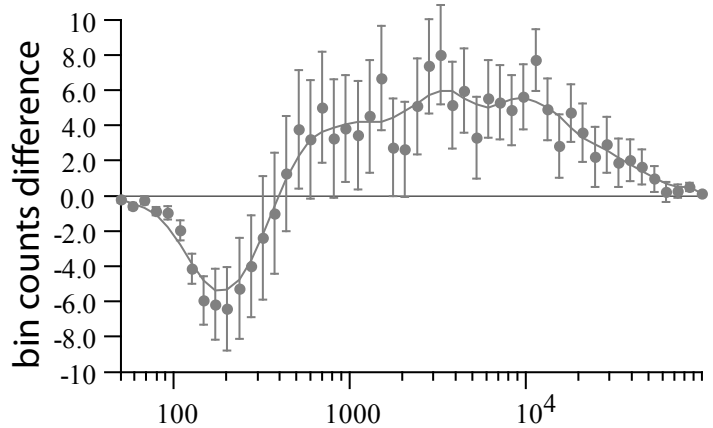

d)

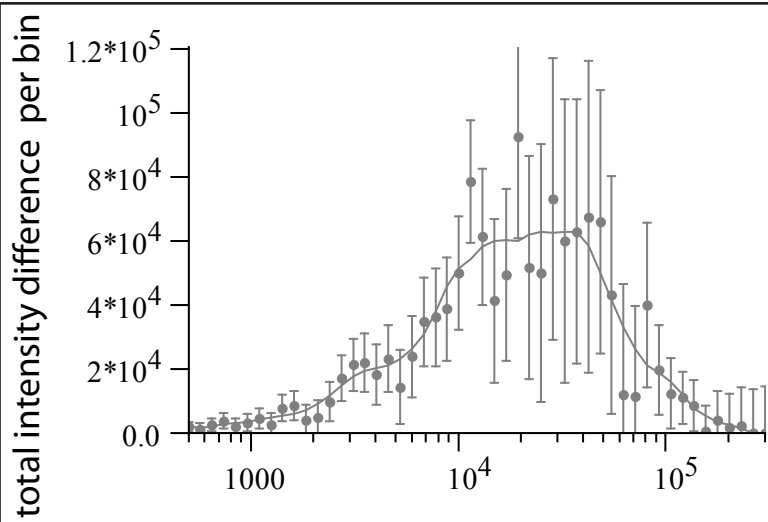

Supplement: Figure S12 — Distribution analysis for the IGF-1 co-stimulation experiment. (a) The integral intensity (i.e., the integral of the intensity of the fitted object, representing per-endosome cargo content) is calculated per-each endosome. From here, a histogram is built showing how many endosomes are counted (y-axis) for each bin of mean vesicular integral intensity (x-axis). The two curves (black and red) represent two arbitrary conditions to be compared. (b) Starting from the mean integral intensity distribution shown in panel (a), it is possible to calculate the total vesicular intensity distribution shown here, by multiplying each bin of mean integral intensity (x-value) by the number of counts in that bin. This distribution displays how much cargo is contained in different sub-population of endosomes, that contain different mean cargo amount. (c) Bin count difference of the two distributions shown in panel (a): it is here visible that low cargo containing endosomes are depleted in the “red” condition, whereas bigger endosomes containing more cargo are enriched. The continuous grey line represents smoothing by moving average. (d) Distribution of the total vesicular intensity difference per bin, calculated subtracting the two curves in panel (b): it is here visible that endosomes from the “red” condition contain more cargo than the ones from the “black” condition, especially for high bins (>104) of mean integral intensity (i.e., big endosomes containing a lot of cargo). The continuous grey line represents smoothing by moving average. The visualization depicted in panel (d) is what has been used in Figures 6. (PDF) [file pcbi.1003801.s012.pdf]

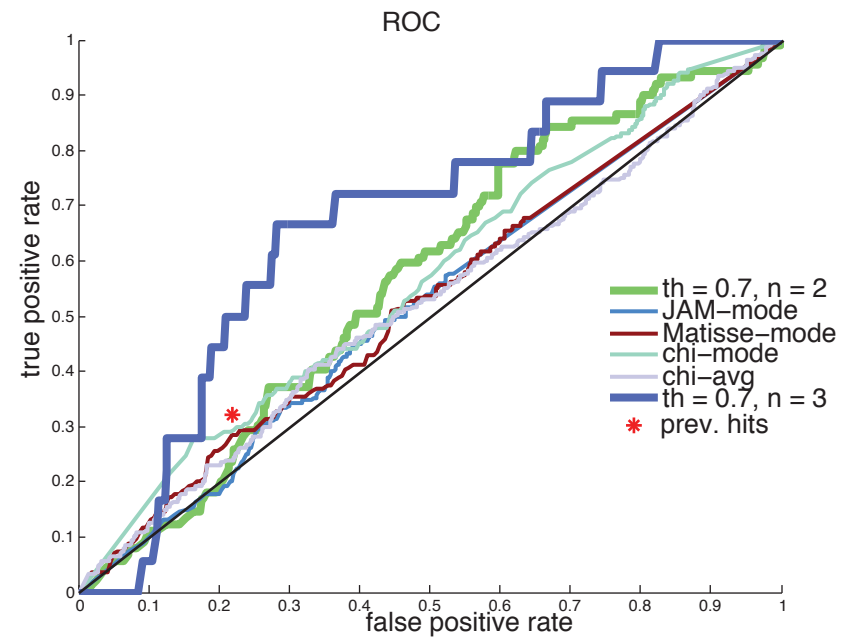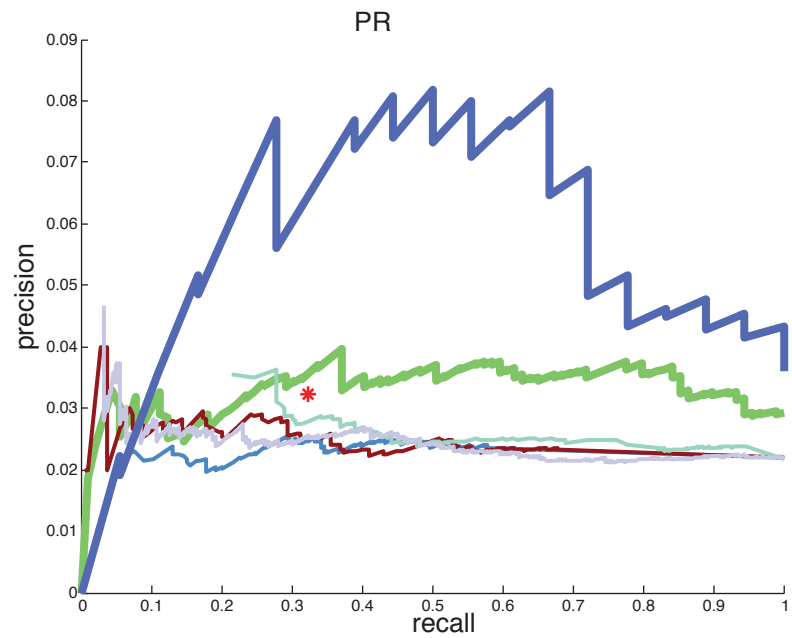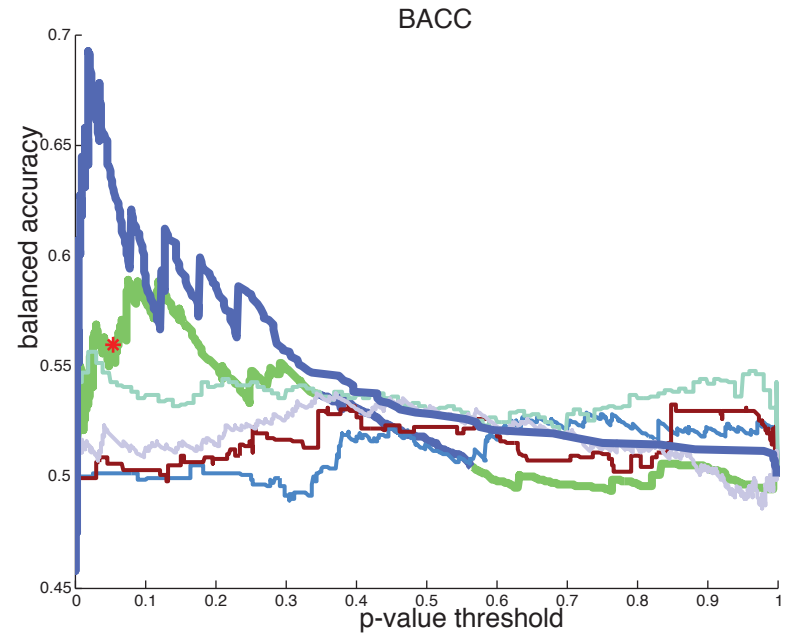

Supplement: Figure S15 — Comparison of IMPACT-modules with other methods on an independent list of genes. ROC, PR and BACC curves for the direct comparison of IMPACT-modules with other methods in classifying an independent list not used for parameter tuning, to account for potential overfitting. We used the merged list of Rab5 effectors [2] and of proteins with endocytic domains related to endocytosis (PX, FYVE, BAR, TBD and VPS9, [1]), comprising of 306 members, of which 213 are present in the interaction network (the overlap with the endocytosis GO annotation gene list was just 62 genes). IMPACT-modules (both for T = 0.7, k = 3 and T = 0.7, k = 2) out-performed the other approaches on this new list (shown here) as well as on the union of the two, the GO terms list and the RAB5 effectors and domains list (not shown). The red asterisk (*) in all three panels indicates single values relative to the hit list definition of the previous analysis [1], which was based on a combined evaluation of the phenotypic strength (chi-square) and phenotypic specificity (Phenoscore). The values are calculated by the true and false positive rates based on a significance threshold of 0.05, as in the published hit list. At equal false positive rate value as the hits list from the previous analysis, IMPACT can recover a higher number of true positives (ROC curve). (PDF) [file pcbi.1003801.s015.pdf]

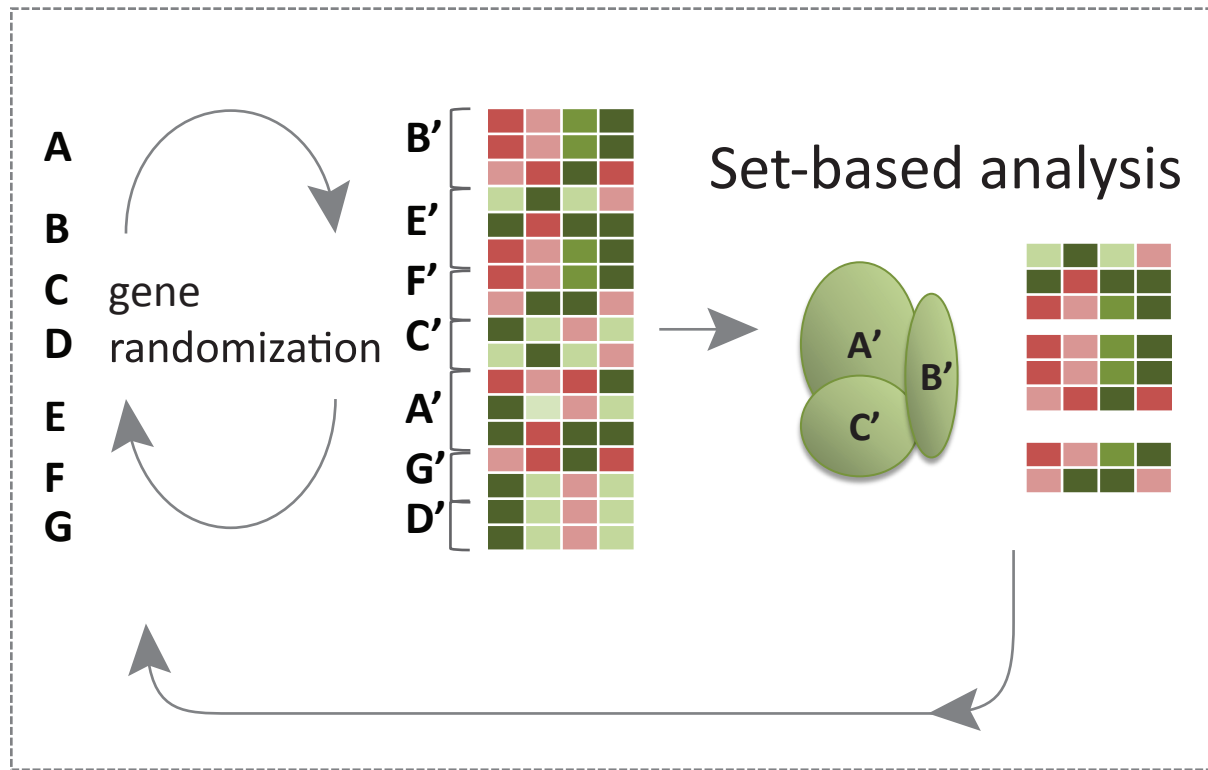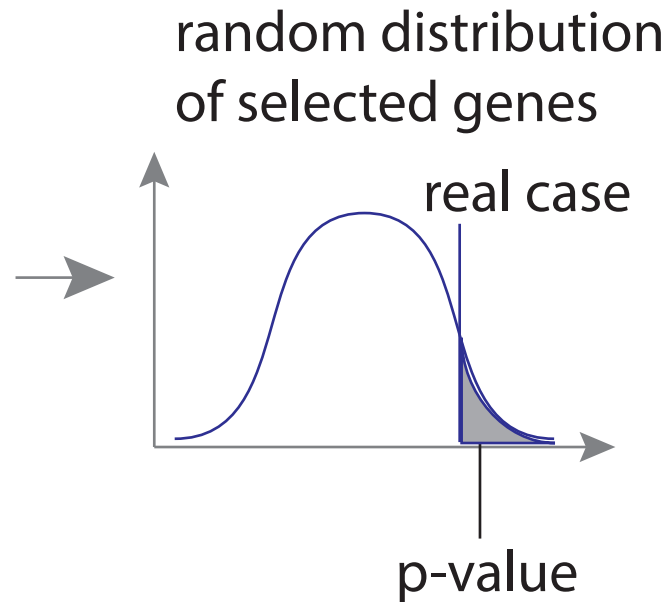

Supplement: Figure S16 — Assessment of statistical significance for the set-based analysis. For the set-based analysis, significance is determined through appropriate randomizations that take into account the genes and profiles number in each complex: 1) gene labels are permuted across the entire dataset keeping together profiles belonging to the same gene; 2) complexes subunits are reshuffled only with random subunits having a comparable number of profiles. (PDF) [file pcbi.1003801.s016.pdf]

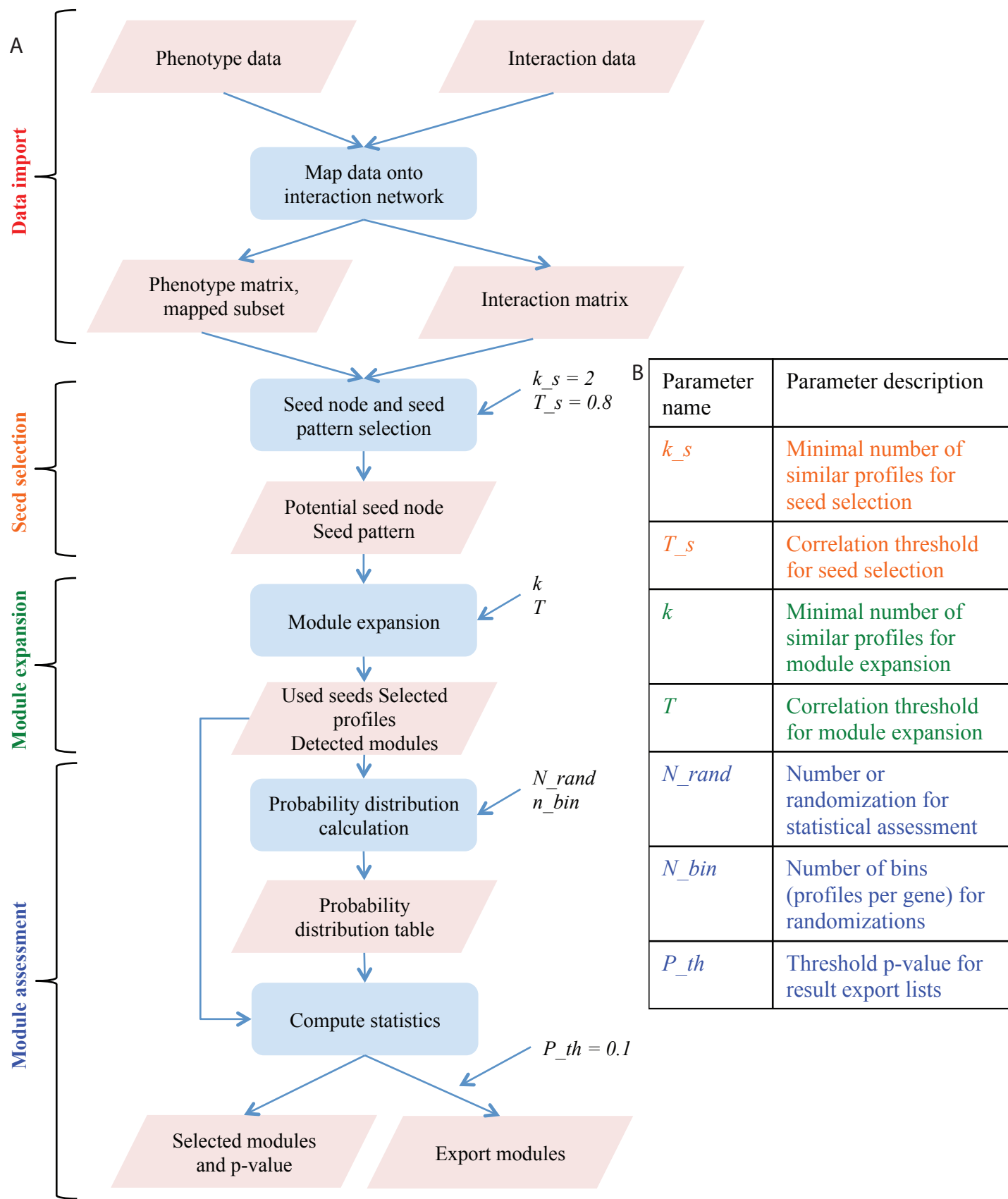

Supplement: Figure S17 — Visual description of IMPACT-modules pipeline. A) Flowchart of the network-based approach. Parallelograms Indicate input/output steps, rectangles describe processing steps. Different sections described in the main text (Methods) are indicated on the left. The pipeline takes two input files, the phenotype data and the prior information (interaction data), see Input Data in Methods. B) Description for the parameters reported on the right of the flowchart. A similar flowchart applies to the IMPACT-set module, with the only following difference that there is no seed selection step, as each set (i.e. protein complex) is considered for the analysis. (PDF) [file pcbi.1003801.s017.pdf]

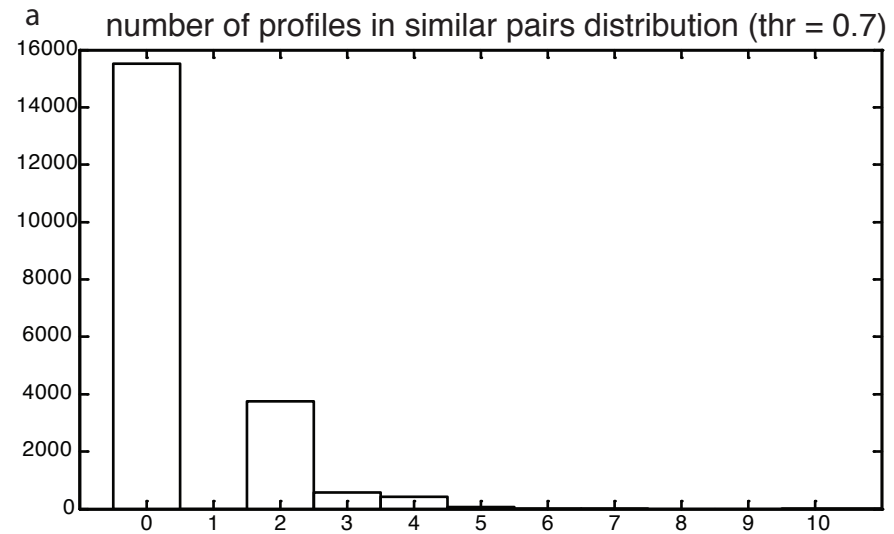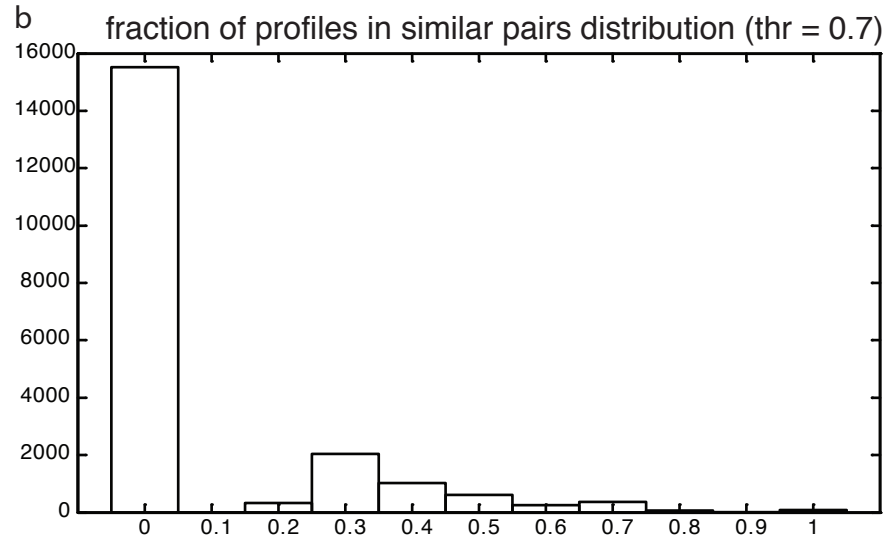

Supplement: Figure S18 — Distribution of pairwise similarity for oligonucleotides of the same gene. a) Distribution of the number of profiles similar above T = 0.7 within the same gene (n_s). b) Distribution for the fraction of profiles similar above threshold T = 0.7 within the same gene, calculated as n_s/n_tot, with n_tot the total number of profiles per gene. (PDF) [file pcbi.1003801.s018.pdf]
