# Supplementary material for: Revealing Molecular Mechanisms by Integrating High-Dimensional Functional Screens with Protein Interaction Data
Source: PLoS Comput Biol. 2014 Sep 4;10(9):e1003801. doi: 10.1371/journal.pcbi.1003801 (PMC4154648; doi:10.1371/journal.pcbi.1003801)
Supplement: Figure S6 — ROC, PR and BACC curves for IMPACT-sets. Row (a): comparison of different searching thresholds. Row (b): comparison IMPACT-sets to the analysis done using a single profile (average and mode of the original oligo profiles). (PDF) [file pcbi.1003801.s006.pdf]

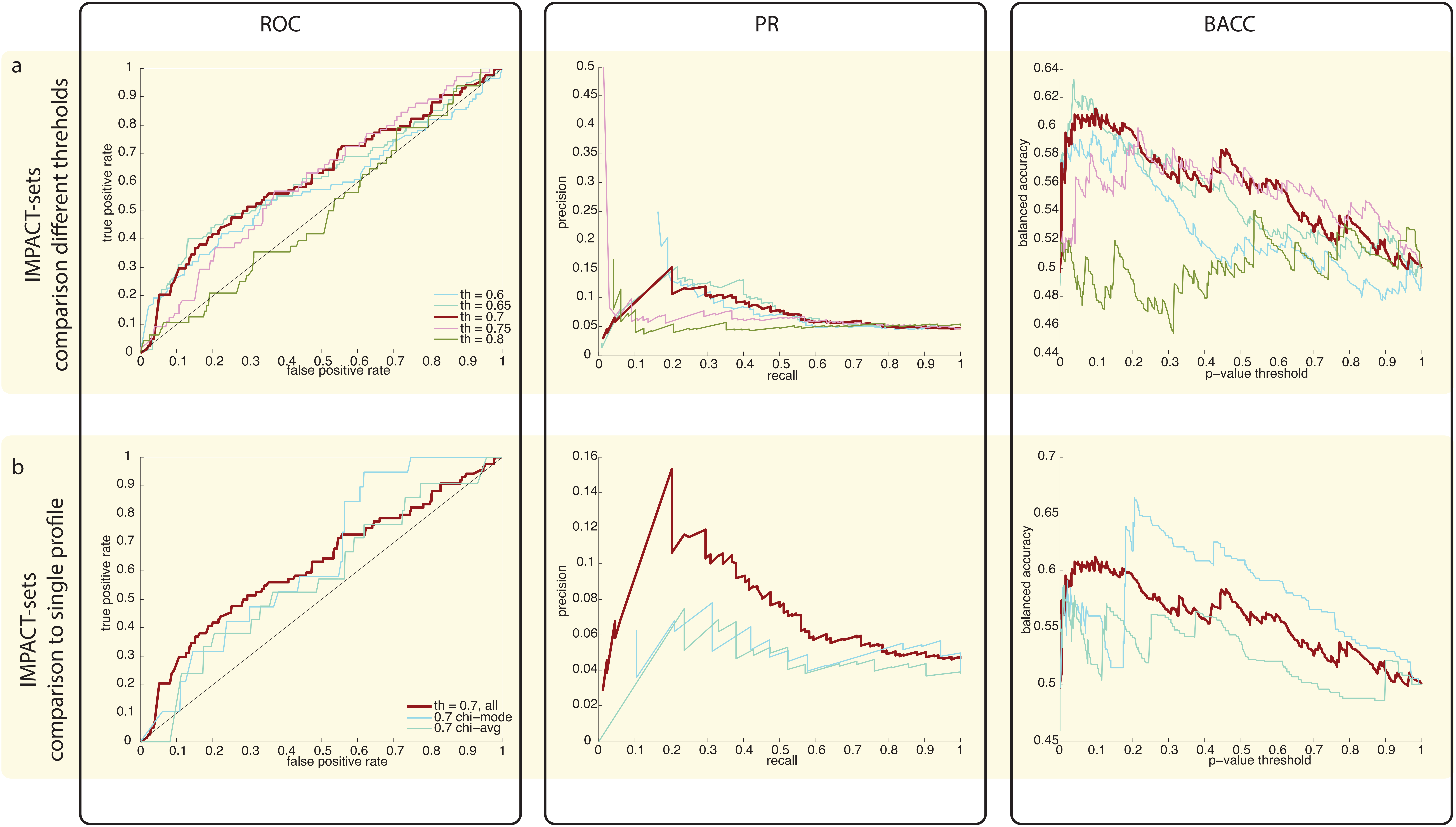

ROC
PR
BACC
a
IMPACT-sets
comparison different thresholds
true positive rate
false positive rate
th = 0.6
th = 0.65
th = 0.7
th = 0.75
th = 0.8
precision
recall
balanced accuracy
p-value threshold
b
IMPACT-sets
comparison to single profile
th = 0.7, all
0.7 chi–mode
0.7 chi–avg
