# Supplementary material for: Revealing Molecular Mechanisms by Integrating High-Dimensional Functional Screens with Protein Interaction Data
Source: PLoS Comput Biol. 2014 Sep 4;10(9):e1003801. doi: 10.1371/journal.pcbi.1003801 (PMC4154648; doi:10.1371/journal.pcbi.1003801)
Supplement: Figure S13 — Details of the phenotypic space figure for complexes. Proteins being part of the complexes are shown in the respective insets. Asterisks (*) indicate genes having at least one profile correlated with the profile shown in the insets in Figure 4. (PDF) [file pcbi.1003801.s013.pdf]

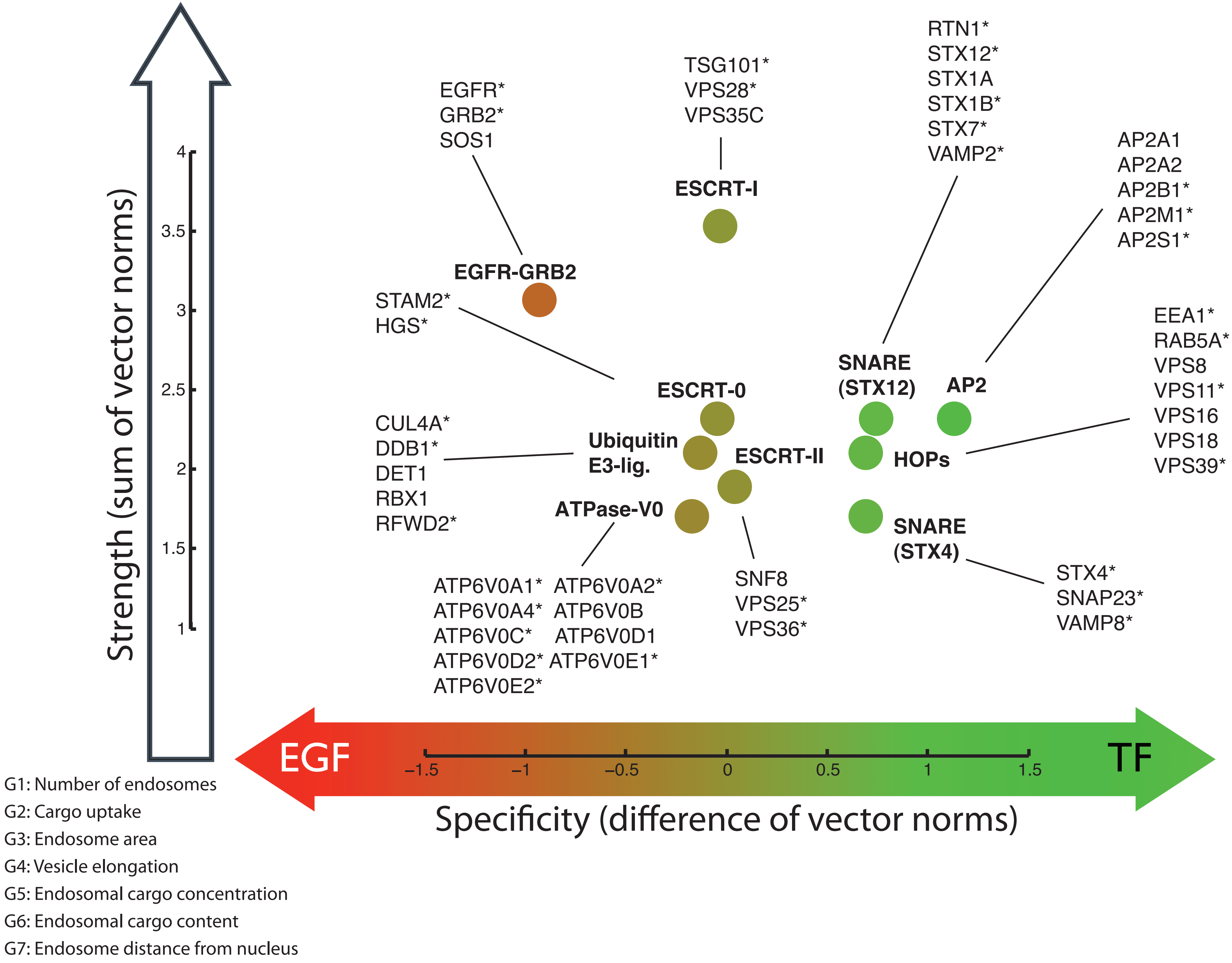

Strength (sum of vector norms)
4
3.5
3
2.5
2
1.5
1
EGFR*
GRB2*
SOS1
EGFR-GRB2
TSG101*
VPS28*
VPS35C
ESCRT-I
RTN1*
STX12*
STX1A
STX1B*
STX7*
VAMP2*
AP2A1
AP2A2
AP2B1*
AP2M1*
AP2S1*
STAM2*
HGS*
ESCRT-0
SNARE (STX12)
AP2
EEA1*
RAB5A*
VPS8
VPS11*
VPS16
VPS18
VPS39*
CUL4A*
DDB1*
DET1
RBX1
RFWD2*
Ubiquitin E3-lig.
ESCRT-II
HOPs
ATPase-V0
SNARE (STX4)
ATP6V0A1* ATP6V0A2*
ATP6V0A4* ATP6V0B
ATP6V0C* ATP6V0D1
ATP6V0D2* ATP6V0E1*
ATP6V0E2*
SNF8
VPS25*
VPS36*
STX4*
SNAP23*
VAMP8*
EGF
–1.5
–1
–0.5
0
0.5
1
1.5
TF
Specificity (difference of vector norms)
G1: Number of endosomes
G2: Cargo uptake
G3: Endosome area
G4: Vesicle elongation
G5: Endosomal cargo concentration
G6: Endosomal cargo content
G7: Endosome distance from nucleus
