# Supplementary material for: Revealing Molecular Mechanisms by Integrating High-Dimensional Functional Screens with Protein Interaction Data
Source: PLoS Comput Biol. 2014 Sep 4;10(9):e1003801. doi: 10.1371/journal.pcbi.1003801 (PMC4154648; doi:10.1371/journal.pcbi.1003801)
Supplement: Figure S14 — Details of the phenotypic space figure for modules. Here we report the graphic representations of the modules of Figure 5. We used Cytoscape for the module visualization. (PDF) [file pcbi.1003801.s014.pdf]

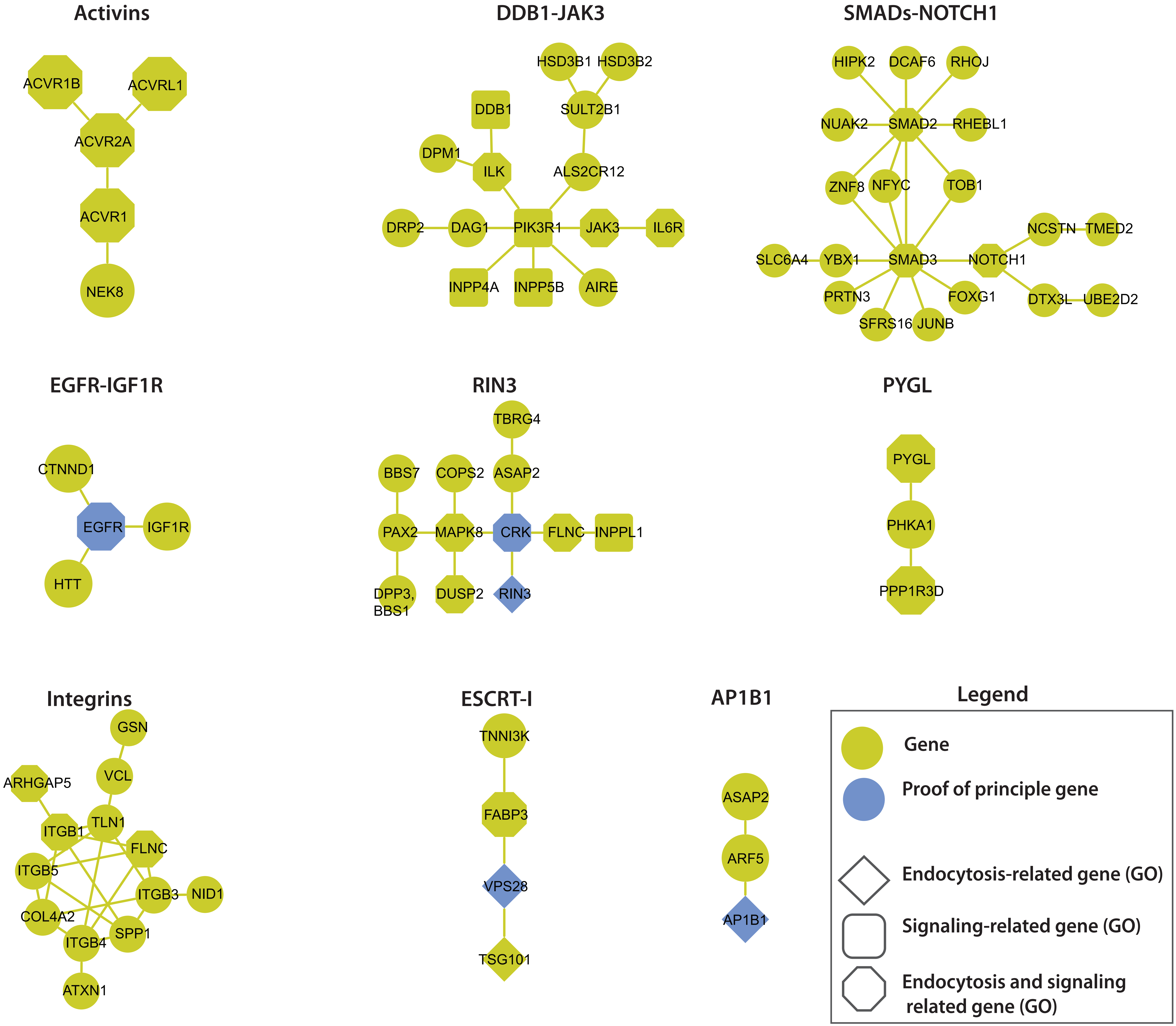
Activins
ACVR1B
ACVRL1
ACVR2A
ACVR1
NEK8
DDB1-JAK3
HSD3B1
HSD3B2
DDB1
SULT2B1
DPM1
ILK
ALS2CR12
DRP2
DAG1
PIK3R1
JAK3
IL6R
INPP4A
INPP5B
AIRE
SMADs-NOTCH1
HIPK2
DCAF6
RHOJ
NUAK2
SMAD2
RHEBL1
ZNF8
NFYC
TOB1
NCSTN
TMED2
SLC6A4
YBX1
SMAD3
NOTCH1
PRTN3
FOXG1
DTX3L
UBE2D2
SFRS16
JUNB
EGFR-IGF1R
CTNND1
EGFR
IGF1R
HTT
RIN3
TBRG4
BBS7
COPS2
ASAP2
PAX2
MAPK8
CRK
FLNC
INPPL1
DPP3,
BBS1
DUSP2
RIN3
PYGL
PYGL
PHKA1
PPP1R3D
Integrins
GSN
VCL
ARHGAP5
ITGB1
TLN1
FLNC
ITGB5
ITGB3
NID1
COL4A2
SPP1
ITGB4
ATXN1
ESCRT-I
TNNI3K
FABP3
VPS28
TSG101
AP1B1
ASAP2
ARF5
AP1B1
Legend
Gene
Proof of principle gene
Endocytosis-related gene (GO)
Signaling-related gene (GO)
Endocytosis and signaling
related gene (GO)
