# Supplementary material for: Revealing Molecular Mechanisms by Integrating High-Dimensional Functional Screens with Protein Interaction Data
Source: PLoS Comput Biol. 2014 Sep 4;10(9):e1003801. doi: 10.1371/journal.pcbi.1003801 (PMC4154648; doi:10.1371/journal.pcbi.1003801)
Supplement: Figure S19 — Reference profiles better match re-screen profiles than original phenotypic profiles. Profiles selected by IMPACT (blue curves) compared to all the oligonucleotide profiles in the old screen data (left) and the new rescreen data (right). Three examples are shown (top to bottom): PDPK1, MLC1, IGF1R. X-axes: parameter index as described in Table S1. Y-axes: normalized parameter value. For further description of plots see Figure S1. (PDF) [file pcbi.1003801.s019.pdf]

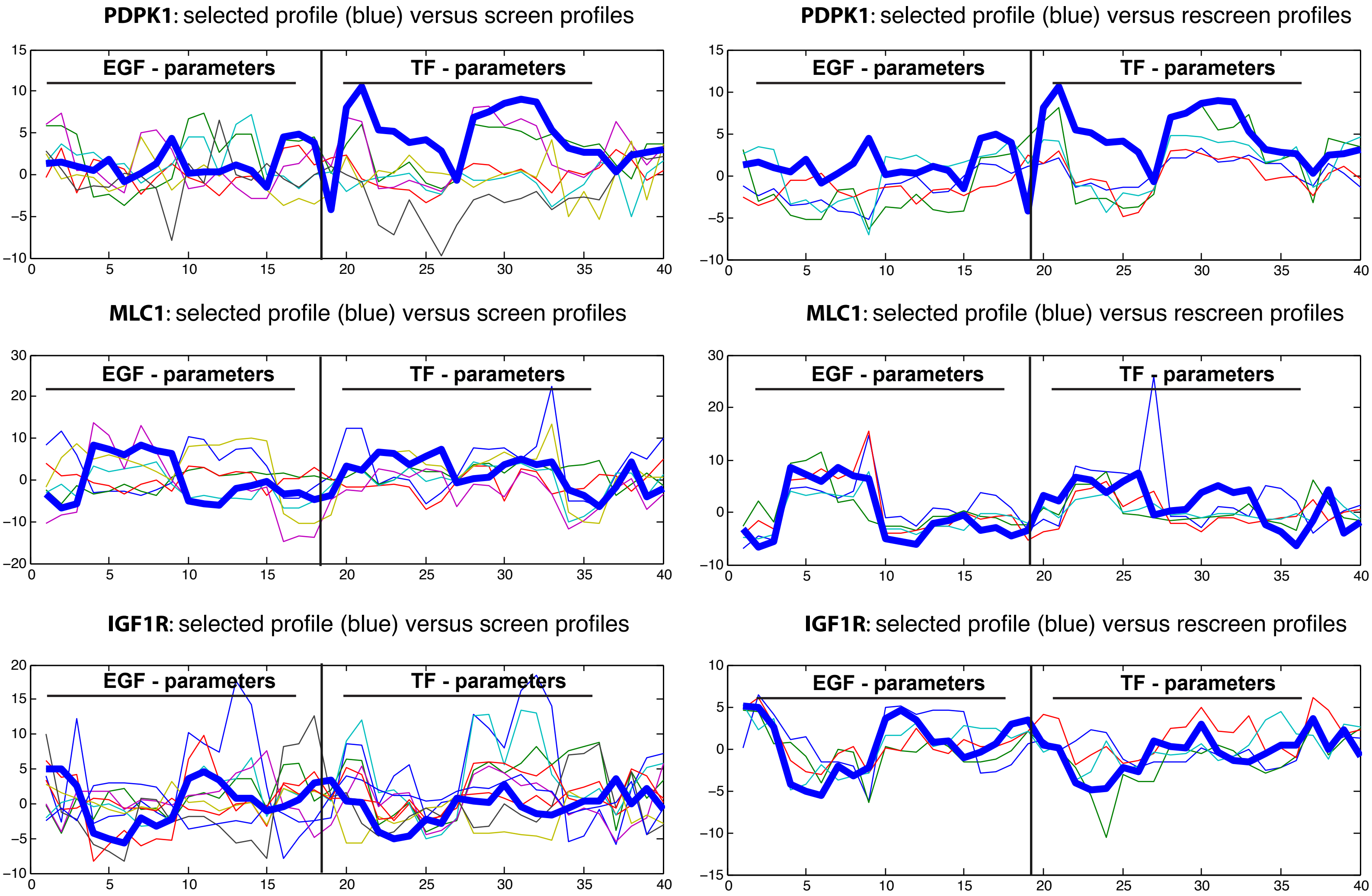

PDPK1: selected profile (blue) versus screen profiles
PDPK1: selected profile (blue) versus rescreen profiles
MLC1: selected profile (blue) versus screen profiles
MLC1: selected profile (blue) versus rescreen profiles
IGF1R: selected profile (blue) versus screen profiles
IGF1R: selected profile (blue) versus rescreen profiles
EGF - parameters
TF - parameters
