# Supplementary material for: Revealing Molecular Mechanisms by Integrating High-Dimensional Functional Screens with Protein Interaction Data
Source: PLoS Comput Biol. 2014 Sep 4;10(9):e1003801. doi: 10.1371/journal.pcbi.1003801 (PMC4154648; doi:10.1371/journal.pcbi.1003801)
Supplement: Table S1 — List of parameters used in the RNAi screen assay [1] (prev. page). The first two columns describe respectively the label and the description for the parameter groups used as summarized graphical representation (Figures 4 and 5). The third column enumerates all the parameters constituting each group. The numbers in parentheses indicate the parameter index for EGF and TF, respectively. All 40 parameters have been used in the integrative analysis; but the parameters “background intensity” and “colocalisation” at the end of the table have not been used for the summarized graphical representation. (PDF) [file pcbi.1003801.s020.pdf]

Parameter group label	Parameter group description	Detailed Parameter description
**G1**	Number of endosomes	(1, 19) number of endosomes
**G2**	Cargo uptake	(2, 20) total frame intensity<br><br>(3, 21) integral vesicular intensity
**G3**	Endosome area	(4, 22) mean endosome area weighted by volume<br><br>(5, 23) mean endosome area weighted by intensity<br><br>(6, 24) median endosome area
**G4**	Vesicle elongation	(7, 25) mean endosome elongation weighted by volume<br><br>(8, 26) mean endosome elongation weighted by intensity<br><br>(9, 27) median endosome elongation
**G5**	Endosomal cargo concentration	(10, 28) mean endosome intensity weighted by volume<br><br>(11, 29) mean endosome intensity weighted by intensity<br><br>(12, 30) median endosome intensity
**G6**	Endosomal cargo content	(13, 31) mean endosome integral intensity weighted by volume<br><br>(14, 32) mean endosome integral intensity weighted by intensity<br><br>(15, 33) median endosome integral intensity
**G7**	Endosome distance from nucleus	(16, 34) mean endosome distance weighted by volume<br><br>(17, 35) mean endosome distance weighted by intensity<br><br>(18, 36) median endosome distance
**-**	-	(37, 38) background intensity
**-**	-	(39) colocalisation of EGF to TF by volume
**-**	-	(40) colocalisation of TF to EGF by volume
