# Supplementary material for: Revealing Molecular Mechanisms by Integrating High-Dimensional Functional Screens with Protein Interaction Data
Source: PLoS Comput Biol. 2014 Sep 4;10(9):e1003801. doi: 10.1371/journal.pcbi.1003801 (PMC4154648; doi:10.1371/journal.pcbi.1003801)
Supplement: Table S2 — GO terms related to endocytosis. GO terms used for assembling the positive reference set. Genes annotated for one or more of these terms were considered as positives (387). The negative set was assembled considering genes that are annotated with functions other than endocytosis (21,585). Of those, 293 positive and 9,929 negative genes are represented in the network and 133 positive and 2,735 negative ones are in the sets. (PDF) [file pcbi.1003801.s021.pdf]

GO Term id	GO Term Name
**GO:0006897**	Endocytosis
**GO:0016197**	Endosome transport
**GO:0032439**	Endosome localization
**GO:0005768**	Endosome
**GO:0044440**	Endosomal Part
**GO:0030904**	Retromer Complex
**GO:0016471**	Vacuolar hydrogen-translocating V-type ATPase complex
