# Supplementary material for: Revealing Molecular Mechanisms by Integrating High-Dimensional Functional Screens with Protein Interaction Data
Source: PLoS Comput Biol. 2014 Sep 4;10(9):e1003801. doi: 10.1371/journal.pcbi.1003801 (PMC4154648; doi:10.1371/journal.pcbi.1003801)
Supplement: Table S3 — CORUM complexes. CORUM database comprehends manually curated protein complexes from Human and from other mammalian organisms. In the table the number of complexes per organisms is reported. The total number from all species is 2,083. (PDF) [file pcbi.1003801.s022.pdf]

Organisms	Number of complexes
**Bovine**	27
**Dog**	18
**Hamster**	2
**Human**	1343
**MINK**	1
**Mammalia**	94
**Mouse**	330
**Pig**	5
**Rabbit**	12
**Rat**	251
