# Supplementary material for: Revealing Molecular Mechanisms by Integrating High-Dimensional Functional Screens with Protein Interaction Data
Source: PLoS Comput Biol. 2014 Sep 4;10(9):e1003801. doi: 10.1371/journal.pcbi.1003801 (PMC4154648; doi:10.1371/journal.pcbi.1003801)
Supplement: Table S4 — Results of IMPACT-sets on CORUM complexes and IMPACT-modules on the combined interaction network. Classification performances are reported in terms of AUCs (of the ROC curves) of IMPACT-sets (left) and IMPACT-modules (right) applied on CORUM complexes and on the combined interaction network respectively. AUCs are reported for different similarity thresholds (T) and for the analysis based on a single profile (i.e. average of the original oligonucleotide profiles and mode of the original oligonucleotide, cases single-avg and single-mode). The table on the right shows AUCs obtained by using IMPACT-modules with different similarity thresholds (T) and different minimum number/percentage of profiles (k), labeled as T - k. Legend: AUC = area under the ROC curve; sem = standard error of the mean; p-values = p (AUC)>0.5. (PDF) [file pcbi.1003801.s023.pdf]

IMPACT-sets (complexes)			
**Parameters**	**AUC**	**sem**	**p-value**
**0.6**	0.578	0.029	0.004
**0.65**	0.611	0.030	1e-04
**0.7**	0.619	0.033	2e-04
**0.75**	0.602	0.038	0.004
**0.8**	0.497	0.043	0.472
**0.7-single-avg**	0.571	0.066	0.141
**0.7-single-mode**	0.636	0.070	0.026

IMPACT-modules (network)			
**Parameters**	**AUC**	**sem**	**p-value**
**0.6 - 3**	0.540	0.037	0.140
**0.65 - 3**	0.532	0.049	0.257
**0.7 - 3**	0.648	0.068	0.015
**0.75 - 3**	0.613	0.108	0.148
**0.8 - 3**	0.613	0.138	0.206
**0.7 - 50%**	0.789	0.068	1e-05
**0.7 - 30%**	0.548	0.042	0.127
**0.7-single-avg**	0.496	0.037	0.457
**0.7-single-mode**	0.528	0.040	0.242
**0.7 - 4**	0.551	0.176	0.386
**0.7 - 3**	0.648	0.068	0.015
**0.7 - 2**	0.553	0.030	0.039
**0.7 - 1**	0.506	0.019	0.376
