# Supplementary material for: Revealing Molecular Mechanisms by Integrating High-Dimensional Functional Screens with Protein Interaction Data
Source: PLoS Comput Biol. 2014 Sep 4;10(9):e1003801. doi: 10.1371/journal.pcbi.1003801 (PMC4154648; doi:10.1371/journal.pcbi.1003801)
Supplement: Table S5 — Number of genes selected by IMPACT. Number of unique genes identified by both IMPACT-sets (T = 0.7) and IMPACT-modules (T = 0.7, k = 2). Left column: total numbers of genes selected by IMPACT in any module/set. Right column: number of genes in modules/sets with p-values< = 0.1. 0.7-single-mode and 0.7-single-avg represent the analysis done by considering a single profile i.e. mode and average profile computed out of the original oligo profiles of each gene. (PDF) [file pcbi.1003801.s024.pdf]

Selection criteria	# genes selected	# genes p-value <= 0.1
**0.7**	4049	2720
**0.7–single-mode**	2376	2064
**0.7–single-avg**	2641	2400
