# Supplementary material for: Revealing Molecular Mechanisms by Integrating High-Dimensional Functional Screens with Protein Interaction Data
Source: PLoS Comput Biol. 2014 Sep 4;10(9):e1003801. doi: 10.1371/journal.pcbi.1003801 (PMC4154648; doi:10.1371/journal.pcbi.1003801)
Supplement: Table S6 — Classification results obtained considering protein complexes as interaction network. The table summarize the results obtained by running IMPACT-sets and IMPACT-modules on protein complexes, with k = 3 (minimal number of similar profiles). Isolated network: protein complexes are converted to binary protein interactions with the matrix model (all interacting with all) and they constitute an interaction network on their own. Other network context: as in isolated network, but the complex interaction network is added to the whole combined network used for IMPACT-modules. (PDF) [file pcbi.1003801.s025.pdf]

**Method**	**Case**	**AUC**	**sem**	**p(AUC) > 0.5**
IMPACT-sets	Complexes *T* = 0.7	0.619	0.033	2e-4
IMPACT-modules	Complex_as_network *T* = 0.7; *k* = 3 (isolated network)	0.531	0.052	0.2755
IMPACT-modules	Complex_as_network *T* = 0.7; *k* = 3 (other network context)	0.585	0.044	0.0264
