# Supplementary material for: Revealing Molecular Mechanisms by Integrating High-Dimensional Functional Screens with Protein Interaction Data
Source: PLoS Comput Biol. 2014 Sep 4;10(9):e1003801. doi: 10.1371/journal.pcbi.1003801 (PMC4154648; doi:10.1371/journal.pcbi.1003801)
Supplement: Table S7 — Classification results obtained considering different subsets of parameters in the phenotypic vector. Different groups of parameters (left column, Table S1 for details) were removed one by one from the original phenotypic data. G1 and G2 parameter groups were combined together because: 1) they represent linked biological features (endocytic uptake); 2) to have the same number of excluded parameters as the other cases (6 each). IMPACT-modules was run (T = 0.7, k = 3) and the AUC on the selected modules was measured for each case (right column). (PDF) [file pcbi.1003801.s026.pdf]

Parameter subset	AUC	sem	p(AUC) > 0.5
all params	0.648	0.0679	0.015
excluding G1-G2	0.556	0.0732	0.222
excluding G3	0.622	0.0532	0.011
excluding G4	0.514	0.0580	0.405
excluding G5	0.503	0.0696	0.483
excluding G6	0.632	0.0649	0.021
excluding G7	0.575	0.0493	0.064
