# Supplementary material for: Revealing Molecular Mechanisms by Integrating High-Dimensional Functional Screens with Protein Interaction Data
Source: PLoS Comput Biol. 2014 Sep 4;10(9):e1003801. doi: 10.1371/journal.pcbi.1003801 (PMC4154648; doi:10.1371/journal.pcbi.1003801)
Supplement: Table S8 — Comparison of classification performance of IMPACT-modules with other methods. In the table are reported Area Under the Curve (AUC) and standard error of the mean (sem) values relative to the analysis conducted with IMPACT-modules (grey rows) and relative to analysis performed using alternative methods (MATISSE, JActiveModules) that integrate network information with phenotypic data and are based on single profile (MATISSE) or on single values (JActiveModule). Chi-mode and Chi-avg denote the classification of the chi-square statistic calculated on the mode and average profile. Degree denotes the classification performances obtained by ranking the degree of network nodes. (PDF) [file pcbi.1003801.s027.pdf]

Method	AUC	sem
**0.7 – 3 profiles**	0.648	0.06
**0.7 – 2 profiles**	0.553	0.03
**MATISSE (mode prof.)**	0.518	0.017
**JActiveModules (mode prof.)**	0.507	0.018
**Chi-mode**	0.524	0.019
**Chi-avg**	0.518	0.018
**Degree**	0.516	0.30
