# Supplementary material for: Revealing Molecular Mechanisms by Integrating High-Dimensional Functional Screens with Protein Interaction Data
Source: PLoS Comput Biol. 2014 Sep 4;10(9):e1003801. doi: 10.1371/journal.pcbi.1003801 (PMC4154648; doi:10.1371/journal.pcbi.1003801)
Supplement: Table S9 — Comparison of classification performances of IMPACT-sets with other methods. Comparison of classification performances of IMPACT-sets with a different approach that uses only phenotypic information (e.g. chi-square statistic calculated on the mode and average profile of the oligo profiles of each gene). (PDF) [file pcbi.1003801.s028.pdf]

Method	AUC	sem
**IMPACT-sets (T=0.7)**	0.619	0.033
**Chi-mode**	0.553	0.029
**Chi-avg**	0.485	0.026
