# Supplementary material for: Revealing Molecular Mechanisms by Integrating High-Dimensional Functional Screens with Protein Interaction Data
Source: PLoS Comput Biol. 2014 Sep 4;10(9):e1003801. doi: 10.1371/journal.pcbi.1003801 (PMC4154648; doi:10.1371/journal.pcbi.1003801)
Supplement: Table S10 — Classification performances of IMPACT-modules and other methods on different positive sets. Classification performances of IMPACT-modules measured on different lists and compared to other methods. Lists: 1) endocytosis GO terms list (yellow); 2) RAB5 effectors and proteins with domains related to endocytosis (red); 3) same as 2), excluding genes common to GO (cyan); 4) the union of both 1) and 2) (green). Legend: AUC = area under the ROC curve; sem = standard error of the AUC estimation; p(AUC) = probability that the AUC is higher than the random 0.5 case (z-test); p(diff) = probability that difference between AUCs of the reference method (0.7–3 or 0.7–2) and the compared case is significant (z-test of differences of stratified bootstrapped values). (PDF) [file pcbi.1003801.s029.pdf]

GO				
**Method**	**AUC**	**sem**	**p(AUC) > 0.5**	**p(diff) 07-3, 07-2**
IMPACT-modules 0.7-3	0.6483	0.0679	0.0145	-, 0.0817
IMPACT-modules 0.7-2	0.5527	0.0295	0.0371	0.0817, -
JAM	0.5074	0.0176	0.3370	0.0162, 0.0726
Matisse	0.5184	0.0172	0.1433	0.0244, 0.1445
Chi-mode	0.5241	0.0189	0.1011	0.0315, 0.1935
Chi-avg	0.5177	0.0177	0.1576	0.0250, 0.1352
**RAB5_DOM**				
**Method**	**AUC**	**sem**	**p(AUC) > 0.5**	**p(diff) 07-3, 07-2**
IMPACT-modules 0.7-3	0.6735	0.071	0.0073	-, 0.0812
IMPACT-modules 0.7-2	0.5708	0.032	0.0135	0.0812, -
JAM	0.5205	0.0202	0.1555	0.0146, 0.0842
Matisse	0.5445	0.0204	0.0147	0.0371, 0.2533
Chi-mode	0.5664	0.0218	0.0012	0.0634, 0.4645
Chi-avg	0.5204	0.0202	0.1565	0.0134, 0.0943
**RAB5_DOM_not_GO**				
**Method**	**AUC**	**sem**	**p(AUC) > 0.5**	**p(diff) 07-3, 07-2**
IMPACT-modules 0.7-3	0.6460	0.0911	0.0545	-, 0.7665
IMPACT-modules 0.7-2	0.5792	0.0386	0.0201	0.2335, -
JAM	0.4992	0.0237	0.5138	0.0421, 0.0259
Matisse	0.5389	0.0242	0.0537	0.1137, 0.1797
Chi-mode	0.5610	0.0258	0.0090	0.1641, 0.3418
Chi-avg	0.5120	0.0239	0.3068	0.0639, 0.0625
**GO+RAB5_EFF**				
**Method**	**AUC**	**sem**	**p(AUC) > 0.5**	**p(diff) 07-3, 07-2**
IMPACT-modules 0.7-3	0.652	0.055	0.0028	-, 0.0614
IMPACT-modules 0.7-2	0.5643	0.0237	0.0034	0.0614, -
JAM	0.5045	0.0142	0.3759	0.0028, 0.0119
Matisse	0.5257	0.0142	0.0348	0.0102, 0.0763
Chi-mode	0.5378	0.0154	0.0071	0.0177, 0.1664
Chi-avg	0.5159	0.0143	0.1340	0.0050, 0.0321
