# Supplementary material for: Revealing Molecular Mechanisms by Integrating High-Dimensional Functional Screens with Protein Interaction Data
Source: PLoS Comput Biol. 2014 Sep 4;10(9):e1003801. doi: 10.1371/journal.pcbi.1003801 (PMC4154648; doi:10.1371/journal.pcbi.1003801)
Supplement: Table S11 — Enrichment list for network modules. List of molecular pathways (KEGG) enriched among the modules with low p-value<0.1. The enrichment was evaluated with the hypergeometric test by using the DAVID web-based Bioinformatics resources [3], [4]. The background distribution was built considering the entire network. (PDF) [file pcbi.1003801.s030.pdf]

KEGG Pathway Term	PValue	Benjamini
hsa04510:Focal adhesion	3.87E-25	7.32E-23
hsa05200:Pathways in cancer	4.96E-21	4.69E-19
hsa05220:Chronic myeloid leukemia	7.88E-17	6.99E-15
hsa04010:MAPK signaling pathway	1.18E-15	5.77E-14
hsa04370:VEGF signaling pathway	9.65E-15	3.65E-13
hsa04810:Regulation of actin cytoskeleton	1.39E-14	4.37E-13
hsa05212:Pancreatic cancer	3.03E-14	8.18E-13
hsa05215:Prostate cancer	3.17E-14	7.48E-13
hsa04070:Phosphatidylinositol signaling system	9.07E-14	1.90E-12
hsa04910:Insulin signaling pathway	4.20E-13	7.93E-12
hsa05218:Melanoma	4.67E-13	8.02E-12
hsa04012:ErbB signaling pathway	6.91E-13	1.09E-11
hsa05214:Glioma	2.61E-12	3.80E-11
hsa05223:Non-small cell lung cancer	5.56E-12	7.51E-11
hsa04630:Jak-STAT signaling pathway	7.79E-12	9.81E-11
hsa05210:Colorectal cancer	3.41E-11	4.02E-10
hsa04666:Fc gamma R-mediated phagocytosis	8.06E-11	8.96E-10
hsa04722:Neurotrophin signaling pathway	2.62E-10	2.75E-09
hsa05222:Small cell lung cancer	2.91E-10	2.89E-09
hsa04660:T cell receptor signaling pathway	3.23E-10	3.06E-09
hsa05221:Acute myeloid leukemia	9.41E-10	8.08E-09
hsa05211:Renal cell carcinoma	1.87E-09	1.47E-08
hsa05213:Endometrial cancer	3.82E-09	2.89E-08
hsa05219:Bladder cancer	8.26E-09	6.01E-08
hsa04662:B cell receptor signaling pathway	1.80E-08	1.26E-07
hsa04664:Fc epsilon RI signaling pathway	1.81E-08	1.22E-07
hsa04540:Gap junction	5.82E-08	3.79E-07
hsa04914:Progesterone-mediated oocyte maturation	6.40E-08	4.03E-07
hsa04912:GnRH signaling pathway	2.72E-07	1.60E-06
hsa04512:ECM-receptor interaction	3.96E-07	2.27E-06
hsa04114:Oocyte meiosis	1.10E-06	5.94E-06
hsa04110:Cell cycle	1.84E-06	9.64E-06
hsa04210:Apoptosis	3.45E-06	1.76E-05
hsa04062:Chemokine signaling pathway	4.02E-06	2.00E-05
hsa04520:Adherens junction	8.49E-06	4.11E-05
hsa04620:Toll-like receptor signaling pathway	2.51E-05	1.18E-04
hsa04020:Calcium signaling pathway	4.43E-05	1.99E-04
hsa04530:Tight junction	5.19E-05	2.28E-04
hsa04650:Natural killer cell mediated cytotoxicity	5.56E-05	2.39E-04
hsa04350:TGF-beta signaling pathway	5.98E-05	2.51E-04
hsa04150:mTOR signaling pathway	0.00109	0.003739
hsa04920:Adipocytokine signaling pathway	0.001325	0.004466
hsa05216:Thyroid cancer	0.001461	0.004752
hsa04310:Wnt signaling pathway	0.003391	0.00998
hsa04144:Endocytosis	0.003741	0.01084
hsa04115:p53 signaling pathway	0.015232	0.039491
