# Supplementary material for: Revealing Molecular Mechanisms by Integrating High-Dimensional Functional Screens with Protein Interaction Data
Source: PLoS Comput Biol. 2014 Sep 4;10(9):e1003801. doi: 10.1371/journal.pcbi.1003801 (PMC4154648; doi:10.1371/journal.pcbi.1003801)
Supplement: Table S12 — Complete list of endocytosis significant genes rescued by IMPACT. 92 endocytic genes (GO annotation) were found in significant modules and/or protein complexes (p-value< = 0.1) by IMPACT-sets (T = 0.7) and IMPACT-modules (T = 0.7, k = 2) and were not hits in the previous screen analysis [1]. * indicates genes in significant modules/sets. (PDF) [file pcbi.1003801.s031.pdf]

ABCG1	CRK*	LYST*	RAB9A	VLDLR*
AP1B1*	CTTN	NECAP2*	RAMP3	VPS13A*
AP1G1*	DBNL*	NEDD4L*	RAPGEF1*	VPS16
AP1G2	DNM1*	NOSTRIN*	SH3GL1	VPS28*
AP3B1*	EPN1*	NUMB*	SH3GL2*	VPS35
APOB*	EPN2*	PACSIN3*	SH3GL3*	VPS41
APP	FGF10*	PICALM*	SH3KBP1*	VPS4A
APPL1*	FLNA*	PLD2*	SLA*	VTI1A*
ARF6	FNBP1	PLDN*	SNX1*	WAS*
ARSA*	GGA2	PRKCI	SORL1	YKT6
ASGR1*	GJA1*	PRKCZ*	SPG21*	ZFYVE9*
ATP6V0A2*	GNPNAT1*	PSCD2	SQSTM1	
BACE1*	HIP1R	PTP4A1*	STX16	
BCAP31*	KIAA0368*	RAB11FIP1*	STX2*	
BET1L*	KRAS*	RAB11FIP2	STX7	
CBL*	LGMN	RAB18*	SYBL1*	
CD2AP*	LRP1*	RAB21*	SYNJ1*	
CD63	LRP1B*	RAB4A*	TSC2*	
CHMP1A*	LRP5*	RAB5A*	VAMP4*	
CLEC4F*	LRP6*	RAB5B*	VAMP8*	
