# Supplementary material for: Revealing Molecular Mechanisms by Integrating High-Dimensional Functional Screens with Protein Interaction Data
Source: PLoS Comput Biol. 2014 Sep 4;10(9):e1003801. doi: 10.1371/journal.pcbi.1003801 (PMC4154648; doi:10.1371/journal.pcbi.1003801)
Supplement: Table S13 — Comparison of classification performance of IMPACT-modules depending on similarity measure and seeding. In the table are reported Area Under the Curve (AUC), standard error of the mean (sem) and p-values (probability that the AUC is higher than the random 0.5 case; z-test) relative to the analysis comparing different similarity measures for both modules and sets (absolute versus positive correlation) and different seeding for modules only. When not indicated, IMPACT-modules is run with default seeding parameters T_s = 0.8 and k_s = 2, as reported in the main text. The cases in bold are the ones chosen for the follow-up analysis in the main text. (PDF) [file pcbi.1003801.s032.pdf]

Scenario	AUC	sem	p(AUC) > 0.5
IMPACT-sets with absolute correlation, T = 0.7	0.567	0.032	0.018
**IMPACT-sets with positive correlation, T = 0.7**	**0.619**	**0.033**	**2e-04**
**IMPACT-modules with absolute correlation, T = 0.7, k = 3**	**0.648**	**0.0681**	**0.015**
IMPACT-modules with positive correlation, T = 0.7, k = 3	0.6273	0.0682	0.031
**IMPACT-modules with absolute correlation, T = 0.7, k = 2**	**0.5527**	**0.0295**	**0.037**
IMPACT-modules with positive correlation, T = 0.7, k = 2	0.5395	0.0306	0.098
IMPACT-modules with different seeding (T_s = 0.5), T = 0.7, k = 3	0.5713	0.0433	0.050
IMPACT-modules with random seeding, T = 0.7, k = 3	0.5920	0.0754	0.111
IMPACT-modules with random seeding, T = 0.7, k = 2	0.5069	0.0305	0.411
