# Supplementary material for: Revealing Molecular Mechanisms by Integrating High-Dimensional Functional Screens with Protein Interaction Data
Source: PLoS Comput Biol. 2014 Sep 4;10(9):e1003801. doi: 10.1371/journal.pcbi.1003801 (PMC4154648; doi:10.1371/journal.pcbi.1003801)
Supplement: Table S14 — Comparison of different networks used as prior information in IMPACT-modules. Different networks used as prior information for IMPACT-modules. For STRING, different evidence levels (experimental, all and co-expression) and different confidence levels (400, 700 and all (> = 0), respectively) were used. Network features and module seeding/searching summary are reported. (PDF) [file pcbi.1003801.s033.pdf]

**Network**	**# genes network**	**# edges network**		**# seeds (T_s=0.8, k_s=2)**	**# modules (T=0.7, k=3)**
**HPRD-Intact-KEGG**	**10’305**	**53’810**		**4’357**	**297**
STRING experimental 0.4	11’526	76’394		5’441	413
STRING all 0.7	13’310	198’410		7’257	1’046
STRING co-expression all	14’374	695’627		9’426	2’580
