# Supplementary material for: Revealing Molecular Mechanisms by Integrating High-Dimensional Functional Screens with Protein Interaction Data
Source: PLoS Comput Biol. 2014 Sep 4;10(9):e1003801. doi: 10.1371/journal.pcbi.1003801 (PMC4154648; doi:10.1371/journal.pcbi.1003801)
Supplement: Table S15 — Classification performance of IMPACT-modules with different interaction networks on the endocytosis GO terms. Legend: AUC = area under the ROC curve; sem = standard error of the AUC estimation; p(AUC) = probability that the AUC is higher than the random 0.5 case (z-test). (PDF) [file pcbi.1003801.s034.pdf]

Method	Network	AUC	sem	p(AUC) > 0.5
**IMPACT-modules, T = 0.7, k = 3**	**HPRD+Intact+KEGG**	**0.6483**	**0.0679**	**0.0145**
IMPACT-modules, T = 0.7, k = 3	String exp. 0.4	0.6034	0.0512	0.0217
IMPACT-modules, T = 0.7, k = 3	String all 0.7	0.5525	0.0418	0.1046
IMPACT-modules, T = 0.7, k = 3	String co-expression	0.5046	0.0332	0.4449
