# Supplementary material for: Revealing Molecular Mechanisms by Integrating High-Dimensional Functional Screens with Protein Interaction Data
Source: PLoS Comput Biol. 2014 Sep 4;10(9):e1003801. doi: 10.1371/journal.pcbi.1003801 (PMC4154648; doi:10.1371/journal.pcbi.1003801)
Supplement: Table S16 — Enrichment analysis for the autophagy genes. Legend for columns, from left to right: scoring method; number of autophagy genes in the scoring list; total number of autophagy genes assessed by the scoring method (175 for the entire screen, of which 161 mapping on the interaction network); number of genes selected as significant by the scoring method; total number of genes assessed by the scoring method; p-value of enrichment of autophagy genes in the scoring hit-list, calculated through Fisher's exact test. # gene sig. = gene in significant modules (p-value< = 0.1). (PDF) [file pcbi.1003801.s035.pdf]

Method	# autophagy genes in list	# autophagy genes in total	# genes sig.	# total genes	p-value enrichment
IMPACT-modules, T = 2.5, k = 2	46	161	1332	6825	2e-3
IMPACT-modules, T = 3, k = 2	37	161	837	6825	4e-5
IMPACT-modules, T = 2, k = 2	75	161	2303	6825	1e-4
IMPACT-modules, avg. , T = 4, k = 1	65	161	1724	6825	7e-6
rank	25	175	1000	9481	0.04
