# Supplementary material for: Revealing Molecular Mechanisms by Integrating High-Dimensional Functional Screens with Protein Interaction Data
Source: PLoS Comput Biol. 2014 Sep 4;10(9):e1003801. doi: 10.1371/journal.pcbi.1003801 (PMC4154648; doi:10.1371/journal.pcbi.1003801)
Supplement: Table S17 — Classification performance for the autophagy genes. Legend: AUC = area under the ROC curve; sem = standard error of the AUC estimation; p(AUC) = probability that the AUC is higher than the random 0.5 case (z-test). Abbreviation: avg. = average of profiles from different replicates. (PDF) [file pcbi.1003801.s036.pdf]

**Method**	**AUC**	**sem**	**p(AUC) > 0.5**
IMPACT-modules, T = 2.5, k = 2	0.5633	0.0237	3.8e-3
IMPACT-modules, avg., T = 4, k = 1	0.5856	0.0238	2e-4
rank	0.4949	0.0299	0.5881
