# Supplementary material for: Revealing Molecular Mechanisms by Integrating High-Dimensional Functional Screens with Protein Interaction Data
Source: PLoS Comput Biol. 2014 Sep 4;10(9):e1003801. doi: 10.1371/journal.pcbi.1003801 (PMC4154648; doi:10.1371/journal.pcbi.1003801)
Supplement: Table S18 — Gene Ontology biological process annotations enriched in the CRISPR-Cas9 screen analysis. Column legend (left to right): name summarizing the different GO categories in the cluster; enrichment score, calculated as −Log (p-value), where p-values is the one in the next column; geometric mean of enrichment p-values for terms in the same cluster; geometric mean of the fold enrichment of different terms in the same cluster; geometric mean of the p-value corrected for multiple hypothesis (Benjamini correction). (PDF) [file pcbi.1003801.s037.pdf]

Annotation Cluster	Enrichment Score	Enrichment p-value (geometric mean)	Fold Enrichment (geometric mean)	Benjamini p-value (geometric mean)
Phosphorylation	10.89	1.30E-11	1.60	6.82E-09
Kinase activity / MAPK	8.30	4.97E-09	1.93	8.82E-07
Response to stimulus	8.05	8.85E-09	1.73	1.67E-06
Cell motility	6.04	9.18E-07	1.72	7.59E-05
Signal transduction	5.25	5.59E-06	1.75	3.27E-04
Cell growth	4.80	1.57E-05	1.96	7.29E-04
Transcription/biosynthesis	4.74	1.84E-05	1.52	8.46E-04
Immune response	4.41	3.89E-05	1.96	1.53E-03
Cell death / apoptosis	4.25	5.67E-05	1.47	2.03E-03
Cell morphogenesis	4.22	6.02E-05	1.69	2.12E-03
EGFR activity	3.80	1.58E-04	4.11	4.44E-03
