# Supplementary material for: Revealing Molecular Mechanisms by Integrating High-Dimensional Functional Screens with Protein Interaction Data
Source: PLoS Comput Biol. 2014 Sep 4;10(9):e1003801. doi: 10.1371/journal.pcbi.1003801 (PMC4154648; doi:10.1371/journal.pcbi.1003801)
Supplement: Table S19 — KEGG pathways annotations enriched in the CRISPR-Cas9 screen analysis. Column legend (left to right): name summarizing the different GO categories in the cluster; enrichment score, calculated as −Log (p-value), where p-values is the one in the next column; geometric mean of enrichment p-values for terms in the same cluster; geometric mean of the fold enrichment of different terms in the same cluster; geometric mean of the p-value corrected for multiple hypothesis (Benjamin correction). (PDF) [file pcbi.1003801.s038.pdf]

Annotation Cluster	Enrichment Score	Enrichment p-value (geometric mean)	Fold Enrichment (geometric mean)	Benjamini p-value (geometric mean)
Cancer pathways	6.25	5.68E-07	2.35	6.28E-06
Cell receptor	4.99	1.01E-05	1.97	7.75E-05
Cardiomyopathy	2.78	1.68E-03	1.73	6.73E-03
Linoleic acid metabolism	0.82	0.15	1.67	0.30
