# Supplementary material for: Revealing Molecular Mechanisms by Integrating High-Dimensional Functional Screens with Protein Interaction Data
Source: PLoS Comput Biol. 2014 Sep 4;10(9):e1003801. doi: 10.1371/journal.pcbi.1003801 (PMC4154648; doi:10.1371/journal.pcbi.1003801)
Supplement: Table S20 — Genes in the Melanoma KEGG pathway selected by IMPACT for the CRISPR-Cas9 screen analysis. Full list of the 35 genes selected by IMPACT belonging to the Melanoma KEGG pathway annotation. (DOCX) [file pcbi.1003801.s039.docx]

| **HGNC symbol** | | | |
| --- | --- | --- | --- |
| AKT2 | FGF21 | MET | PIK3R3 |
| AKT3 | FGF3 | MITF | PIK3R5 |
| CDH1 | FGF8 | NRAS | RAF1 |
| CDKN2A | FGFR1 | PDGFA | RB1 |
| E2F2 | HRAS | PDGFB | TP53 |
| EGF | IGF1 | PDGFD |  |
| EGFR | MAP2K1 | PDGFRB |  |
| FGF10 | MAPK1 | PIK3CB |  |
| FGF19 | MAPK3 | PIK3CD |  |
| FGF20 | MDM2 | PIK3R1 |  |
