# Supplementary material for: Revealing Molecular Mechanisms by Integrating High-Dimensional Functional Screens with Protein Interaction Data
Source: PLoS Comput Biol. 2014 Sep 4;10(9):e1003801. doi: 10.1371/journal.pcbi.1003801 (PMC4154648; doi:10.1371/journal.pcbi.1003801)
Supplement: Table S21 — Genes in the MAPK KEGG pathway selected by IMPACT for the CRISPR-Cas9 screen analysis. Full list of the 97 genes selected by IMPACT belonging to the MAPK KEGG pathway annotation. (DOCX) [file pcbi.1003801.s040.docx]

| **HGNC symbol** | | | | |
| --- | --- | --- | --- | --- |
| ACVR1C | EGFR | MAP3K7 | NTRK1 | RAPGEF2 |
| AKT2 | ELK4 | MAP3K8 | PAK1 | RASGRF1 |
| AKT3 | FGF10 | MAP4K1 | PAK2 | RASGRP1 |
| ATF2 | FGF19 | MAPK1 | PDGFA | RELA |
| BDNF | FGF20 | MAPK10 | PDGFB | RELB |
| CACNA2D2 | FGF21 | MAPK11 | PDGFRB | RPS6KA1 |
| CACNA2D4 | FGF3 | MAPK14 | PLA2G1B | RPS6KA2 |
| CACNB1 | FGF8 | MAPK3 | PLA2G2E | RPS6KA4 |
| CACNB4 | FGFR1 | MAPK8 | PLA2G2F | RPS6KA6 |
| CASP3 | FLNA | MAPK8IP3 | PLA2G3 | SOS2 |
| CHUK | FLNB | MAPT | PLA2G5 | STK3 |
| CRKL | GADD45G | MEF2C | PLA2G6 | TAB1 |
| DAXX | HRAS | MRAS | PPM1A | TGFB1 |
| DUSP1 | IKBKB | NFATC2 | PPP3CA | TGFB2 |
| DUSP2 | IL1R2 | NFATC4 | PRKACB | TP53 |
| DUSP3 | JUN | NFKB1 | PRKACG | TRAF2 |
| DUSP6 | MAP2K1 | NFKB2 | PRKX | TRAF6 |
| DUSP8 | MAP2K7 | NLK | PTPN7 |  |
| ECSIT | MAP3K1 | NRAS | PTPRR |  |
| EGF | MAP3K11 | NTF3 | RAF1 |  |
