# Supplementary material for: Revealing Molecular Mechanisms by Integrating High-Dimensional Functional Screens with Protein Interaction Data
Source: PLoS Comput Biol. 2014 Sep 4;10(9):e1003801. doi: 10.1371/journal.pcbi.1003801 (PMC4154648; doi:10.1371/journal.pcbi.1003801)
Supplement: Text S1 — Dissecting the function of related protein complexes. (PDF) [file pcbi.1003801.s041.pdf]

Dissecting the function of related protein complexes

SNARE complexes are critically involved in regulating the fusion of many cellular membranes [4]. This family of complexes consists of several variants composed of common as well as specific components localized to different sub-cellular compartments. This functional promiscuity likely resulted in relatively weak and divergent phenotypes for individual genes, which hampered the previous detection of those genes [1]. Despite these complications, our analysis identified consistent profiles for various SNARE complexes (Figure 4). For example, a complex consisting of STX4, SNAP23 and VAMP8 has a phenotype of high intracellular accumulation of transferrin at the cell periphery, consistent with impaired fusion of recycling tubules with the plasma membrane [3,4].

Endosomal sorting complexes required for transport (ESCRT) are important for the degradation of endocytosed membrane proteins and for the biogenesis of multi-vesicular bodies [6,7]. ESCRTs are recruited in a cascade beginning with ESCRT-0, followed by ESCRT-I, -II and –III. Silencing components of either ESCRT-0, -I or –II impacts on both EGF and transferrin (Figure 4). Whereas loss of ESCRT-0 and -I decreases cargo content and cargo concentration of endosomes, knock-down of ESCRT-II has the opposite effect. Thus, even though ESCRT complexes have related functions and act in a linear pathway for the generation of MVB, they also have other endocytic functions leading to heterogeneous phenotypes, as previously suggested [8].
