# Supplementary material for: Revealing Molecular Mechanisms by Integrating High-Dimensional Functional Screens with Protein Interaction Data
Source: PLoS Comput Biol. 2014 Sep 4;10(9):e1003801. doi: 10.1371/journal.pcbi.1003801 (PMC4154648; doi:10.1371/journal.pcbi.1003801)
Supplement: Text S2 — Application to other data sets. (DOCX) [file pcbi.1003801.s042.docx]

## Application to other data sets

### Other sources of prior information

We run IMPACT-modules on different interaction networks obtained from the STRING database (<http://string-db.org>, [10]). STRING is a database of known and predicted protein interactions, including direct (physical) and indirect (functional) associations. Those are derived from different sources, called evidence: biochemical experiments, high-throughput screens, co-expression, evolutionary analysis and previous knowledge (e.g. text mining and databases). Each protein interaction is reported with a quantitative confidence level, a score between 0 (low probability) and 1 (high probability) that indicates the certainty of that interaction. Confidence scores are reported individually for each different evidence type and as a cumulated confidence value.

We built 3 different interaction networks by considering different evidence (experimental, all, co-expression) and different confidence levels (≥400, ≥700, all, respectively). Then, we mapped the phenotypic data in the networks, obtaining different coverage in terms of nodes and edges represented (Table S14). We then run IMPACT-modules with the parameters of our best performing case (i.e., seeding with *T_s* = 0.8 and *k_s* = 2; module expansion with *T* = 0.7 and *k* = 3) and we compared the results to the previously described HPRD-Intact-KEGG high quality network used in this study.

The results of the comparison suggested that, once the search parameters are fixed, the number of seed nodes examined and modules identified strongly depended on the number of edges in the network (Table S14). However, when comparing the classification performance on the endocytosis GO terms list (AUC), we noticed that the choice of high-quality, experimentally validated interactions is preferable (Table S15), as the addition of uncertain or wrong edges between genes could confound module expansion and lead to the identification of spurious genes.

### Analysis of the siRNA autophagy screen

We analyzed the data from a genome-wide siRNA screen [11] performed in a stable human cell line (HEK 293) expressing GFP-LC3, the marker protein for autophagosomes. The aim of this study was to identify new proteins that modulate starvation-induced autophagy. The screen was performed in 3 independent replicates of the same siRNA pools. For each replicate, a set of 3 parameters was measured by automated image analysis, describing different features of autophagosomes, i.e. spot count per object (SCPO), spot total intensity per object (STIPO) and spot total area per object (STAPO). The data was then z-normalized within each run. After filtering for cell viability, three-dimensional vectors (i.e. 3 parameters) for 9’481 genes could be mapped uniquely to Ensemble ids, and among those 8’370 had all 3 replicates. We kept also the remaining 1’100 genes with 2 replicates as our method can handle different number of profiles per gene.

To run IMPACT-modules on this dataset, we also modified the procedure in order to use as similarity measure the inverse of the Euclidean distance instead of the Pearson correlation coefficient. In fact, we noticed that in a low-dimensional space the latter metric did not allow discriminating well among profiles that were visually similar versus dissimilar. On the other hand, in a high-dimensional space, Euclidean distance does not capture well similarity due to sparse data points, and therefore Pearson correlation is more adequate there. We modified the method to allow choosing between the two metrics.

We run IMPACT with different parameters (see Table S16) on 2 data sets: the full data set with different profiles per gene and a condensed version for the data set where we calculated an average profile per gene. We compared the results of IMPACT to the screening analysis, where 1’000 hit genes (500 increaser and 500 decreasers) were identified based on the combined ranking on the 3 parameters, i.e. the geometrical mean of the ranking of the three median parameters (median of 3 replicates).

For the comparison, we chose as positive set the 230 genes from the human autophagy database ([www.autophagy.lu](http://www.autophagy.lu), [12]), which we will call autophagy genes. Of those, 175 were screened and 161 mapped on our HPRD-Intact-KEGG interaction network. First, we analyzed if the genes identified by different methods were enriched in autophagy genes, by performing the hypergeometric test for enrichment. All method successfully enriched for autophagy genes (Table S16), but IMPACT was better (p-values < 2e-3 for all cases tested) than the ranking analysis (p-value = 0.04). Also, when considering two hit lists of the same length by selecting the top 1’332 ranking genes, IMPACT selected a higher proportion of autophagy genes (46 out of 6825 versus 32 out of 9481, p-value = 3e-3 with non-parametric chi-squared test for proportions). The classification performance analysis, where we measured the AUC of the ROC curves with the different methods, also showed better results for IMPACT compared to the ranking analysis (AUC of 0.563 versus 0.495; Table S17), suggesting that our method not only enriches for the expected genes in the positive set, but also attributes a lower p-value to them, i.e. autophagy genes are in more significant modules compared to non autophagy genes.

Differently from what was observed in the case of profiles coming from different siRNA reagents, when running IMPACT on the averaged profiles of three identical replicates (i.e., screen repetitions with the same set of siRNA pools), both the enrichment statistics and the classification performance were comparable and even slightly higher than the case with all profiles (Tables S16 and S17), suggesting that in this case averaging effectively suppressed parts of the noise. However, both differences were not statistically significant.

Finally, gene annotation enrichment analysis performed by the DAVID website [3,4] identified several molecular pathways and biological processes enriched for the 1’332 genes selected by IMPACT, whereas the ranking analysis did not identify any significant term after correction of multiple hypothesis testing (Supplementary Dataset S1). Among those, we could identify several autophagy-related processes, such as ‘regulation of apoptosis’, ‘catabolic processes’ and ‘hydrolase activity’ (p-values of 1.6e-5, 6.8e-3, 7.4e-3 respectively).

### Analysis of the CRISPR-CAS9 knockout screen

We analyzed the data from a genome-wide CRISPR-Cas9 knockout screen in human cells [13]. We considered the data set relative to the melanoma cell line A375, where the authors investigated the effect of gene loss upon treatment with vemurafenib (PLX), a therapeutic drug inhibitor of BRAF. In order to quantify the interaction between the drug and gene loss, the authors measured cell viability under the following conditions: only transfection plasmid; only vehicle (DMSO) treatment at 7 and 14 days; PLX drug treatment at 7 and 14 days. Each cell viability count was performed in 2 replicates (except for plasmid). For each condition, they performed gene knockout with several (2 to 5) synthetic single guide RNAs (sgRNAs), which by specific integration into the genome induce double strand break and frame shift indel mutations, resulting in a loss-of-function phenotype. The screening analysis reported in the paper was performed by comparing cell viability at 14 days in drug treatment versus control by using the RNAi Gene Enrichment Ranking (RIGER) algorithm to rank genes with consistent sgRNAs effect.

In order to run IMPACT on this data set, we tested different options: 1) we averaged different replicate counts for each condition (DMSO 7 days, DMSO 14 days, PLX 7days, PLX 14 days) and we calculated the log fold change of these values relative to the plasmid count, resulting therefore in a 4-dimensional phenotypic vector for each sgRNA; 2) same as 1), but we normalized the DMSO 7 days to zero, to subtract any offset due to the vehicle; 3) we subtracted PLX and DMSO counts at 7 and 14 days, resulting in a 2-dimensional vector that displays the variation upon drug treatment over the two measured time points. Then, we run IMPACT-modules using as similarity measure the inverse of the Euclidean distance (similar to the previous application in the autophagy screen) on all the described phenotypic data sets, and identified modules and significant genes (lists of genes in Supplementary Dataset S2). For the case with 2 parameters discussed below, IMPACT-modules identified 1418 significant genes.

We then performed gene annotation enrichment analysis by using the DAVID website, and compared the results of IMPACT to the RIGER analysis from the original publication. Table S18 and S19 show the enrichment clusters obtained with IMPACT (phenotypic data set with 2 parameters) for GO biological processes and KEGG pathways, respectively. Overlaying hit genes on a graphical representation of two of the most enriched KEGG pathways, the Melanoma and MAPK, shows that several core components and upstream receptors are affected by gene knockout (Table S20 and S21), revealing potential important mechanism of PLX intracellular action and resistance to the drug treatment. RIGER analysis performed on the top 1420 ranking genes showed enrichment for different categories with overall higher p-values (GO clusters ‘transcription’, ‘rRNA processing’, ‘histone modification’ and ‘oxidative phosphorylation’; p-values of 8e-4, 1e-3, 3e-3 and 1e-4 respectively). Overall, IMPACT identified more meaningful terms according to the known mechanisms of action of the drug. However, RIGER analysis was still useful to identify isolated genes (i.e. not having the same phenotype as their interactors) showing a decrease of cell viability, and therefore potentially involved in mechanisms of drug resistance (see *Discussion*). The complete enrichment results for IMPACT-modules with 4 parameters, IMPACT with 2 parameters and RIGER analysis are reported in Supplementary Dataset S2.
