# Supplementary material for: Revealing Molecular Mechanisms by Integrating High-Dimensional Functional Screens with Protein Interaction Data
Source: PLoS Comput Biol. 2014 Sep 4;10(9):e1003801. doi: 10.1371/journal.pcbi.1003801 (PMC4154648; doi:10.1371/journal.pcbi.1003801)
Supplement: Text S3 — References for the supplementary materials. (PDF) [file pcbi.1003801.s043.pdf]

## References for Supplementary Materials:


1. Collinet C, Stöter M, Bradshaw CR, Samusik N, Rink JC, et al. (2010) Systems survey of endocytosis by multiparametric image analysis. Nature 464: 243–249.

2. Christoforidis S, Zerial M (2000) Purification and identification of novel Rab effectors using affinity chromatography. Methods 20: 403–410. doi:10.1006/meth.2000.0953.

3. Huang DW, Sherman BT, Lempicki RA (2009) Systematic and integrative analysis of large gene lists using DAVID bioinformatics resources. Nat Protoc 4: 44–57. doi:10.1038/nprot.2008.211.

4. Fasshauer D, Antonin W, Margittai M, Pabst S, Jahn R (1999) Mixed and non-cognate SNARE complexes. Characterization of assembly and biophysical properties. J Biol Chem 274: 15440–15446.

5. Kennedy MJ, Davison IG, Robinson CG, Ehlers MD (2010) Syntaxin-4 defines a domain for activity-dependent exocytosis in dendritic spines. Cell 141: 524–535. doi:10.1016/j.cell.2010.02.042.

6. Slagsvold T, Pattni K, Malerød L, Stenmark H (2006) Endosomal and non-endosomal functions of ESCRT proteins. Trends Cell Biol 16: 317–326. doi:10.1016/j.tcb.2006.04.004.

7. Hurley JH, Hanson PI (2010) Membrane budding and scission by the ESCRT machinery: it's all in the neck. Nat Rev Mol Cell Biol 11: 556–566. doi:10.1038/nrm2937.

8. Raiborg C, Malerød L, Pedersen NM, Stenmark H (2008) Differential functions of Hrs and ESCRT proteins in endocytic membrane trafficking. Exp Cell Res 314: 801–813. doi:10.1016/j.yexcr.2007.10.014.
